# Apple replant disease: unraveling the fungal enigma hidden in the rhizosphere

**DOI:** 10.1007/s44154-025-00258-1

**Published:** 2025-11-27

**Authors:** Ziqing Ma, Yiwei Jia, Zhiquan Mao, Fengwang Ma, Qingmei Guan, Yanan Duan

**Affiliations:** 1 State Key Laboratory for Crop Stress Resistance and High-Efficiency Production/Shaanxi Key Laboratory of Apple, College of Horticulture, Northwest A&F University, Yangling , Shaanxi, 712100 China; 2 College of Horticultural Science and Engineering/State Key Laboratory of Crop Biology, Shandong Agricultural University, Tai′an, Shandong, 271018 China

**Keywords:** Apple replant disease, Fungal community, *Fusarium*, Illumina sequencing, Rhizosphere, Soil environment

## Abstract

The occurrence of apple replant disease (ARD) is closely related to the increase of soil pathogenic fungi abundance. However, the relationship between ARD and fungal community structure remains poorly understood. In this study, Illumina high-throughput sequencing was used to investigate the composition, diversity, and function of rhizosphere fungal communities associated with healthy (HRS) and diseased apple trees (DRS). Microbial taxa related to ARD were also identified. The severity of ARD varied among the sampled orchards. We found that Ascomycota was the dominant phylum in the DRS fungal taxa, and the fungal community abundance and Simpson index of DRS were significantly higher than those of HRS. Cluster and FUNGuild database analyses revealed significant differences in the relative abundance and function of fungal taxa between DRS and HRS. Most fungi isolated from DRS were plant pathogens, predominantly from the genus *Fusarium* (Ascomycota, Nectriaceae), which was also the predominant fungal genus detected in DRS. In contrast, *Mortierella* was more abundant in HRS. To validate the sequencing results, *Fusarium* isolates, including *F. proliferatum*, *F. oxysporum*, and *F. solani*, were verified as pathogens and showed high virulence. Structural equation modeling indicated that the occurrence of ARD was directly or indirectly influenced by *Fusarium*, *Mortierella*, phloridin, available phosphorus, and soil organic matter. Further research is needed to elucidate how soil parameters affect ARD. Laboratory tests demonstrated that *F. proliferatum* MR5 can produce pectinase and cellulase and is sensitive to two fungicides: flusilazole and bromothalonil. In conclusion, the deterioration of rhizosphere fungal community structure may be a key biological factor driving ARD, with *Fusarium* in DRS identified as a major causative agent of ARD in China. The findings of this study provide valuable insights for developing preventive strategies against ARD.

**Supplementary information:**

The online version contains supplementary material available at 10.1007/s44154-025-00258-1.

## Introduction

In China, the areas for apple cultivation are mainly located in the Northwest Loess region (NL) and the around Bohai Gulf region (ABG). More than half of the old apple orchards (over 20 years old) urgently require renovation and replanting in their original locations, leading to the widespread occurrence of apple replant disease (ARD). This has become a major obstacle to sustainable apple cultivation in the region (Duan et al. 2022; Li et al. 2018). ARD is a soil-disease syndrome of complex etiology that affects apple tree roots in replanted orchards, causing water and nutrient stress, stunted tree growth, necrosis of fine feeder roots, and reduced yields. These symptoms are especially severe in young apple trees and may even result in tree death (Jiang et al. 2017; Tilston et al. 2020). Previous studies have found that microbial community structure imbalances in apple tree rhizospheric soil, harmful physicochemical parameters, and autotoxic compound accumulation are the main reasons for the occurrence of ARD (Manici et al. 2003; Tilston et al. 2020; Yin et al. 2016). Among these, an imbalance in rhizospheric soil microbial communities has been identified as the primary disease-causing factor (Wang et al. 2021). Furthermore, several recent surveys have shown that plant growth and vitality in native soil are significantly reduced compared to pasteurized or fumigated soil, further highlighting the critical role of root-colonizing soilborne fungi as casual agents of replant disease (Jiang et al. 2017; Manici et al. 2003; Wang et al. 2024; Xiang et al. 2021b). Therefore, understanding the specific etiology of ARD in China is crucial for developing commercially viable soil management strategies to target specific/individual components of the disease.

Rhizosphere microbial diversity and ecological functions affect nutrient cycling, decomposition, and plant health (Turner et al. 2013). Fungi are considered the main decomposers in soil and are closely related to the development and transmission of soilborne diseases (Frąc et al. 2018). It has been reported that continuous cropping or monoculture can lead to an increase in the abundances of pathogenic microorganisms such as *Nectria*, *Rhizoctonia*, *Pythium*, *Cylindrocarpon*, and *Fusarium,* a reduction in beneficial microorganisms, an imbalance in microbial biota, ultimately affecting plant growth (Franke-Whittle et al. 2015; Gao et al. 2019; Manici et al. 2003; Tilston et al. 2020; Wang et al. 2018). In other words, when plant-induced changes in soil microbiota promote the invasion of multiple host-specific soilborne pathogens (fungi, oomycetes, and nematodes), apple replanting disease (ARD) occurs. This disease is caused by the combined effects of several pathogenic soil microorganisms rather than by a single pathogen acting alone (Somera and Mazzola 2022). In addition, environmental factors (e.g., phenolic acids, soil nutrition) may also shape the fungal community and its abundance in rhizospheric soil (Fei et al. 2021; Jiang et al. 2017; Song et al. 2018; Wang et al. 2012). Due to the involvement of multiple biological agents (some of which act synergistically) and abiotic factors (such as the accumulation of phenolic detritus from previous orchard plantings) in the occurrence of replanting diseases, their major functional roles have been difficult to establish. Therefore, clarifying the relationship between environmental factors and ARD-related pathogenic microorganisms can provide a theoretical basis for the control of ARD in China.

Various techniques have been employed to manage ARD, such as soil replacement, crop rotation, heat/radiation treatment, organic matter amendment, biological control, and the selection of ARD-resistant planting materials (Leinfelder and Merwin 2006; Mazzola and Manici 2012; Van Schoor et al. 2009; Wang et al. 2021; Zhao et al. 2014). However, these control measures face challenges such as high cost, complex operation, unclear mechanisms, and unstable effectiveness (Duan et al. 2021; Reim et al. 2019; Somera and Mazzola 2022). Chemical fungicides can effectively control or kill various harmful microorganisms in the soil and remain the primary means of controlling ARD, including chloropicrin, metham sodium, dazomet, calcium cyanamide, 1,3-dichloropropene, and methyl bromide (Ali et al. 2024; Jiang et al. 2020, 2022; Sabatino et al. 2019; Wang et al. 2016). However, methyl bromide has been banned due to its high toxicity and ozone-depleting effects (Raymaekers et al. 2020), and several other chemicals also pose environmental pollution risks and threaten human health (Yao et al. 2006). Research indicates that the repeated use of high-concentration chemical fungicides by farmers promotes the evolution of resistance in plant pathogenic fungi, thereby reducing the efficacy of these fungicides (Hahn 2014). Therefore, evaluating the resistance of pathogenic soil microorganisms to commonly used fungicides can provide new insights for developing and selecting low-toxic, high-efficiency fungicides to control ARD.

To address the above questions, we conducted a study on the symptoms of replant disease observed after the replacement of old orchards in the two main apple-growing regions of China: the around Bohai Gulf (ABG) and the Northwest Loess (NL) regions. Previous attempts at replanting in some old orchards revealed severe replant disease symptoms, including stunted growth and plant death. Therefore, using the Illumina MiSeq platform, we performed sequencing analysis of the rhizosphere fungal community (RFC) in symptomatic and asymptomatic apple trees. The main objectives were as follows: (1) to investigate the response of the soil fungal community in symptomatic and asymptomatic apple trees to ARD; (2) to isolate and culture potential pathogenic fungi from root and rhizospheric soil samples; (3) to define the interactions between fungal groups and soil properties, and (4) to provide a research basis for screening biological control agents to manage ARD.

## Results

### The rhizosphere soil environment

The rhizosphere soil in both regions (ABG and NL) was weakly acidic, with a sandy loam texture, except for XJ, XQ, and XC orchards, which had a silty clay loam texture (Tables S1 and S13). In the ABG region, the available phosphorus (AP) content ranged from 82.92 to 351.17 mg·kg⁻^1^, available potassium (AK) from 68.67 to 234.94 mg·kg⁻^1^, soil organic matter (SOM) from 1.23% to 2.94%, bulk density (ρb) from 0.97 to 1.44 g·cm⁻^3^, available nitrogen (AN) from 22.58 to 42.47 mg·kg⁻^1^, and water content (ω) from 6.36% to 16.96% in the rhizosphere soil of ARD symptomatic trees. In the NL region, AP ranged from 1.24 to 24.52 mg·kg⁻^1^, AK from 106.82 to 254.03 mg·kg⁻^1^, SOM from 1.04% to 2.08%, ρb from 1.06 to 1.28 g·cm⁻^3^, AN from 15.95 to 27.64 mg·kg⁻^1^, and ω from 6.20% to 14.08%. The content of AP and AN in the NL region was significantly lower than that in the ABG region. However, there were no statistically significant differences in the physicochemical properties of the rhizosphere soil associated with healthy and ARD symptomatic trees (Tables S13 and S14).

The contents of benzoic acid (Ba), phlorizin (Pd), syringic acid, and total phenolic acid in replanted orchards were higher than those in old apple orchards, with syringic acid (*P* = 0.0185) reaching significant levels. There were statistically significant differences in the content of Ba (*P* = 0.0092), *p*-hydroxybenzoic acid (Pha) (*P* = 0.0534), cinnamic acid (Ca) (*P* = 0.0058), Pd (*P* < 0.0001), and total phenolic acid (*P* = 0.0002) in the rhizosphere soil associated with healthy and ARD symptomatic trees (Table 1, Table S15).

**Table 1:** Selected phenolic acids in the rhizosphere soil of healthy trees without obvious symptoms of apple replant disease (ARD) and trees exhibiting symptoms attributed to ARD^a^

Physico-chemical property	Phloridzin (mg·100 g^−1^)			Benzoic acid (mg·100 g^−1^)			*p*-hydroxybenzoic acid (mg·100 g^−1^)			Syringate (mg·100 g^−1^)		
Location number^b^	ARD symptomatic	Healthy	*P* value	ARD symptomatic	Healthy	*P* value	ARD symptomatic	Healthy	*P* value	ARD symptomatic	Healthy	*P* value
DC	2.23 (0.37)	1.42 (0.14)	0.0244	6.22 (0.22)	4.85 (0.25)	0.0020	0.41 (0.01)	0.45 (0.07)	0.4003	2.47 (0.45)	1.99 (0.31)	0.1966
QC	1.07 (0.12)	0.71 (0.15)	0.0309	4.24 (0.33)	3.18 (0.52)	0.0403	0.51 (0.17)	0.28 (0.05)	0.0800	1.77 (0.12)	1.61 (0.31)	0.4536
LC	2.11 (0.47)	0.89 (0.07)	0.0115	10.46 (1.19)	7.54 (1.13)	0.0370	0.75 (0.02)	0.81 (0.09)	0.3028	2.02 (0.05)	1.74 (0.09)	0.0096
MC	1.13 (0.20)	0.60 (0.16)	0.0219	16.66 (0.72)	15.32 (1.09)	0.1493	0.65 (0.11)	0.43 (0.11)	0.0633	0.86 (0.12)	0.60 (0.20)	0.1289
JC	1.53 (0.15)	1.06 (0.11)	0.0116	4.58 (0.72)	3.65 (0.156)	0.0916	0.29 (0.11)	0.23 (0.09)	0.5578	1.49 (0.11)	1.48 (0.03)	0.9304
PC	1.05 (0.15)	0.68 (0.15)	0.0398	7.26 (0.93)	5.35 (0.38)	0.0297	0.63 (0.16)	0.68 (0.06)	0.6870	0.91 (0.07)	0.65 (0.18)	0.0811
YL	1.76 (0.05)	1.59 (0.11)	0.0665	6.69 (0.34)	4.60 (0.78)	0.0132	0.55 (0.06)	0.41 (0.09)	0.0972	0.69 (0.03)	0.56 (0.02)	0.0031
DL	1.44 (0.04)	0.99 (0.08)	0.0010	4.65 (0.37)	2.52 (0.12)	0.0007	0.62 (0.09)	0.49 (0.16)	0.2705	0.55 (0.13)	0.44 (0.05)	0.2358
HC	3.44 (0.23)	2.55 (0.04)	0.0026	7.20 (0.15)	6.96 (0.14)	0.0995	0.95 (0.04)	0.75 (0.10)	0.0277	3.39 (0.19)	2.86 (0.25)	0.0422
H	1.04 (0.22)	0.63 (0.05)	0.0363	15.98 (1.71)	11.64 (0.01)	0.0117	0.56 (0.09)	0.60 (0.05)	0.5874	1.14 (0.07)	0.97 (0.14)	0.1357
XQ	2.55 (0.09)	1.54 (0.23)	0.0019	13.70 (1.31)	7.80 (1.31)	0.0053	0.37 (0.02)	0.40 (0.04)	0.2849	1.49 (0.37)	1.36 (0.45)	0.7245
XC	1.28 (0.24)	0.68 (0.25)	0.0389	4.31 (0.51)	3.71 (0.30)	0.1550	0.36 (0.02)	0.28 (0.03)	0.0129	0.59 (0.10)	0.55 (0.15)	0.7078
XJ	0.87 (0.11)	0.53 (0.04)	0.0064	13.42 (2.29)	7.39 (0.10)	0.0103	0.28 (0.04)	0.26 (0.02)	0.5126	1.36 (0.18)	1.35 (0.29)	0.9492
XL	1.25 (0.23)	0.86 (0.09)	0.0505	3.09 (0.52)	2.55 (0.27)	0.1868	0.28 (0.06)	0.19 (0.08)	0.2083	0.88 (0.08)	0.63 (0.2)	0.1183
XF	1.39 (0.18)	0.95 (0.10)	0.0209	11.10 (0.78)	9.88 (0.50)	0.0859	0.44 (0.12)	0.18 (0.04)	0.0238	0.34 (0.05)	0.20 (0.05)	0.0237
XW	1.93 (0.91)	0.43 (0.03)	0.0470	7.70 (0.77)	5.91 (0.38)	0.0227	0.29 (0.07)	0.19 (0.07)	0.1458	1.22 (0.15)	1.07 (0.20)	0.3822

^a^All values are the mean of three replicates with the standard deviation of mean given in parentheses

^b^The specific meaning of the number can be found in Supplementary Table 1

ARD severity was calculated as the ratio of dry biomass produced in sterilized and unsterilized soil of the same orchard, and the soils were classified into severe, moderate, and low severity. Three of the sixteen soils were ranked as severe, 9 as moderately severe, and only 4 as mildly severe (Table 2).

**Table 2:** The ARD severity of soils

Location number^a^	Dry weight of sterilized soil plant (g)	Dry weight of unsterilized soil plant (g)	Dry weight inhibition rate (%)	The ARD severity of the soils	Severity classification of ARD^b^
DC	22.21 ± 0.05	11.49 ± 0.50	93.34	Moderate	II
QC	24.02 ± 0.19	16.61 ± 0.90	44.64	Low	III
LC	24.68 ± 0.24	12.05 ± 0.48	104.89	severe	I
MC	21.47 ± 0.34	14.60 ± 0.43	47.03	Low	III
JC	24.38 ± 0.30	15.06 ± 0.13	61.91	Moderate	II
PC	21.98 ± 0.90	13.65 ± 0.94	61.05	Moderate	II
YL	27.40 ± 0.41	14.07 ± 0.43	94.72	Moderate	II
DL	22.43 ± 0.37	11.31 ± 0.36	98.37	Moderate	II
HC	26.42 ± 0.21	12.73 ± 0.25	107.59	severe	I
H	19.52 ± 1.34	13.58 ± 1.28	43.76	Low	III
XQ	24.13 ± 0.22	11.83 ± 0.24	104.00	severe	I
XC	20.59 ± 1.13	13.33 ± 0.72	54.52	Moderate	II
XJ	17.05 ± 0.70	13.80 ± 0.90	23.57	Low	III
XL	21.44 ± 0.47	14.13 ± 0.39	51.75	Moderate	II
XF	21.63 ± 0.28	13.36 ± 0.28	61.84	Moderate	II
XW	23.48 ± 0.42	12.17 ± 0.24	92.93	Moderate	II

^a^The specific meaning of the number can be found in Supplementary Table 1

^b^ARD severity: severe (I), inhibition of dry weight > 100%; moderate (II), 100% > inhibition of dry weight > 50%; low (III), inhibition of dry weight < 50%

### Sequencing data statistics

Fungal ITS sequencing was performed on 78 rhizosphere soil samples from replanted orchards, yielding a total of 3,619,232 valid sequences. After filtering and optimizing, the number of valid sequences was reduced to 3,391,533. The proportions of optimized sequences exceeded 79.21%, with lengths predominantly ranging between 200 and 400 bp (Table S16). The rarefaction curves demonstrated that as the number of sequences increased, the curves initially rose sharply and then began to plateau at around 20,000 sequences, indicating that the sequencing results were reliable and suitable for analyzing the RFC structure (Fig. S2 A, D, G). Consequently, the rank-abundance distribution curves were used to illustrate species abundance and uniformity (Fig. S2 B, E, H). Along the X-axis, the species abundance in the rhizosphere soil of replanted orchards in the ABG region was higher than that in the NL region, while the uniformity of species distribution was similar between the two regions.

The species accumulation curves showed that as the sample size increased, the curves rose sharply before leveling off for all samples. This pattern indicated that the sampling effort was sufficient, and further data analysis could be conducted (Fig. S2 C, F, I).

### Diversity analysis of fungal communities

The Sobs, Chao, and ACE indexes reflected the richness of RFC, whereas the Shannon and Simpson indexes reflected the diversity. As shown in Table S17, the richness and diversity of RFC in the replanted orchards in the NL region were significantly lower than those in the ABG region. For the ABG region, the richness and diversity indices of the HRS and DRS fungal communities in the three orchards (HC, MC, and LC) showed no significant difference, and the fungal community richness and Simpson index were greater in DRS than in HRS. In addition, the Shannon index showed no significant differences.

According to Fig. S3 A-C, T-ABG (A), J-ABG (B), and T-NL(C) contained all unique OTUs, and the proportions of core OTUs were not significantly different. Notably, the total number of OTUs in T-ABG was greater than that in J-ABG, and the total number of OTUs in DRS was greater than that in HRS in the ABG region, except for HC and YL (Fig. S3 D-G, I-O). The number of unique OTUs in T-ABG was 3.01 times that in NL (Fig. S3 H).

### Rhizospheric biotic and abiotic factors associated with plant growth in the ABG and NL regions

At the phylum level, Ascomycota exhibited the highest relative abundance, followed by Basidiomycota and Zygomycota (Fig. S4). Linear regression revealed a negative correlation between Basidiomycota relative abundance and the plant dry-weight inhibition rate (*r* = −0.2892, *P* = 0.0462), while Ascomycota relative abundance showed a positive correlation (*r* = 0.4909, *P* = 0.0004) (Table 3). At the family level, Nectriaceae displayed the highest relative abundance (Fig. S5 A, C, E, G). Linear regression indicated that Tremellales_fam_Incertae_sedis (Basidiomycota) relative abundance negatively correlated with the plant dry-weight inhibition rate (*r* = −0.3804, *P* = 0.0076). In contrast, Nectriaceae, Hypocreaceae, and Helotiaceae (Ascomycota) showed positive correlations with inhibition rates of *r* = 0.4348 (*P* = 0.0020), *r* = 0.3372 (*P* = 0.0191), and *r* = 0.3330 (*P* = 0.0207), respectively (Table 3). These findings aligned with the results of the phylum-level linear regression analysis.

**Table 3:** Correlation coefficients (*r*) between the relative abundance of fungus and the rate of inhibition of plant dry weight in replanted orchards

Fungal	Correlation with plant growth (*r*)	OTUsize (%)^a^
Ascomycota	0.4909	0.66
Nectriaceae	0.4348	0.19
Chaetomiaceae	0.2459	0.06
Hypocreaceae	0.3372	0.01
Helotiaceae	0.3330	0.01
Basidiomycota	−0.2892	0.16
Zygomycota	−0.1644	0.10
Mortierellaceae	−0.1701	0.10
Lasiosphaeriaceae	−0.3465	0.03
Helotiales_fam_Incertae_sedis	−0.2370	0.01
Tremellales_fam_Incertae_sedis	−0.3804	0.02

When fungi were present in the soil of all ABG regions, and when the relationship with plant growth was > 0.15 or < − 0.15, they were all included in the table

^a^The average value of the sum of the abundances (as percentages) of this species in each sample

**Fig. 1:**
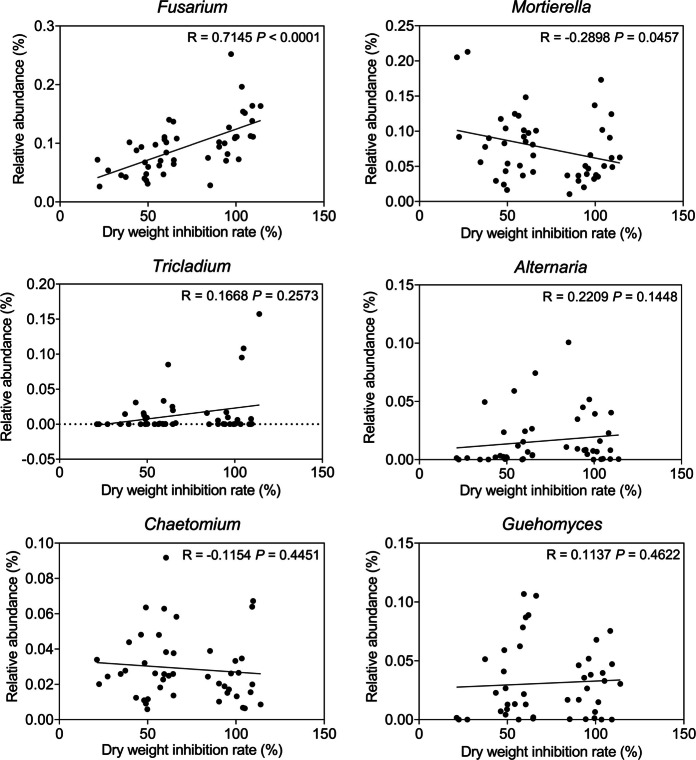
Relationship between the rate of inhibition of plant dry weight and the relative abundance of fungal genera. The regression lines indicate statistical significance at *P* < 0.05

At the genus level, *Fusarium* was the dominant genus in both NL and ABG regions (Fig. S5 B, D, F, H). Notably, *Alternaria* was more abundant than *Fusarium* in the PCT and QCT orchards. Krona analysis (Fig. S6) showed that Ascomycota, Sordariomycetes, Hypocreales, and Nectriaceae dominated the rhizospheric soil fungi, with varying *Fusarium* percentages. *Fusarium* accounted for 84% in the H orchard and 61% in the XQ orchard. Linear regression showed that the relative abundance of *Fusarium* positively correlated with the plant dry-weight inhibition rate (*R* = 0.7145, *P* < 0.0001). Additionally, the relative abundances of *Alternaria*, *Tricladium*, and *Guehomyces* increased with the inhibition rate across all samples (Fig. 1).

As is well known, ARD occurrence is closely associated with the deterioration of soil fungal communities, driven by soil physicochemical properties and phenolic acid content. Linear regression analysis revealed negative correlations between AK (*R*^*2*^ = 0.1643, *P* = 0.043) and ρb (*R*^*2*^ = 0.1116, *P* = 0.0203) with the plant dry-weight inhibition rate. SOM (*R*^*2*^ = 0.1512, *P* = 0.0063) and AN (*R*^*2*^ = 0.1332, *P* = 0.0107) showed positive correlations with the inhibition rate. AP, ω, and pH showed no significant correlation with the plant dry-weight inhibition rate (*P* > 0.05) (Fig. S7). Pd (*R*^*2*^ = 0.6001, *P* < 0.0001), Ca (*R*^*2*^ = 0.2276, *P* = 0.0006), syringate (St) (*R*^*2*^ = 0.1479, *P* = 0.070), and Pha (*R*^*2*^ = 0.1225, *P* = 0.0148) positively correlated with the inhibition rate. Ba and ferulic acid (Fa) decreased with increasing inhibition rates, while catechin (Ch) showed no clear relationship with the inhibition rate (Fig. S8).

### Compositional differences in rhizospheric fungi between DRS and HRS of the ABG region

Although the fungal taxa composition in DRS and HRS of the ABG region was similar, hierarchical clustering revealed distinct fungal communities (Fig. S9 and S10). In HRS, the relative abundance of Mortierellaceae (8.31%) was 1.34 times higher than in DRS. The relative abundances of Helotiaceae, Hypocreaceae, and Microascaceae in DRS were 2.28, 3.68, and 2.94 times higher, respectively, than in HRS. In contrast, Tremellales_fam_Incertae_sedis abundance in HRS was 1.35 times higher than in DRS. Linear regression indicated that the relative abundances of Tremellales_fam_Incertae_sedis and Mortierellaceae decreased with increasing plant dry-weight inhibition, whereas Helotiaceae and Hypocreaceae increased (Table 3).

At the genus level, the relative abundances of *Fusarium, Alternaria*, *Tricladium*, and *Guehomyces* in DRS were 1.21, 1.02, 2.31, and 1.05 times higher, respectively, than in HRS. In HRS, *Mortierella*, *Chaetomium*, and *Acremonium* were 1.41, 1.82, and 2.39 times richer than in DRS, with relative abundances of 7.93%, 4.31%, and 0.96% respectively. *Mortierella* was most abundant in QCJ, LCJ, PCJ, YLJ, JCJ, and DLJ orchards, while *Chaetomium* dominated in the HJ orchard (Fig. S11). Linear regression showed that the relative abundance of *Mortierella* negatively correlated with plant dry-weight inhibition (*R* = 0.2898, *P* = 0.0457). The relative abundance of *Chaetomium* decreased with increasing inhibition, while *Fusarium, Alternaria*, *Tricladium*, and *Guehomyces* increased (Fig. 1).

### Identification of differentially distributed fungal taxa

**Fig. 2:**
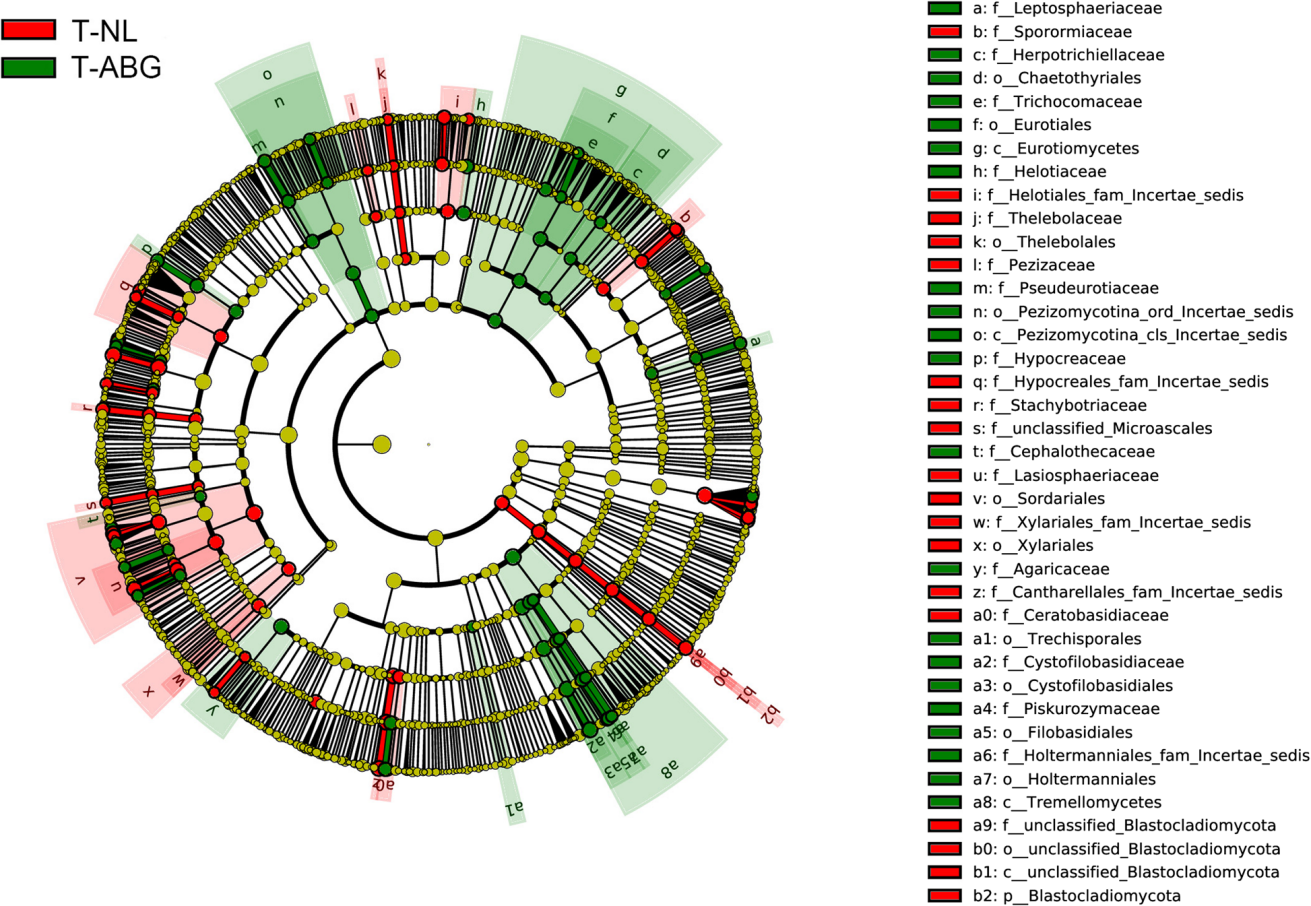
Taxonomic cladogram generated from the LEfSe analysis. The phylum, class, order, family, and genus levels are listed from the inside to the outside of the cladogram. Labels for family and genus are abbreviated using a single letter. Red and green colors indicate taxa enriched in NL and ABG replanted orchards, respectively, while yellow circles represent taxa without significant differences among the three cropping systems. The rhizosphere soil samples from trees with ARD symptoms in the ABG region are designated as T-ABG, while those from the NL region are referred to as T-NL

Rhizosphere fungal communities (RFC) from six NL and ten ABG regions were clustered hierarchically using the unweighted UniFrac algorithm. The RFC split into two main branches, with replanted orchards from NL regions forming one cluster and those from ABG regions forming a distinct cluster. NMDS results further confirmed the distinct separation between NL and ABG samples, reflecting significant differences in RFC (Fig. S12). A total of 18 and 21 fungal taxa were identified in the ABG and NL regions, respectively (Fig. 2). The ABG region showed enrichment of Cystofilobasidiaceae, Hypocreaceae, and Helotiaceae, while the NL region was enriched with Cantharellales_fam_Incertae_sedis, Helotiales_fam_Incertae_sedis, and Lasiosphaeriaceae (Fig. S13). These findings were consistent with the community composition analysis (Fig. S14 A). Metastats analysis revealed that *Tetracladium* had high relative abundances in the NL region, whereas *Guehomyces* and *Tricladium* were more abundant in the ABG region (0.0001 < *p* < 0.001) (Fig. S14 B).

**Fig. 3:**
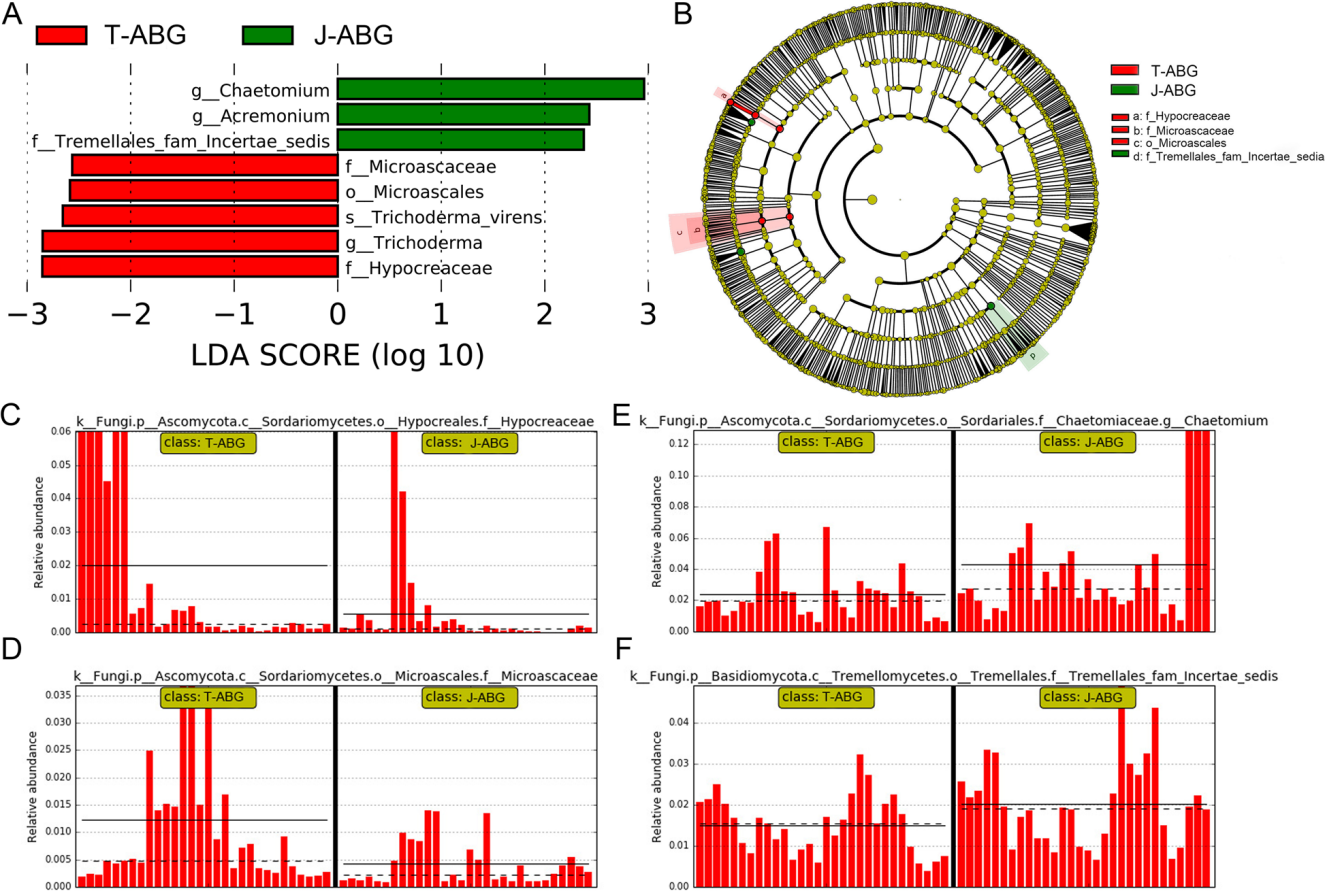
LDA score chart (**A**), taxonomic cladogram (**B**), and bar chart (**C**-**F**) generated from the LEfSe analysis. **A** LDA scores obtained from the Linear Discriminant Analysis (LDA) of microbial groups showing significant differences between the two groups. **B** The phylum, class, order, family, and genus levels are listed from the inside to the outside of the cladogram. Labels for family and genus levels are abbreviated using a single letter. Red and green indicate taxa enriched in diseased and healthy rhizospheric soil of apple trees in the ABG, respectively, while yellow circles represent taxa without significant differences among the three cropping systems. **C**-**F** Differential species information is displayed, with the ordinate representing relative abundance and the abscissa indicating sample names (each column corresponds to a sample; only group names are labeled in the figure, not individual sample names). Different groups are highlighted in yellow boxes. The rhizosphere soil samples from trees with ARD symptoms in the ABG region are designated as T-ABG, while those from healthy fruit trees are labeled J-ABG

A difference matrix heat map and NMDS were generated for DRS and HRS fungal communities using the unweighted UniFrac algorithm. DRS and HRS formed distinct clusters, indicating significant differences in their fungal communities (Fig. S15). LEfSe analysis identified 8 fungal taxa with significantly different relative abundances in DRS and HRS, comprising 1 order, 3 families, 3 genera, and 1 species (Fig. 3). HRS was enriched with *Chaetomium* (highest LDA score) and *Acremonium*, while DRS was enriched with Hypocreaceae (highest LDA score) and Microascaceae. HRS also showed enrichment of Tremellales_fam_Incertae_sedis, aligning with the community composition analysis (Fig. S14 C-D).

### Relationship between fungal taxa and environmental factors

**Fig. 4:**
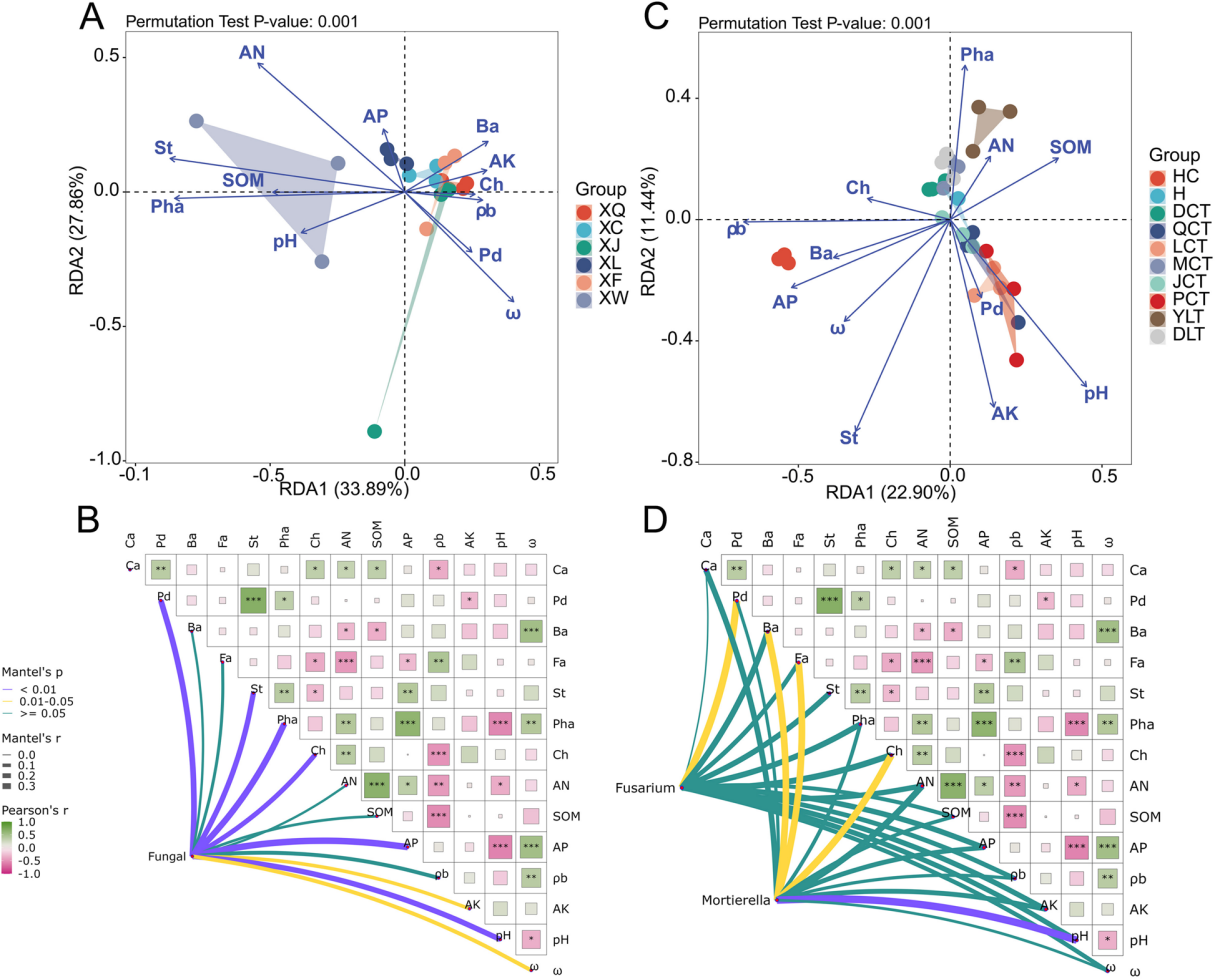
Relationship between the composition of fungal communities and major environmental factors: (**A**) NL and (**C**) ABG. Environmental factors are indicated by blue arrows, and different orchards are represented by different shapes. Spearman analysis of the relationships between soil physicochemical properties and the most abundant fungal genera in the rhizospheric soil of replanted orchards (**B**, **D**). Soil properties include available phosphorus (AP), available potassium (AK), organic matter (SOM), soil bulk density (ρb), available nitrogen (AN), pH, soil moisture content (ω), phloridin (Pd), benzoic acid (Ba), syringate (St), cinnamic acid (Ca), ferulic acid (Fa), p-hydroxybenzoic acid (Pha), and catechin (Ch). The R values are represented by different colors in the figure (green indicates positive correlation, violet indicates negative correlation; the color intensity and grid size reflect the strength of the correlation). The right legend shows the color range for different R values. Significance levels are indicated as follows: * 0.01 < *p* ≤ 0.05, ** 0.001 < *p* ≤ 0.01, *** *p* ≤ 0.001. The thickness and color of the lines connecting nodes and environmental factors indicate their degree of correlation: purple (*p* < 0.01), yellow (0.01 ≤ *p* < 0.05), green (*p* ≥ 0.05)

The relationship between fungal taxa at the genus level and major environmental factors was analyzed (Fig. 4 A, C). In the NL region, AN, ω, St and Pha significantly influenced the composition of the main fungal populations in the rhizosphere (*p* < 0.05). In the ABG region, AP, ρb, AK, pH, ω, St and Pha significantly affected the composition of the main fungal populations in the rhizosphere (*p* < 0.05). Spearman analysis revealed significant correlations between phenolic acids (Ba, Ca, Fa, Pha, Ch) and major soil properties (Fig. 4 B, D). The relative abundance of main fungal genera in the rhizospheric soil of replanted orchards showed significant correlations with Pd, St, Pha, Ch, AP, and pH (*p* < 0.01), weakly correlated with AK and ω (0.01 ≤ *p* < 0.05), and no significant correlations with Ba, Fa, AN, and SOM (*p* ≥ 0.05). Notably, the relative abundance of *Fusarium* significantly correlated with Pd (0.01 ≤ *p* < 0.05), while the relative abundance of *Mortierella* showed significant correlations with Ba, Fa, Ch, and pH, particularly with pH (*p* < 0.01).

Redundancy analysis indicated that, in the NL region, *Fusarium* significantly correlated with Pd and AN. pH, AP, and SOM positively influenced *Gibberella* and *Verticillium*. ρb, AK, and ω positively affected *Mortierella*. *Chaetomium* showed a positive correlation with AN. The influence of major environmental factors on the main fungal genera followed the order: AN > pH > SOM > ρb > ω > AP > Pd > AK. In the ABG region, *Fusarium* and *Gibberella* significantly correlated with AN and SOM, suggesting that AN and SOM levels reflect their relative abundances. pH positively influenced *Verticillium* and *Mortierell*a. AP and ω positively affected *Guehomyces*. AK and Pd positively influenced *Mortierella* and *Guehomyces*. The influence of major environmental factors on the main fungal genera followed the order: SOM > pH > AP > ω > ρb > AN > Pd > AK (Fig. S16).

**Fig. 5:**
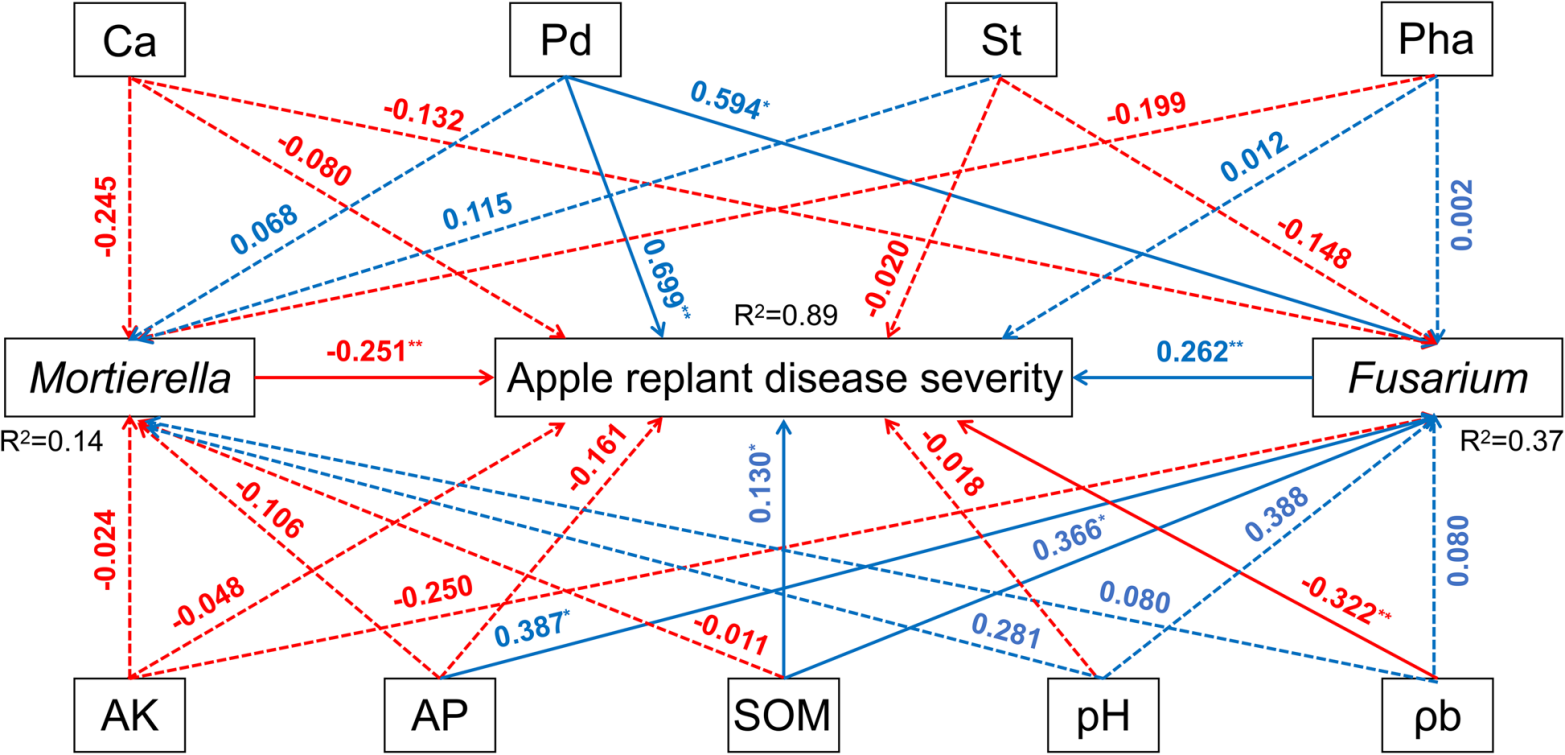
Structural equation model (SEM) showing the causal relationships among the content of soil phenolic acids (cinnamic acid, syringate, *p*-hydroxybenzoic acid, phloridin), the severity of apple replant disease, soil properties (ρb, AP, pH, AK, and SOM), and the relative abundance of *Mortierella* and *Fusarium*. Arrows indicate significant relationships, dotted lines indicate non-significant relationships, and solid lines indicate significant effects. Blue lines represent positive effects, and red lines represent negative effects. *R²* indicates the proportion of variance explained by the model. Model fit summary (χ² = 9.380, NFI = 0.972, SRMR = 0.024) is provided. Numbers above arrows indicate path coefficients. Significance levels: *p* < 0.05, * *p* < 0.01)

We analyzed the relationship between soil properties, phenolic acids, ARD severity, and the relative abundances of *Mortierella* and *Fusarium*. SEM (χ^2^ = 148.090, NFI = 0.562, SRMR = 0.160) (Fig. S17A) explained 82.0% of the variations in the ARD severity, 16.0% of the variations in the *Mortierella*, and 28.0% of the variations in the *Fusarium*. SEM (χ^2^ = 110.708, NFI = 0.673, SRMR = 0.126) (Fig. S17B) explained 82.0% of the variations in the ARD severity, 15.0% of the variations in the *Mortierella*, and 28.0% of the variations in the *Fusarium*. Therefore, we focused on soil properties (ρb, AP, pH, AK, and SOM) and soil phenolic acids (Ca, Pha, Pd, and St) to analyze the overall relationship. The best-fitting SEM (χ^2^ = 9.380, NFI = 0.972, SRMR = 0.024) explained 89.0% of the variations in the ARD severity, 14.0% of the variations in the *Mortierella*, and 37.0% of the variations in the *Fusarium* (Fig. 5). SOM and AP were significantly positively correlated with the relative abundance of *Fusarium*, and non-significantly decreased the relative abundance of *Mortierella.* SOM significantly influenced ARD severity. Pd in the rhizosphere soil was significantly positively correlated with the relative abundance of *Fusarium*, thereby increasing ARD severity. *Mortierella* in the rhizosphere soil was significantly negatively correlated with the ARD severity.

### Functional groups of fungi

**Fig. 6:**
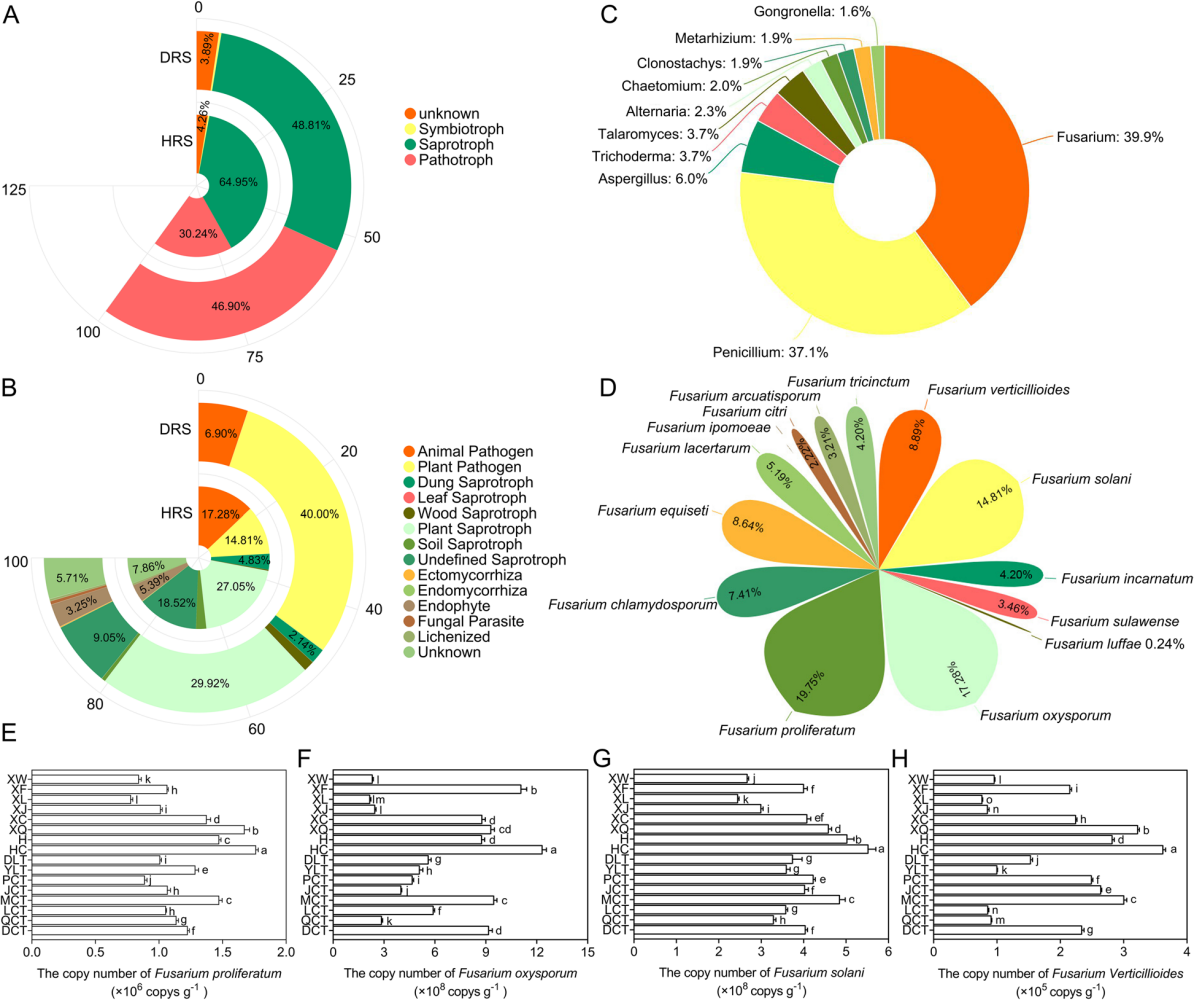
**A** Trophic composition of rhizospheric fungal communities. **B** Functional composition of rhizospheric fungi. T: Rhizospheric soil samples from diseased apple trees; J: Rhizospheric soil samples from healthy apple trees. Results with relative abundance less than 0.1% are not shown. **C**-**D** Percentage of the top 10 fungi and *Fusarium* species isolated from the rhizosphere soil of diseased apple trees. **E**–**H** Gene copy numbers of *F. verticillioides*, *F. oxysporum*, *F. proliferatum*, and *F. solani* in replanted orchards. Numbers followed by the same letter are not significantly different based on Duncan’s Multiple Range Test at α = 5%

Analysis using the FUNGuild database revealed that most fungi in HRS and DRS were saprotrophs, followed by pathotrophs, with symbiotrophs accounting for only 0.40% and 0.56%, respectively (Fig. 6A). A key finding was the significantly higher percentage of pathotrophs in DRS compared to HRS. Additionally, HRS exhibited a higher proportion of saprophytes. Fungal isolates were classified into 14 functional types based on their soil resource preferences (Fig. 6B), including animal pathogens, plant pathogens, dung saprotrophs, leaf saprotrophs, wood saprotrophs, plant saprotrophs, soil saprotrophs, undefined saprotrophs, ectomycorrhizals, endomycorrhizals, endophytes, fungal parasites, lichenized fungi, and unknown (Shen et al. 2020). The functional groups showed significant differences in DRS and HRS. Plant pathogens dominated in DRS, whereas plant saprophytes were most abundant in HRS, followed by undefined saprophytes.

*Fusarium*, *Penicillium*, *Trichoderma*, *Talaromyces*, and *Aspergillus* were the dominant fungal genera in replanted orchard soils, representing 32.98%, 30.70%, 3.09%, 3.09%, and 4.97% of the total isolates, respectively (Fig. 6C). Morphological and molecular identification detected 14 *Fusarium* species, with *F. verticillioides* (8.89%), *F. oxysporum* (17.28%), *F. proliferatum* (19.75%), and *F. solani* (14.81%) being the most prevalent (Fig. 6D). qPCR analysis (Fig. 6E-H) detected DNA from *F. verticillioides*, *F. oxysporum*, *F. proliferatum*, and *F. solani* in all rhizospheric soil samples of diseased apple trees, with varying concentrations. The abundance of *F. verticillioides*, *F. oxysporum*, *F. proliferatum*, and *F. solani* was notably higher in HC, H, XQ, and MCT orchards. This finding aligns with Krona analysis results, which also showed higher *Fusarium* OTUs abundance in HC, H, XQ, and MCT orchards.

### Pathogenicity test

**Fig. 7:**
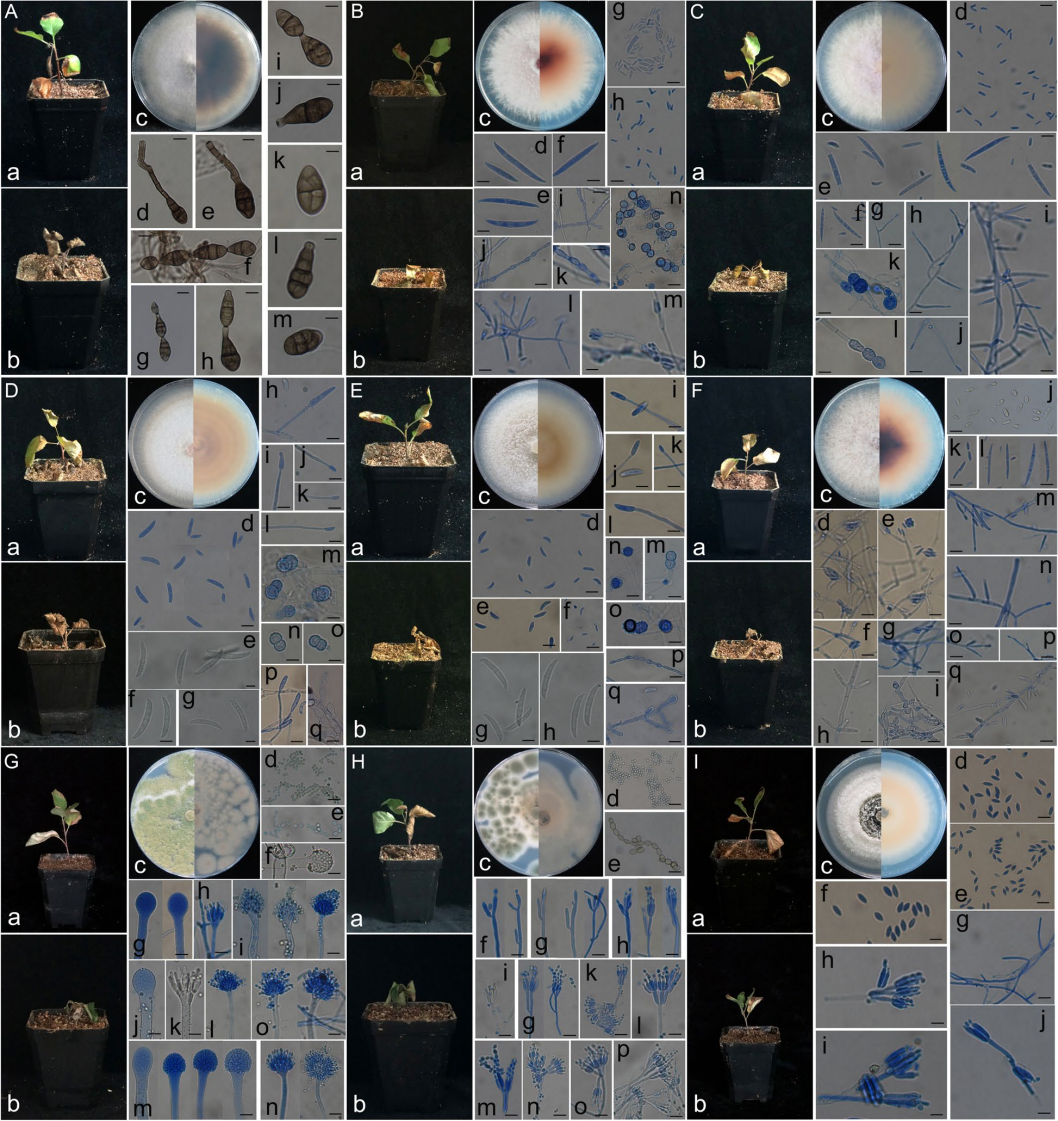
Disease symptoms of M.9T337 and *Malus hupehensis* Rehd. seedlings, along with culture characteristics and microscopic appearance of isolates. **a**-**b** Disease symptoms in M.9T337 (**a**) and *Malus hupehensis* Rehd. seedling (**b**). **c** Fungal culture on potato-dextrose agar medium. **A** *Alternaria alternata* YR9 (isolated from root, Yiyuan, China). **d**-**m**: Conidia and conidiophores on potato-carrot agar medium. Scale bar: **d**, f = 20 μm; **e**, h = 15 μm; i-l = 10 μm. **B** *F. oxysporum *YR15 (isolated from root, Penglai, China). **d**-**f**: Macroconidia. **g**-**h**: Microconidia. **i**-**m**: Microconidial false heads from monophialides. **n**: Chlamydospores. Scale bars = 10 μm. **C** *F. oxysporum* HC131(isolated from soil, Daliang, China). **d**: Microconidia. **e**-**f**: Macroconidia. **g**-**j**: Microconidial false heads from monophialides. **k**-**l**: Chlamydospores. Scale bars = 10 μm. **D** *F. solani* HC39 (isolated from soil, Penglai, China). **d**: Microconidia. **e**-**g**: Macroconidia. **h**-**l**, **p**-**q**: Monophialides. **m**-**o**: Chlamydospores. Scale bars: d = 5 μm; e-q = 10 μm. **E** *F. solani *Q61 (isolated from soil, Qixia, China). **d**-**f**: Microconidia, **g**-**h**: Macroconidia, **i**-**l**, **p**-**q**: Monophialides, **m**-**o**: Chlamydospores. Scale bars: **f**, k = 20 μm; d-e, g-j, l-o = 10 μm. **F** *F. proliferatum* MR5 (isolated from root, Muping, China). **d**-**i**, **m**-**q**: Microconidial false heads from monophialides and polyphialides. **j**-**k**: Microconidia. **l**: Macroconidia. Scale bars = 15 μm. **G**: *Aspergillus flavus* XW23 (isolated from soil, Shanxi, China). **d**-**e**: Conidia. **f**-**g**, **j**, **m**: Conidiophores and apical vesicles. **h**-**i**, **k**-**l**, **n**-**o**: Bottle stems. Scale bars: **d**, **f**-**g**, **j**-**k**, m = 20 μm; e = 15 μm; **h**-**i**, **l**, n-o = 30 μm. **H** *Penicillium brasilianum* Q9 (isolated from soil, Qixia, China). **c**: Culture on Czapek yeast agar (CYA) medium. **d**-**e**: Conidia. **f**-**p**: Conidiophores. Scale bars: d = 20 μm; e = 10 μm; f-p = 15 μm. I: *Albifimbria verrucaria* XW39 (isolated from soil, Shanxi, China). **d**-**f**: Conidia. **g**: Hyphae. **h**-**j**: Conidiogenous cells. Scale bars: **d**, f-j = 10 μm; e = 15 μm

Greenhouse pathogenicity tests revealed that the isolates listed in Table S18 were highly pathogenic to M.9T337 and *Malus hupehensis* Rehd. seedlings, causing wilting and death within 2 weeks. Morphological and phylogenetic identification confirmed the following isolates (Table S18; Fig. 7; Fig. S18-24): *Alternaria alternata* YR9, five *Fusarium* strains (*F. oxysporum* YR15, HC131; *F. solani* HC39, Q61; *F. proliferatum* MR5), *Aspergillus flavus* XW23, *Penicillium brasilianum* Q9, *Albifimbria verrucaria* XW39, and *Phoma macrostoma* HC139. The disease intensity of *Fusarium* isolates varied between M.9T337 and *Malus hupehensis* Rehd. Specifically, 42.47% of *Fusarium* isolates caused disease intensity exceeding 30% in M.9T337, while 52.84% caused disease intensity exceeding 30% in *Malus hupehensis* Rehd. Notably, over 76% of *Fusarium* isolates exhibited disease intensity greater than 10% in both apple seedlings (Table S19). Control apple seedlings remained healthy, with green leaves showing no signs of disease (Fig. S18 A, B). Diseased seedlings exhibited browning starting from the leaf edges, with spots rapidly expanding and accompanied by chlorosis. Over time, entire leaves turned brown to dark brown, followed by rolling, yellowing, wilting, and eventual death (Fig. 7 A-I: a, b; Fig. S18 C, D).

When the *Fusarium* complex (five strains) was inoculated alone or in combination with other fungal isolates, disease intensity (DI) reached 100%, and seedlings died within two weeks (Table 4). This finding, along with reduced root infection frequency of *Alternaria alternata* YR9 during coinoculation, suggests weak competitiveness of this fungus against the *Fusarium* complex in root colonization. *Penicillium brasilianum* Q9 and *Aspergillus flavus* XW23, owing to their rapid reproduction, compete effectively with the *Fusarium* complex in soil and may synergistically contribute to seedling wilting and death. However, the precise mechanisms underlying their cooperative effects require further investigation.

**Table 4:** Pathogenicity and root infection frequency of *Aspergillus*, *Alternaria*, *Penicillium*, and *Fusarium* mixture (*F. oxysporum* YR15, HC131; *F. solani* HC39, Q61; *F. proliferatum* MR5) alone and when co-inoculated on apple seedlings

Treatment^a^	Disease intensity (DI)	Relative infection frequency^b^			
		PB (%)	AF (%)	FF (%)	AA (%)
*Aspergillus flavus* XW23 (AF)	100%		100		
FF + AF	100%		40.21	55.56	
*Fusarium* mixture (FF)	100%			100	
FF + PB	100%	40.72		51.50	
*Penicillium brasilianum* Q9 (PB)	100%	100			
FF + AA	100%			70.54	22.32
*Alternaria alternata* YR9 (AA)	100%				100
Control	0				

^a^Average above-ground dry weight produced by ten plants in each of three replicates (30 pots per treatment)

^b^Recovery of the inoculated fungi from seedling roots is relative to total root fungal colonization

### Extracellular enzymatic activity and fungal fungicide sensitivity analysis

*Fpmd* MR5 was found to produce pectinase and cellulase (Fig. S25 A-B). PGAse and CMCellulase activities increased gradually from 24 to 96 h, peaked at 96 h (633.05 U·mL^−1^) and 120 h (493.64 U·mL^−1^), respectively, before gradually declining (Fig. S25 C-D). Indoor inhibition tests revealed that all 11 tested fungicides inhibited the mycelial growth and spore germination of *Fpmd* MR5 (Tables 5 and 6, Fig. S26). Among the tested fungicides, flusilazole and bromothalonil exhibited significantly stronger inhibitory effects on mycelial growth and spore germination of *Fpmd* MR5, showing the highest indoor toxicity., Mancozeb and carbendazim followed in effectiveness. The EC_50_ values for mycelial growth and conidia germination were 2.47–3.59 mg·L^−1^ and 2.51–3.98 mg·L^−1^, respectively. Except for prochloraz, carbendazim, and mancozeb, the other fungicides showed stronger inhibition of conidia germination than of hyphal growth.

**Table 5:** Effects of 11 fungicides on mycelium growth of *Fusarium proliferatum* f.sp. *malus domestica*

Fungicide	EC_50_ (mg·L^−1^)	Correlation coefficient (*R*^*2*^)	Regression equation of toxicity	95% CL (mg·L^−1^)
Azoxystrobin	23.4	0.98	y = 0.65x-0.89	15.59 ~ 35.13
Prochloraz	5.88	0.96	y = 0.13x-0.1	3.55 ~ 9.73
Pyraclostrobin	56.23	0.97	y = 0.2x-0.35	34.52 ~ 91.61
Difenoconazole	12.05	0.96	y = 0.37x-0.4	7.44 ~ 19.53
Flusilazole	-	-	-	-
Bromothalonil	-	-	-	-
Thiophanate-Methyl	8.78	0.94	y = 0.53x-0.5	5.49 ~ 14.03
Zineb	10.41	0.99	y = 0.57x-0.58	6.59 ~ 16.46
Carbendazim	3.59	0.96	y = 0.18x-0.1	2.17 ~ 5.95
Mancozeb	2.47	0.71	y = 0.61x-0.24	1.52 ~ 4.02
Chlorothalonil	9.33	0.95	y = 0.33x-0.32	5.71 ~ 15.23

"-"means that the inhibition rate of mycelium treated with different concentrations of fungicides is 100%

**Table 6:** Efects of 11 fungicides on conida germination of *Fusarium proliferatum* f.sp. *malus domestica*

Fungicide	EC_50_ (mg·L^−1^)	Correlation coefficient (*R*^*2*^)	Regression equation of toxicity	95% CL (mg·L^−1^)
Azoxystrobin	12.28	0.96	y = 1.01x-1.1	11.33 ~ 13.3
Prochloraz	13.11	0.99	y = 0.17x-0.19	11.79 ~ 14.58
Pyraclostrobin	22.64	0.97	y = 0.31x-0.42	20.45 ~ 25.07
Difenoconazole	4.49	0.95	y = 0.23x-0.15	4.04 ~ 4.99
Flusilazole	-	-	-	-
Bromothalonil	-	-	-	-
Thiophanate-Methyl	3.98	0.96	y = 0.35x-0.21	3.58 ~ 4.42
Zineb	6.11	0.97	y = 0.14x-0.11	5.49 ~ 6.79
Carbendazim	3.98	0.94	y = 0.1x-0.06	3.58 ~ 4.43
Mancozeb	2.51	0.68	y = 0.15x-0.06	2.26 ~ 2.8
Chlorothalonil	4.53	0.96	y = 0.32x-0.21	4.08 ~ 5.04

"-"means that the rate of conidia germination treated with different concentrations of fungicides is 0%

## Discussion

Fungal ITS sequencing revealed that Ascomycota was the most abundant phylum in the ABG and NL regions, as well as in continuous cropping soils of strawberry and *Pisum sativum* (Franke-Whittle et al. 2015; Li et al. 2010; Li and Liu 2019). This may be attributed to poor nutrient substrates, as soil nutrients such as TP, AN, AP, and AK have been reported to negatively affect Ascomycota abundance (Li et al. 2020). Subsequent LEfSe and NMDS analyses highlighted significant differences in fungal taxa composition between the ABG and NL regions. The differences in fungal composition and relative abundance between ABG and NL regions are likely driven by factors such as soil nutrients, plant species, agricultural management practices, soil type, and phenolic acid accumulation (Li et al. 2010; Wang et al. 2020).

The diversity and composition of rhizosphere fungal community are closely related to healthy plant growth and disease development (Větrovský et al. 2020). In this study, differences in the composition and diversity of fungal communities between DRS and HRS were observed, suggesting a close association with ARD occurrence. The relative abundances of *Fusarium, Alternaria*, *Tricladium*, and *Guehomyces* were significantly higher in DRS than in HRS, Most of these genera have been identified as dominant in continuous cropping soils (Jiang et al. 2017; Li et al. 2020; Li and Liu 2019; Liao et al. 2021; Nagrale et al. 2016; Song et al. 2018; Zhu et al. 2018; Wu et al. 2018, 2019). The *Gibberella*/*Fusarium* group (*Gibberella* being the sexual stage of *Fusarium*) is well-known for causing root rot and fusarium wilt in plants (Amatulli et al. 2010; Gálvez et al. 2017; Moretti 2009). Thus, increased *Fusarium* abundance likely contributes to ARD. Additionally, Franke-Whittle et al. (2015) found that *Fusarium* and *Gibberella* negatively correlated with plant growth, further underscoring their pathogenic role in replanted orchards. According to the literature, *Tricladium* species grow as saprotrophs on dead leaves or wood and are also known to cause leaf spot disease as parasites (Gupta and Gandhi 1979). Thus, the occurrence of ARD may be closely linked to the increase abundance of pathogenic fungi in rhizospheric soil.

We also observed higher relative abundances of *Mortierella*, *Chaetomium*, and *Acremonium* in HRS. LEfSe analysis further showed that *Chaetomium* and *Acremonium* played important roles in HRS, while Hypocreaceae and Microascaceae were prominent in DRS. According to the literature, *Mortierella* and *Chaetomium* enhance plant nutrient utilization and are linked to reduced disease incidence (Shen et al. 2020; Yao et al. 2019). *Acremonium* produces bioactive secondary metabolites that enhance plant disease resistance (Tian et al. 2017), whereas Microascaceae, a dominant family in continuous cropping soils, negatively correlates with shoot dry weight and stem diameter (Jiang et al. 2017). Notably, Franke-Whittle et al. (2015) reported a strong negative correlation between *Acremonium* (*r* < −0.81) and plant growth, and this genus was also detected in peach replant disease-affected soils (Yang et al. 2012). However, its low relative abundance (0.96%) suggests it is not a major contributor, and further research is needed to clarify its role in ARD.

The investigation of soil fungi in diseased (rhizospheric) soil offers a promising approach for identifying biological control agents to control ARD (Gu et al. 2020). In this study, *Aspergillus*, *Penicillium*, and *Fusarium* were the most frequently isolated fungi from rhizospheric soil. Among these, *Fusarium* was the dominant genus, further corroborating the high-throughput sequencing results and aligning with the findings of Kocić-Tanackov et al. (2017). Additionally, DNA from *F. verticillioides*, *F. oxysporum*, *F. proliferatum*, and *F. solani* was detected in all DRS from the ABG and NL regions. Similarly, Chen et al. (2020) reported significantly increased abundances of *F. solani* and *F. oxysporum* in long-term monocropping systems. According to the literature, *Fusarium* species (*F. oxysporum*, *F. proliferatum*, and *F. solani*) are well-documented pathogens causing root rot, vascular wilting, yellowing, and foliar necrosis (Fei et al. 2021; Gálvez et al. 2017). These findings were further supported by the pathogenicity tests conducted in this study. *F. oxysporum*, *F. proliferatum*, and *F. solani* were highly pathogenic, causing apple seedlings to wither and die, suggesting their potential role as ARD pathogens in China. Similarly, the relative abundance of *Fusarium* increased significantly in continuous cropping systems of Chinese chives, cucumber, and watermelon, leading to plant damage (Wu et al. 2019; Wang et al. 2019; Zhou and Wu 2012). However, some studies have shown that certain *Fusarium* species (e.g., *F.oxysporum*, *Fusarium tricinctum*, and *Fusarium chlamydosporum*) can promote plant growth (Fravel et al. 2003; Boari and Vurro 2004; Zhang et al. 2014). A similar trend was observed in this study, where some *Fusarium* species exhibited no pathogenic effects. Additionally, extensive evidence suggests that ARD is a complex disease with multiple components, some of which may vary across different orchard sites (Ali et al. 2024). In a transnational European study, *pythium* species were identified as pathogenic agents of ARD in soils from Austria, Germany, and Italy (Manici et al. 2003). *Pythium*, *Rhizoctonia*, and *Phytophthora* have frequently been identified as key pathogenic components of ARD in Washington (Mazzola 1998; Mazzola et al. 2002). Currently, many biosystematists classify flagellate fungi, including oomycetes, within the Kingdom Protista rather than Fungi (Hibberd and Leedale 1971; Lipscomb 1982; Ligrone 2022; Margulis 1974; Yu 1986). The absence of oomycetes in this study may be attributed to the use of primers with limited target specificity and/or lower PCR efficiency, which are known to fail in amplifying the ITS1 region of many fungi (Op De Beeck et al. 2014). In future studies, we plan to use oomycete-specific primers for sequencing analysis, with a focus on optimizing this process.

We also identified *Alternaria alternata*, *Aspergillus flavus*, *Penicillium brasilianum*, *Albifimbria verrucaria*, and *Phoma macrostoma* as highly pathogenic to apple seedlings. According to the literature, *Alternaria* species are known to produce toxins that cause a range of diseases, including black spotting, stem wilting, and brown spotting (Nagrale et al. 2016). *Aspergillus flavus*, a major source of mycotoxin contamination in crops, is also associated with fruit rot in table grapes (Ghuffar et al. 2020). *Penicillium brasilianum* is the primary pathogen responsible for rot in onion bulbs (Sang et al. 2014). *Albifimbria verrucaria*, which has a broad host range, has been reported to cause stem necrosis and leaf spotting in tomato in Italy (Matić et al. 2019). *Phoma* exhibits a higher colonization rate on diseased plants in continuous cropping systems and is also known to cause root crown disease in weeds (Caesar et al. 2012). This discovery offers new insights for the biological control of ARD and holds significant practical implications for future agricultural practices.

NMDS analysis revealed distinct clustering of DRS and HRS samples, indicating significant differences in their fungal communities. We also observed higher fungal community richness (Sobs, ACE, and Chao) and Simpson index in DRS compared to HRS. Similarly, numerous studies have reported that continuous cropping significantly increases soil fungal community richness (Li and Liu 2019; Liao et al. 2021; Wang et al. 2020). However, it had no significant effect on the diversity of fungal communities (Gu et al. 2020; Li et al. 2010). Consistent with these findings, our study found no significant difference in Shannon diversity between DRS and HRS, likely due to the influence of soil environment, plant species, and phenolic acids on fungal communities (Wang et al. 2012, 2017). Thus, the occurrence of ARD appears to be closely linked to the composition and abundance of fungal taxa.

In recent years, the influence of dominant soil fungal taxa and environmental factors such as SOM, AN, AP, pH, and AK has garnered increasing attention from researchers (Jiang et al. 2017; Fei et al. 2021). Previous studies have found that pH significantly influences the composition of fungal taxa in continuous cropping soils (Li et al. 2020; Song et al. 2018; Zhang et al. 2019). This study further confirmed these findings, revealing a statistically significant relationship between pH and the relative abundance of fungi in the rhizospheric soil of replanted orchards, particularly for *Mortierella* and *Fusarium*. We also observed that key soil properties, including AP, AK, and SOM, significantly influenced the abundance of dominant fungal genera such as *Fusarium* and *Gibberella*. Similarly, numerous studies have reported that AP positively influences the abundance of *Fusarium* and *Gibberella*, highlighting the close relationship between soil nutrients and fungal taxa composition (Li and Liu 2019; Větrovský et al. 2020; Xiang et al. 2021a; Wu et al. 2018; Wang et al. 2020). In this study, AN and ρb were also significantly correlated with the severity of apple replant disease. These results suggest that optimizing the application of phosphate, potassium, and nitrogen fertilizer, as well as organic matter, can help to mitigate ARD (Ju et al. 2007).

Previous studies have shown that the accumulation of phenolic acids in the rhizosphere is a major factor contributing to ARD, as it stimulates the reproduction of pathogenic microorganisms and exacerbates the disease. Similarly, after pathogen infection, phenolic compounds such as phloridin accumulate and are oxidized by polyphenol oxidase (PPO) to form quinones. These quinones undergo non-enzymatic polymerization, producing brown tannin-like substances (melanin), which ultimately leading to cell necrosis and root browning (Siefen et al. 2024; Wang et al. 2012). In this study, we observed a similar phenomenon. Phenolic acids (Pd, St, Ca, and Pha) were strongly associated with ARD development and significantly influenced the composition of fungal taxa (Bai et al. 2019; Xiang et al. 2021b; Yin et al. 2016, 2017). These phenolic acids showed positive correlations with fungal pathogens, including *Fusarium*, *Gibberella, Alternaria*, *Paramyrothecium*, and *Verticillium* (Guo et al. 2011; Gramaje et al. 2018; Matić et al. 2019). Similarly, Li et al. (2014) found that the gradual addition of peanut root exudates stimulated the growth of *F. oxysporum* while reducing the abundance of *Mortierella*. *Fusarium* can utilize Pd as a carbon source to enhance its growth and conidiospore production, while simultaneously inhibiting the root vigor of apple seedlings. Consistent with these findings, our study revealed a significant positive correlation between Pd levels and the relative abundance of *Fusarium*, both of which were associated with the severity of ARD. These results suggest that the accumulation of ARD-related phenolic acids exacerbates the disease by altering the composition of fungal taxa in the rhizosphere. Therefore, introducing antagonistic microorganisms capable of degrading Pd could be a promising strategy for controlling ARD.

The isolated fungi were classified into ecological groups (symbiotrophs, saprotrophs, and pathotrophs) according to FUNGuild (Liu et al. 2019; Nguyen et al. 2016).This study revealed that DRS had a higher proportion of pathotrophs compared to HRS, indicating that the enrichment of pathogenic fungi may contribute to the occurrence of ARD. Similarly, Li et al. (2020) reported a higher proportion of pathotrophs in soils subjected to continuous cropping for over 10 years. Notably, the fungi with higher isolation frequency in DRS were predominantly plant pathogens, including *Fusarium*, *Aspergillus*, and *Alternaria*. These fungal taxa, which showed higher relative abundance in DRS across both regions, have been linked to various plant diseases (Guo et al. 2011; Gramaje et al. 2018; Matić et al. 2019). These findings indicate that continuous cropping can lead to the accumulation of pathogenic fungi in the rhizosphere, potentially exacerbating plant disease. Liu et al. (2019) classified *Mortierella* as an undefined saprophytic fungus using FUNGuild, consistent with fungal ITS sequencing results showing that *Mortierella* in HRS was the main fungal genus distinguishing it from DRS. Additionally, the proportion of saprophytes (including plant, undefined, and wood saprotrophs) was higher in the fungal isolates from HRS. The results of this study demonstrate distinct composition ratio of ecological groups between DRS and HRS, indicating that ARD may significantly alter fungal functional profiles.

Plant cell wall degrading enzymes (CWDEs) mainly include pectinase, cellulase, hemicellulase, protease, amylase, and phospholipase. These enzymes degrade the cell walls of host plants, facilitating the invasion and colonization of pathogens (Binyamin et al. 2019). Among them, pectinase and cellulase play a particularly important role in the pathogenic process of pathogenic fungi (Laluk and Mengiste 2010; Lionetti et al. 2012). The ability of pathogenic fungi to produce CWDEs is a key indicator of their pathogenicity. For example, the virulence of *Fusarium compactum* in broomrape infection is enhanced by cellulolytic and pectinolytic enzymes, which facilitate host penetration (Babalola et al. 2010). We found that *Fpmd* MR5 produces both pectinase and cellulase, with pectinase activity being significantly higher than cellulase activity. Pectinase activity peaked at 96 h, while cellulase activity peaked at 120 h. Although the activity of both enzymes gradually declined after their respective peaks, cellulase activity remained lower than that of pectinase. This observation aligns with the established understanding of fungal pathogen invasion mechanisms in host plants. Initially, pectolytic enzymes diffuse extracellularly, loosening the middle lamella matrices and host cell walls to facilitate pathogen ingress. Subsequently, cellulose-degrading enzymes promote the softening and disintegration of the host cell wall, enabling fungal penetration into host cells and the development of disease (Benhamou and Côté 1992; Brito et al. 2006; Wilson 2009).

The two most effective indoor control agents in this study, flusilazole and bromothalonil, were screened by measuring the inhibitory effects of 11 fungicides on the mycelial growth and conidia germination of *Fpmd* MR5. Flusilazole is a broad-spectrum, high efficient, and systemic triazole fungicide belonging to the subclass of ergosterol biosynthesis inhibitors. It is commonly used to control fungal disease caused by ascomycete and basidiomycete pathogens (Ozakca and Silah 2013), and has been registered in China for control of a wide range of plant diseases (Lu et al. 2015; China Pesticide Information Network, http://www.chinapesticide.gov.cn). Yu et al. (2011) found that flusilazole residues in apples at harvest were below 0.05 mg·kg^−1^ at both the recommended high dose and 1.5 times that dose. Similarly, Amin et al. (2024) evaluated the potential risks of flusilazole to human health, particularly its effects on the endocrine, reproductive, and immune systems. Bromothalonil [2-bromo-2-(bromomethyl)pentane dinitrile], also known as methyldibromo glutaronitrile, is an easily degraded, highly active, low toxic fungicide with broad spectrum of disease control. It was registered and promoted for use in China in 2008 and is widely applied to fruits and vegetables to control anthracnose disease (Liang et al. 2020). Liu et al. (2014) found that in apples, the final residue amount of bromothalonil was lower than China's maximum residue limit (0.2 mg·kg^−1^) 7 d after application. The above research results provide a scientific basis for the safe use of flusilazole and bromothalonil in apple cultivation, ensuring consumer health protection and food safety supervision. In conclusion, flusilazole and bromothalonil can be selected to control ARD in the future.

## Conclusions

Taken together, our study shows that the composition, relative abundance, and function of the fungal taxa in HRS and DRS differed significantly, and the soil properties (pH, SOM, AP, ρb, and AK) and phenolic acid (Pd, St, Ca, and Pha) may significantly affect the composition of fungal groups in the rhizosphere soil. Further analysis showed that the deterioration of microbial structures in rhizospheric soil with more pathogenic fungi (mainly *F. proliferatum*, *F. oxysporum*, and *F. solani*) may have contributed to the severe growth reduction associated with replant disease-like symptoms. Therefore, in response to the national policy of green agricultural environmental protection, targeted treatment of specific microbial communities may become an important pathway for controlling ARD. In addition, given the complexity and regional differences in the causes of ARD, combined use of alternative management strategies, including soil amendment, biofumigation, inoculation with beneficial endophytes, and the deployment of different rootstock genotypes, may be more effective than a single strategy.

## Materials and methods

### Sample preparation

In July 2019, samples (root and rhizospheric soil) were collected from sixteen orchards, including old orchards (more than 20 years old) and newly replanted orchards (replanted 3–5 years ago). Ten of the orchards were located in the ABG region (MC, PC, LC, QC, YL, DL, DC, JC, HC, and H) and six were in the NL region (XQ, XC, XJ, XF, XL, and XW) (Table S1). These orchards were selected due to the presence of plants showing typical symptoms of replant disease in small localized plots, while the remaining trees in the orchards exhibited optimal vigor (Fig. S1). Dead/weak and well-growing apple trees were selected from each orchard in the two main apple planting regions, ABG and NL. The plant height and ground diameter of ARD-symptomatic apple trees were significantly lower than those of well-growing apple trees (Table 7). Within each orchard, three sampling sites (corresponding to the treatments of this experiment) were identified, and the collection methods were as follows (Manici et al. 2018): (1) Soil was collected within the row (10 to 20 cm deep in a 30-cm-diameter area with the trunk at the center in five sites per orchard) from the root zone of trees that exhibited decline symptoms, such as reduced plant vigor (characterized by lower yield and fruit quality than the orchard standard and leaf chlorosis) or death (symptomatic trees). Soil samples from the ABG region are hereafter referred to as T-ABG, while those from the NL region are termed T-NL. (2) Soil was collected within the tree row as described above from the root zone of trees showing optimal growth and high-quality production (asymptomatic trees). Soil samples from the ABG region are hereafter referred to as J-ABG, while those from the NL region are termed J-NL. (3) Fine roots (i.e., feeder roots with a diameter of less than 2 mm and non-woody structure) from the zone where the propagules of soilborne plant pathogens were most dense (10 to 20 cm depth and 30 cm from the trunk) were exposed and excised at five points around each tree showing ARD symptoms (Tilston et al. 2018). A total of nine samples were collected per orchard: six rhizosphere soil samples (three from healthy apple trees and three from trees showing ARD symptoms) and three root samples. After sieving, mixing, and removing impurities, the samples were sealed into bags, and the collection site details were recorded. At each location, GPS software was used to record the geographical coordinates (latitude and longitude). The tree height and main stem circumference 5 cm above the ground were measured on-site. The soil samples were labeled, placed on dry ice, and shipped to the laboratory as soon as possible. Samples for DNA extraction were stored at −80 °C, samples for fungal isolation were stored at −4 °C, and the remaining soil was sieved (50-mesh) for the determination of soil physicochemical properties and phenolic acids (Shen et al. 2020). All tools and equipment were sterilized with 75% ethanol before sample collection at each site. The remaining bulk orchard samples were stored moist in plastic-lined totes at 5 °C and used for ARD bioassays in the greenhouse (Ali et al. 2024).

**Table 7:** Selected plant growth indicators (plant height and ground diameter) for healthy trees without obvious symptoms of apple replant disease (ARD) and trees exhibiting symptoms attributed to ARD^a^

Plant growth indicators	Plant height (m)			Ground diameter (cm)		
Location number^b^	ARD symptomatic	Healthy	*P* value	ARD symptomatic	Healthy	*P* value
MC	2.05 (0.01)	2.18 (0.03)	0.0013	22.20 (0.52)	36.00 (2.00)	0.0003
PC	1.99 (0.10)	2.41 (0.16)	0.0172	13.70 (0.85)	17.53 (1.62)	0.0221
LC	1.61 (0.17)	2.53 (0.13)	0.0019	10.43 (0.75)	18.63 (1.26)	0.0006
QC	1.81 (0.04)	2.35 (0.30)	0.0370	11.83 (0.85)	21.33 (0.38)	0.0001
YL	1.11 (0.47)	2.67 (0.68)	0.0308	34.00 (0.87)	54.57 (10.60)	0.0285
DL	1.41 (0.19)	2.64 (0.23)	0.0020	83.17 (10.30)	113.67 (9.61)	0.0199
DC	2.55 (0.14)	2.89 (0.14)	0.0415	25.17 (3.25)	34.70 (4.30)	0.0376
JC	2.14 (0.23)	2.99 (0.12)	0.0050	13.27 (0.87)	17.00 (1.74)	0.0295
HC	1.83 (0.14)	3.03 (0.05)	0.0002	13.10 (0.75)	18.03 (0.68)	0.0011
H	1.91 (0.21)	3.54 (0.49)	0.0062	12.53 (3.27)	20.23 (1.17)	0.0184
XQ	1.65 (0.11)	2.45 (0.07)	0.0005	12.27 (0.25)	16.50 (0.95)	0.0017
XC	1.73 (0.06)	2.96 (0.11)	0.0001	13.23 (0.25)	19.87 (2.63)	0.0122
XJ	1.71 (0.07)	2.81 (0.10)	0.0001	13.47 (0.60)	17.90 (1.35)	0.0065
XL	1.79 (0.08)	2.42 (0.11)	0.0013	14.50 (0.89)	20.73 (0.32)	0.0003
XF	1.86 (0.04)	2.65 (0.10)	0.0002	15.50 (0.89)	22.43 (0.50)	0.0003
XW	2.20 (0.11)	2.95 (0.09)	0.0008	12.67 (0.40)	17.23(1.31)	0.0044

^a^All values are the mean of three replicates with the standard deviation of mean given in parentheses

^b^The specific meaning of the number can be found in Supplementary Table 1

### Determination of soil index

Available phosphorus (AP), available potassium (AK), available nitrogen (AN), and soil organic matter content (SOM) were determined according to the *Soil Physical and Chemical Analysis Experiment Guide* (Peverill et al. 1999). Soil bulk density (ρb) was determined using the ring knife method, and soil moisture content (ω) was measured using the drying method at 105 °C. Soil pH was measured using a Shanghai Lei Magnetic Benchtop pH Meter (model PHS-3EJ). Soil mechanical composition was measured using the hydrometer method (Avery 1973), and phenolic acid concentration was determined according to the method described by Chen et al. (2019).

### Severity assessment of ARD

Apple seedlings (*Malus hupehensis* Rehd.) were used in bioassays to confirm the presence of ARD and establish the relative degree of severity between the experimental orchard soils according to the method of Ali et al. (2024) and Xiang et al. (2021b). High-temperature sterilization of the 16 orchard soils was accomplished by exposing the moist soil to a high-pressure steam sterilizer (TOMY, Tokyo, Japan) at a temperature of 121 °C for 2 h across 2 cycles, each 24 h apart. The seeds of *Malus hupehensis* Rehd. were layered at 4 °C for approximately 30 d. Seeds with full buds and cracks were selected and sown in 50-hole seedling trays. After 1 month, seedlings with uniform growth were planted into 32 cm × 25 cm flower pots. After 30 d of cultivation, disease-free plants with consistent growth were selected for further planting. Seedlings were subsequently selected for uniformity and transplanted into pots (20 cm deep, 14 cm diameter, purchased from Tai'an Farmers Market) containing either high-temperature sterilized (h) or untreated (nh) orchard soil. The plants were grown in a greenhouse for 120 d. The inoculated plants were maintained in a greenhouse at 25–30 °C with a 12 h light/12 h dark cycle, and uniform watering management was applied to ensure normal plant growth. The severity of ARD for each soil was determined by calculating the ratio of dry mean weight from the high-temperature sterilized soil treatment and the untreated replanted soil for each orchard (Xiang et al. 2021b). The formula used was: %R = 100 × (X_h_-X_nh_)/X_nh_, where X_h_ and X_nh_ are the dry biomass accumulations for the high-temperature sterilized and untreated soil treatments, respectively. The severity of ARD in the experimental orchards was ranked as follows: Severe (%R > 100%), Moderate (%R = 50 to 100%), and Low (%R < 50%).

### Soil DNA extraction

The extraction of total DNA from rhizosphere soil was performed using the DNeasy PowerMax Soil Kit (Qiagen, Germany) according to the manufacturer’s instructions. The concentration and purity of the extracted DNA were assessed, and the standards used for ITS amplification followed those described by Shen et al. (2020).

### Quantitative Real-Time PCR (Q-PCR)

The relative abundance of *F. oxysporum*, *F. verticillioides*, *F. proliferatum*, and *F. solani* in the rhizosphere soil was measured with a CFX96 Touch™ Real-Time PCR Detection System (Bio-Rad, USA) with the primer sets JR/JF (*F. oxysporum*), CHR/CHF (*F. verticillioides*), CR/CF (*F. proliferatum*), and FR/FF (*F. solani*) (Duan et al. 2021). The PCR reaction system, procedure, and data processing method followed the description of Duan et al. (2021).

### Illumina MiSeq sequencing

The ITS rRNA genes were amplified using the primers ITS1F (5′-CTTGGTCATTTAGAGGAAGTAA-3′) and ITS2R (5′-GCTGCGTTCTTCATCGATGC-3′) in a thermocycler PCR system (GeneAmp 9700, ABI, Foster, CA, United States) (Trofymow et al. 2020). The PCR reactions were conducted using the following program: 3 min of denaturation at 95 °C, 35 cycles of 30 s at 95 °C, 30 s for annealing at 55 °C, and 45 s for elongation at 72 °C, followed by a final extension at 72 °C for 10 min. The PCR reactions were performed in triplicate with a 20 μL mixture containing 4 μL of 5 × FastPfu Buffer, 2 μL of 2.5 mM dNTPs, 0.8 μL of each primer (5 μM), 0.4 μL of FastPfu Polymerase, 0.2 μL of BSA and 10 ng of template DNA. The PCR products were extracted from a 2% agarose gel, purified using an AxyPrep DNA Gel Extraction Kit (Axygen Biosciences, Union City, CA,United States), and quantified using QuantiFluorTM-ST (Promega, Madison, WI, United States) according to the manufacturer’s instructions. Purified amplicons were pooled in equimolar concentrations and paired-end sequenced (2 × 300) on an Illumina MiSeq platform (Illumina, San Diego, CA, United States) according to the standard protocols by Majorbio Bio-Pharm Technology Co. Ltd. (Shanghai, China) (Liu et al. 2019).

The obtained raw reads were subjected to quality filtering, OTU clustering, ITS gene sequence classification, and alpha diversity analysis according to the method of Jiang et al. (2017). Krona software was used to visualize the species annotation results (Awasthi et al. 2017). Fungal function prediction was performed using the FUNGuild database (Nguyen et al. 2016). RDA analysis was conducted using CANOCO5.0. Spearman analysis was performed using the Genescloud tool (https://www.genescloud.cn. The correlations between environmental variables and the groups of dry weight inhibition rate were determined using the correlation test in SPSS (version 20.0, SPSS Inc., Chicago, USA). Bioinformatics analysis was performed using the Majorbio platform (https://www.majorbio.com) (Shen et al. 2020).

### Fungal isolation

Pathogenic microorganisms were isolated from the rhizosphere soil using the plate dilution coating method as previously described (Nsa et al. 2020).The details are as follows: 10 g of rhizosphere soil was re-suspended in 90 mL of sterilized water and shaken at 180 r·min^−1^ for 30 min at 26 °C to fully dissolve the fungi in the soil. The sample was left to stand for 45 s to prepare a stock suspension of soil fungi. The suspension was diluted to 10^–5^, and 100 μL of each dilution was plated on the culture medium (Table S2) and incubated at 25 °C for 24–72 h. Each treatment was repeated three times.

Pathogenic microorganisms were also isolated from roots following the method of Zhou et al. (2015). The details are as follows: typical diseased roots from orchard trees were washed under tap water to remove adhering soil, followed by dipping in 75% ethanol (V/V) for 30 s and then in 1% NaOCl (W/V) for 10 s. The samples were subsequently blotted dry on sterilized paper towels in a laminar flow hood. A sterilized scalpel was used to cut the diseased tissue into small pieces at the junction of the diseased and healthy tissue. Five root pieces (3–5 mm^2^) were randomly taken from each of three plant root segments in each replicate, resulting in 75 root pieces per treatment and a total of 225 segments per orchard. Sterile filter paper was used to absorb the water on the surface of the tissue block, which was then transferred to the culture medium (Table S2). There were 5 tissue pieces per dish, with 6 dishes in total. The isolation plates were incubated in the dark at 25 °C for 48–72 h. Hyphae growing around the diseased tissue were picked out under aseptic conditions. Individual colonies that developed on the media were transferred to water agar (WA). Under a sterile operating table, single spores were picked under a stereomicroscope and inoculated on a PDA plate. After incubating at 25 °C for 5 d, a mycelial disk (0.5 cm in diameter) was placed in a freezing tube containing a 15% glycerol sterile water mixture and stored in a refrigerator at −80 °C for species identification and pathogenicity assays.

### Morphological identification and phylogenetic analysis

Morphological identification and phylogenetic analysis were performed according to the methods described in Table S3.

Total fungal DNA was extracted from pure cultures as described by Amatulli et al. (2010). Approximately 100 mg of mycelium was scraped from each strain growing on PDA medium, and DNA was extracted using the Fungus Genomic DNA Extraction Kit (Solarbio, Beijing, China) according to the manufacturer’s instructions. The DNA regions and primers used for molecular identification are described in Table S4. Polymerase chain reaction (PCR) amplification was performed in an Applied Biosystems 2720 Thermal Cycler (Applied Biosystems, U.S.A.). The primers and PCR cycles are presented in Supplementary Table S4. Amplified PCR products were purified by using a DNA Gel Recovery Kit (AxyPrep, Hangzhou, China) and sequenced on an ABI 3730XL (Applied Biosystems, U.S.A.) by Qingdao Passeno Gene Biotechnology Co., Ltd. Phylogenetic analysis based on maximum likelihood was performed as previously described (Duan et al. 2021), and all sequences were downloaded from NCBI GenBank with accession numbers listed in Table S5-S10.

### Pathogenicity assays

The greenhouse experiments were conducted at Shandong Agricultural University, China, during 2019–2020. The soil was obtained from a 31-year-old apple orchard in Manzhuang Town, Taian, China (117.081039°E longitude, 36.06682°N latitude). The apple rootstock M.9T337 was purchased from Shandong Huinong Horticultural Technology Co., Ltd. The seeds of *Malus hupehensis* Rehd. were layered at 4 °C for approximately 30 d. The acquisition of *Malus hupehensis* Rehd. was as previously described.

A total of 4186 fungal strains were isolated. Among these, 1228 strains (89 genera) (data not provided) were obtained through morphological identification and ITS sequencing for pathogenicity assays. Nine seedlings of each cultivar (M.9T337 and *Malus hupehensis* Rehd.) at the 6-leaf stage were used for pathogenicity testing of isolates. The specific root inoculation method, using a conidia suspension (10^6^ spores·mL^−1^) as the test material and the modified dip-and-cut technique, was as previously described (Sun et al. 2020). The preparation of the conidia suspension is detailed in Table S11. The treated seedlings were replanted in new pots (upper diameter 7 cm × 7 cm, height 8 cm, bottom diameter 5 cm) filled with a sterilized mixture (80 mL vermiculite first, followed by 240 mL of soil) and finally covered with 80 mL of sterile vermiculite. The pots were placed on a 24-hole tray (43 cm × 28.8 cm × 5 cm), which was then placed on an outer tray (44 cm × 32 cm × 3 cm). The inoculated plants were maintained in a greenhouse at 25–30 °C with a 12-h light/12-h dark cycle, and uniform watering management was applied to ensure normal plant growth. The symptoms of ARD were examined 2 weeks after inoculation. The disease index was calculated according to the disease severity criteria described by Purwati et al. (2008). The validation of Koch’s postulates was performed as previously described (Wu et al. 2019).

### Co-inoculation of *Aspergillus*, *Alternaria*, *Penicillium*, and *Fusarium*

In addition, we conducted a pathogenicity test to compare the aggressiveness of the most abundant root pathogenic fungi recovered in this study and to evaluate their relative ability to colonize apple roots. The methodology was the same as that adopted in the pathogenicity tests for isolated strains. Eight treatments were compared: FF (*F. oxysporum* YR15, HC131; *F. solani* HC39, Q61; *F. proliferatum* MR5); FF + AF (*Aspergillus flavus* XW23); FF + AA (*Alternaria alternata* YR9); FF + PB (*Penicillium brasilianum* Q9); AF (*Aspergillus flavus* XW23); AA (*Alternaria alternata* YR9); PB (*Penicillium brasilianum* Q9); Uninoculated control. In co-inoculations, a mixture of each isolated spore suspension in a 1:1 ratio was used for inoculation.

### Fungicide susceptibility testing

Further research by the authors found that *Fusarium proliferatum* MR5 f.sp. *malus domestica* (*Fpmd* MR5) was the leading pathogen causing the occurrence of ARD in China (Duan et al. 2021). Therefore, to identify fungal fungicides effective for controlling *Fpmd* MR5, the inhibitory effects of 11 fungal fungicides on the mycelial growth and conidia germination of *Fpmd* MR5 were determined. For sensitivity assays, a mycelial plug (5 mm in diameter) taken from the margin of a 3-day-old colony was placed on the center of a PDA plate treated with test fungicides at different concentrations, and the final concentrations are shown in Table S12. PDA medium with sterilized water/methanol was used as a blank control (Zheng et al. 2015). Three replicates were used for each concentration. Cultures were kept at 28 °C until the control plates covered the entire surface, and colony diameters were measured in two perpendicular directions; the diameter (5 mm) of the original mycelial plug was subtracted from each measurement (Duan et al. 2021).

The slide method was used to determine the inhibitory effect of fungicides on the conidia germination of *Fpmd* MR5, following Duan et al. (2021). *Fpmd* MR5 was cultured on VBC medium (1 g of KH_2_PO_4_, 1 g of KNO_3_, 0.5 g of sucrose, vitamin B1 tablet, vitamin C tablet, and 20 g of agar in 1 L of water) at 28 °C for 7 d, rinsed thoroughly with sterile water, and shaken evenly. The density of the conidial suspension was measured using a hemocytometer, and the suspension was diluted with sterile distilled water to a final concentration of 1 × 10^6^ spores·mL^−1^ (Duan et al. 2021). The conidia suspension was then mixed with different concentrations of fungicides on a concave glass slide at a ratio of 1:1, with sterile water used as a control. The slide was kept moist and incubated at 28 °C for 24 h to measure the spore germination rate. Spores were scored as germinated if the germ tube length equaled or exceeded half the diameter of the spore. The percentage inhibition was calculated using the formula: I = G/T × 100%, where I is the percent spore germination rate, G is the number of germinated spores, and T is the total number of spores. The total number of spores examined was greater than 200; three replicates were conducted for each treatment, and the experiment was repeated twice. Microsoft Excel 2013 (Microsoft Corporation, USA) was used for data processing, and the relationship between the logarithm of the concentration of each agent (the abscissa) and the mycelial growth inhibition rate and spore germination inhibition rate (the ordinate) was analyzed. Linear regression analysis was used to calculate the EC_50_ value of different agents against *Fpmd* MR5 and the correlation coefficient (*R*^*2*^). The specific calculation formula was based on that of Wu et al. (2014).

### Measurements of extracellular enzyme activity

Pectinase activity was detected by growing fungi in a petri plate on mineral salt agar medium (10 g of citrus pectin (Sigma)/5 g of CM-cellulose (Sigma, USA), 0.1 g of yeast extract, 0.2 g of NaNO_3_, 0.5 g of KCl, 0.5 g of MgSO_4_.7H_2_O, 1.0 g of K_2_HPO_4_, 0.01 g of FeSO_4_.7H_2_O, 20.0 g of agar, pH 6.8–7.0, and 1 L of distilled water). After incubation at 30 °C for 5 d, pectinase activity was tested on plates with orange/transparent halos around the colonies (Kwon et al. 2007; Reddy and Sreeramulu 2012). *Fpmd* MR5 (10^5^ CFU·mL^−1^) was inoculated in 100 mL of pectin mineral medium and incubated under the same conditions described above at 150 rpm. After culture incubation, the cells were centrifuged for 10 min at 15,288 × g (4 °C), and the crude enzyme extract was collected and used in the experiments. The activity of polygalacturonases (PGAse) was recorded several times (from 24 to 240 h).

PGAse activity was determined by measuring the reducing sugars produced by PGA in acetate buffer (0.2 M, pH 5.0) with DNS reagent. The reaction mixture contained a 2.5 g·L^−1^ PGA solution (2 mL) in acetate buffer (0.2 M, pH 5.0) and 50 μL of crude enzyme extract. All tubes were incubated in triplicate at 37 °C for 20 min. The reducing sugars formed were quantified using a calibration curve of GalA (y = 0.3008x + 0.0222, *R*^*2*^ = 0.9852). The reaction was then stopped by adding DNS reagent and boiling for 5 min. The absorbance was read at 540 nm using a spectrophotometer. One unit (U) was defined as the amount of enzyme that releases 1 μmol of GalA per minute (Junior et al. 2021).

Endo-1,4-β-glucanase activity (CMCellulase) was determined at 50 °C with 1% carboxymethylcellulose (Sigma) as substrate in acetate buffer (50 mM, pH 5.0), using glucose as standard (y = 865.41x + 102.17, *R*^*2*^ = 0.9968). The absorbance was read at 540 nm using a spectrophotometer. One enzymatic unit was defined as the amount of enzyme necessary to release 1 µmol of reducing sugars per minute under the aforementioned reaction conditions (Kikot et al. 2010). Enzymatic reactions were performed in a water bath with controlled temperatures (Shanghai, China), and the optical density was measured using a 2600 UV–Vis spectrophotometer (Shanghai, China).

### Data analysis

Soil physicochemical properties, plant biomass, and phenolic acid data are presented as the means ± one standard deviation (SD) of three replicates. Analysis of variance (ANOVA) was conducted, and the data were plotted using SPSS (version 20.0, SPSS Inc., Chicago, USA). The figures were plotted using Microsoft Excel 2013 (Microsoft Corporation, USA) and GraphPad Prism 7.0 (GraphPad, Inc., USA) (Duan et al. 2021). The least significant difference (LSD) test was used for comparison, and significant difference between differences samples were determined at the *p* < 0.05 level. SmartPLS 4.1.0.9 (SmartPLS GmbH, Hamburg, Germany) was used to perform partial least squares structural equation modelling (PLS-SEM) analysis to identify potential causal relationships between explanatory variables and the degree of apple replant disease. The appropriateness of the SEM was ensured by evaluating and optimizing the model fit (Hair et al. 2021).

## Supplementary Information

Supplementary Material 1.

Supplementary Material 2.

## Data Availability

All data analyzed during this study are included in this published article and the sequencing data were deposited to NCBI database. In addition, data will be made available on request. The raw reads can be found below: https://www.ncbi.nlm.nih.gov/bioproject/PRJNA780338.

## Abbreviations

*F. **solani*: *Fusarium* *solani*
*F. **oxysporum*: *Fusarium* *oxysporum*
*F. **proliferatum*: *Fusarium* *proliferatum*
*F. **verticillioides*: *Fusarium* *verticillioides*
*Fpmd *MR5: *Fusarium proliferatum* MR5 f.sp. *malus domestica*
CWDEs: Plant cell wall degrading enzymes
ARD: Apple replant disease
HRS: Rhizosphere fungal community of healthy apple trees
DRS: Rhizosphere fungal community of diseased apple trees
GPS: Global Positioning System
FUNGuild: Fungi Functional
NL: Northwest Loess region
ABG: Around Bohai Gulf region
RFC: Rhizosphere fungal community
AP: Available phosphorus
AK: Available potassium
AN: Available nitrogen
SOM: Organic matter content
ITS: Internal Transcribed Spacer
Q-PCR: Real-Time Quantitative PCR
PDA: Potato Dextrose Agar
WA: Water agar
PCR: Polymerase chain reaction
CM-cellulose: Carboxymethyl Cellulose
PGAse: Polygalacturonases
CMCellulase: Endo-1,4-β-glucanase activity
DNS: 3,5-Dinitrosalicylic acid
OTU: Operational Taxonomic Unit
SD: Standard deviation
LSD: Least significant difference
PLS-SEM: Partial least squares structural equation modelling
ω: Soil moisture content
St: Syringate
Pha: *p*-hydroxybenzoic acid
ρb: Soil bulk density
Ba: Benzoic acid
Ca: Cinnamic acid
Fa: Ferulic acid
Ch: Catechin
Pd: Phloridin
LEfSe analysis: Linear discriminant analysis Effect Size
NMDS: Non-Metric Multidimensional Scaling
