# Supplementary material for: Apple replant disease: unraveling the fungal enigma hidden in the rhizosphere
Source: Stress Biol. 2025 Nov 27;5(1):71. doi: 10.1007/s44154-025-00258-1 (PMC12657686; doi:10.1007/s44154-025-00258-1)

Supplementary materials

Supplementary Figure 1. Replant disease-like symptoms (H-R) were limited to some foci within orchards that otherwise showed homogeneous growth and standard production (A-G). These symptoms included reduced plant vigor (characterized by lower yield, poor fruit quality compared to the orchard standard, and leaf chlorosis) or plant death.

Supplementary Figure 2. A, D, G: Multi-sample rarefaction curves based on OTU clustering data of rhizosphere soil fungi in replanted orchards. The abscissa represents the number of sequencing sequences randomly selected from a sample, and the ordinate represents the number of OTUs constructed based on the sequencing depth. Each color corresponds to a sample type. B, E, H: Rank-abundance distribution curves of rhizosphere soil fungi. The abscissa indicates the ordinal rank of OTUs sorted by abundance, and the ordinate indicates the relative abundance of OTUs. Each color represents a sample type. C, F, I: Species accumulation curves of rhizosphere soil fungi. A-C: Rhizosphere soil samples from diseased apple trees in the Northwest Loess region (NL). D-F: Rhizosphere soil samples from diseased apple trees in the Around Bohai Gulf region (ABG). G-I: Rhizosphere soil samples from healthy apple trees in the ABG region.

Supplementary Figure 3. OTU-Venn graph of rhizospheric soil samples from replanted orchards in ABG and NL. A: Rhizosphere soil samples from diseased apple trees in ABG; B: Rhizosphere soil samples from healthy apple trees in ABG; C: Rhizosphere soil samples from diseased apple trees in NL; D: OTU-Venn graph of rhizospheric soil samples from healthy (J-) and diseased (T-) apple trees in ABG. H: OTU-Venn graph of rhizospheric soil samples from diseased apple trees in ABG and NL. E-G, I-O: OTU-Venn graph of rhizospheric soil samples from healthy (J-) and diseased (T-) apple trees in each replanted orchard in ABG (HC1, HC2, and HC3 represent the three replicates of rhizospheric soil samples from diseased apple trees; HJ1, HJ2, and HJ3 represent the three replicates of rhizospheric soil samples from healthy apple trees, and so on). Different colors represent different samples (or groups), and the outermost circle indicates the sample name. The petals include two rows of numbers: the upper row shows the total number of OTUs in each sample, and the lower row (in brackets) indicates the number of unique OTUs for each sample. The white circle in the center represents the number of core OTUs.


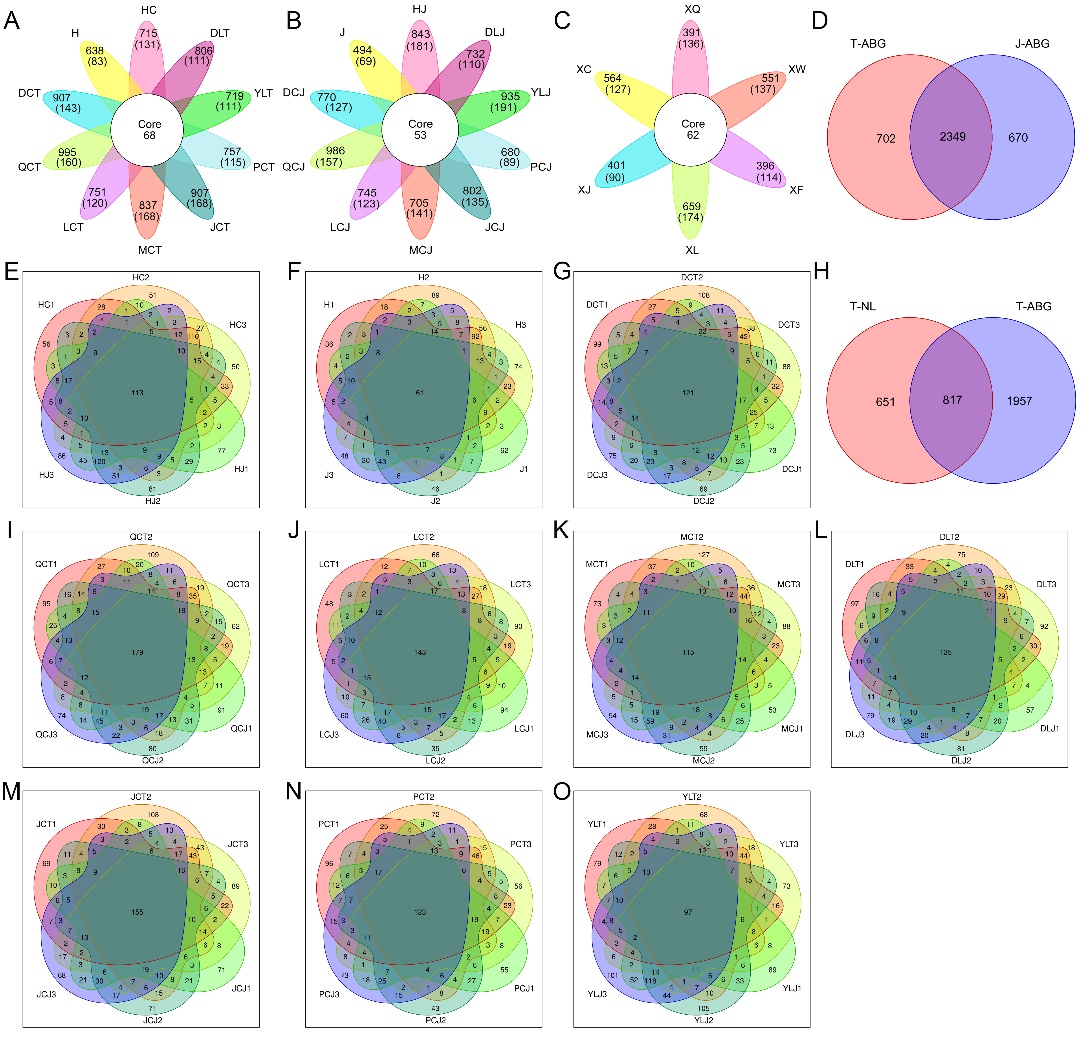


Supplementary Figure 4. Relative abundances of fungal taxa in rhizospheric soil samples from diseased apple trees at the phylum level. A: Replanted orchards in the NL (T-NL). B: Replanted orchards in the ABG (T-ABG). C: Comparison of replanted orchards in the NL and ABG. D: Heatmap showing the relative abundance of phyla in different samples (variables clustered on the vertical axis). The phylogenetic tree was constructed using the neighbour-joining method. The colour intensity corresponds to the relative abundance of the dominant phyla.


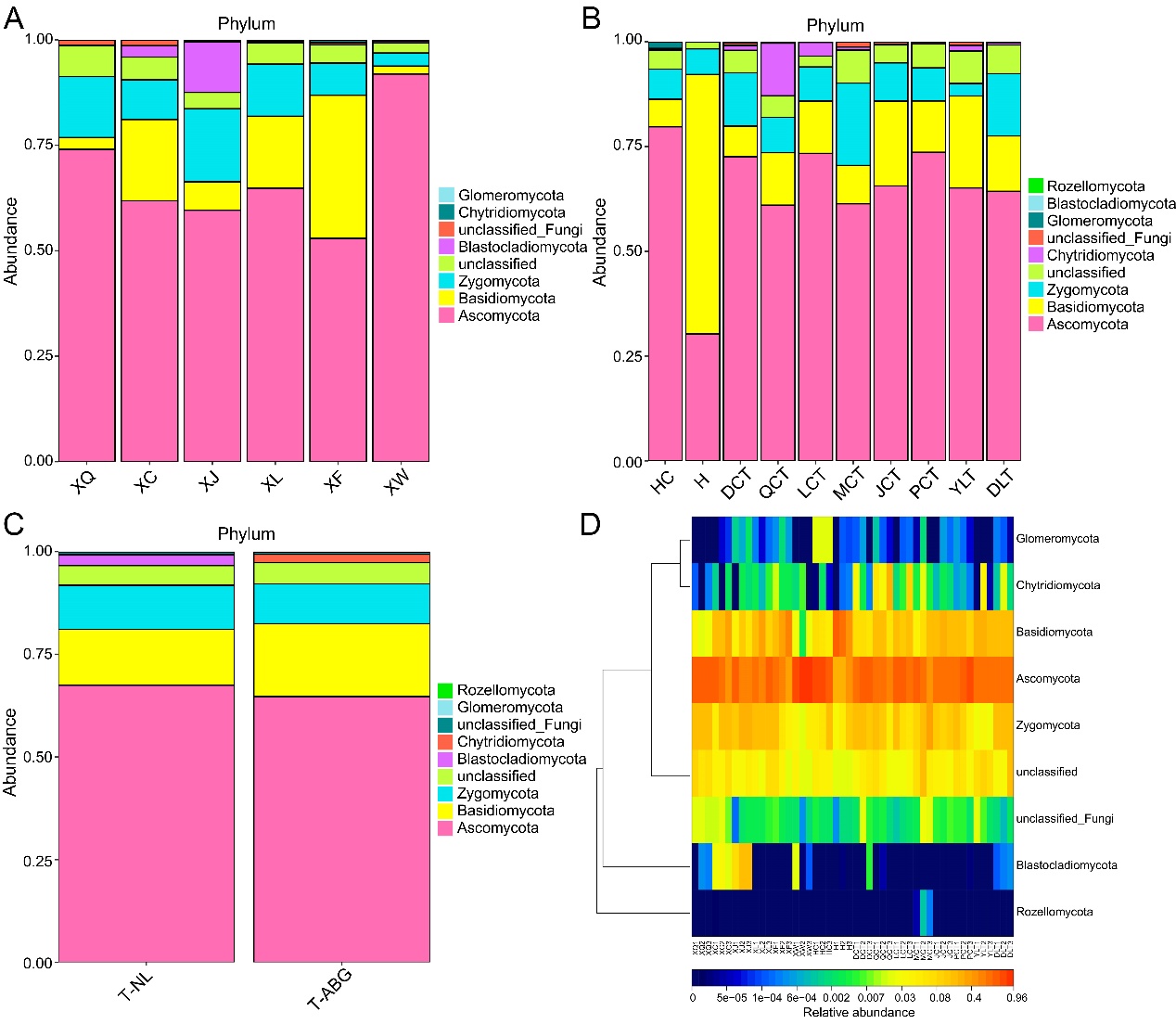


Supplementary Figure 5. Relative abundances of fungal taxa in rhizospheric soil samples from diseased apple trees at the family and genus levels. A-B: Replanted orchards in the NL (T-NL). C-D: Replanted orchards in the ABG (T-ABG). E-F: Comparison of replanted orchards in the NL and ABG. The relative abundance of each species with less than 0.5% in each sample is combined into "Others." G-H: Heatmap showing the relative abundance of families or genera in different samples (variables clustered on the vertical axis). The phylogenetic tree was constructed using the Bray-Curtis method. The colour intensity corresponds to the relative abundance of the dominant family or genus.


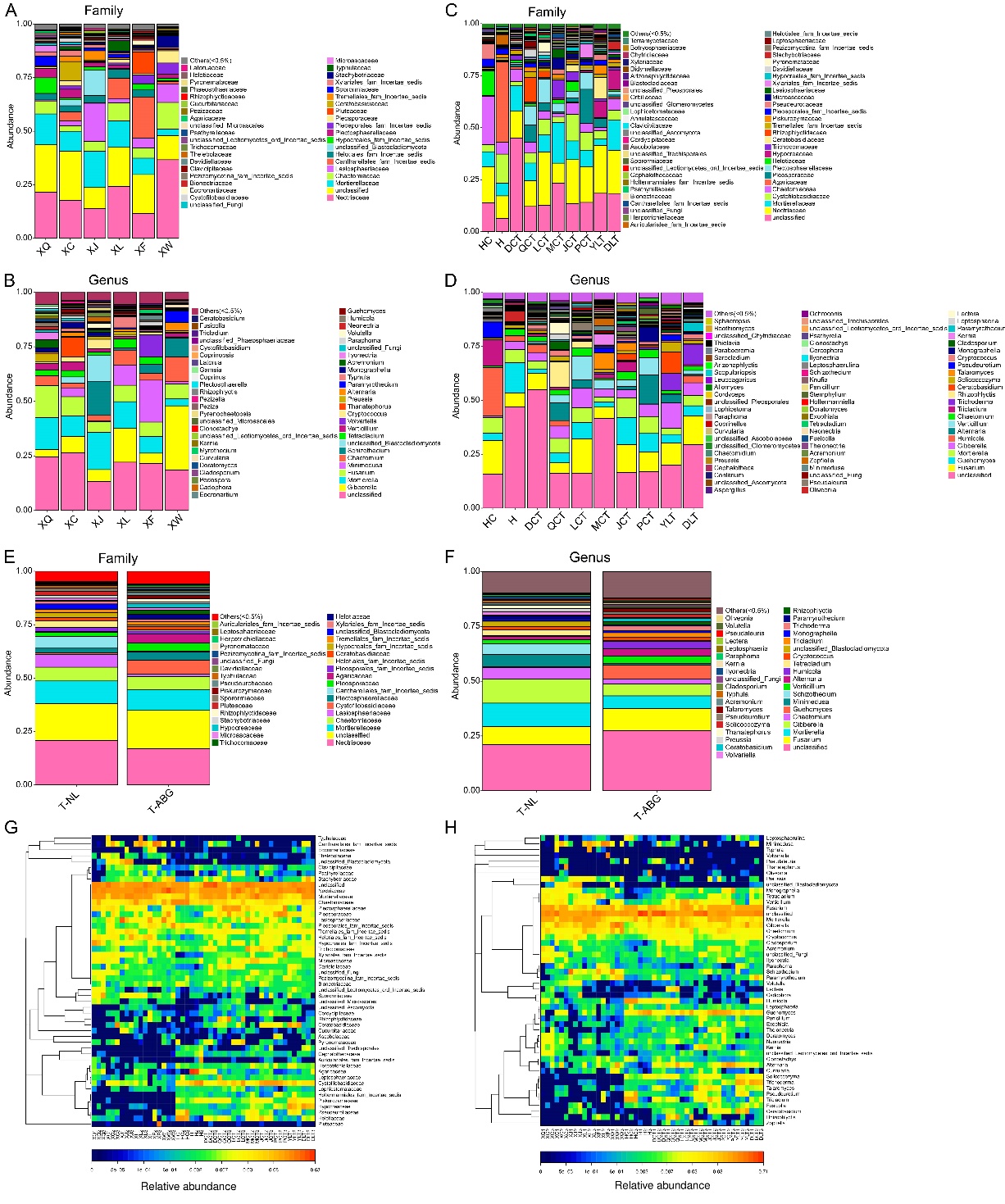


Supplementary Figure 6. Krona analysis of fungal taxa in rhizospheric soil of replanted orchards in the ABG and NL. The circles represent different taxonomic levels from the outside to the inside (Phylum, class, order, family, genus, species), and the area of sector means respective proportion of different OTUs annotation results.


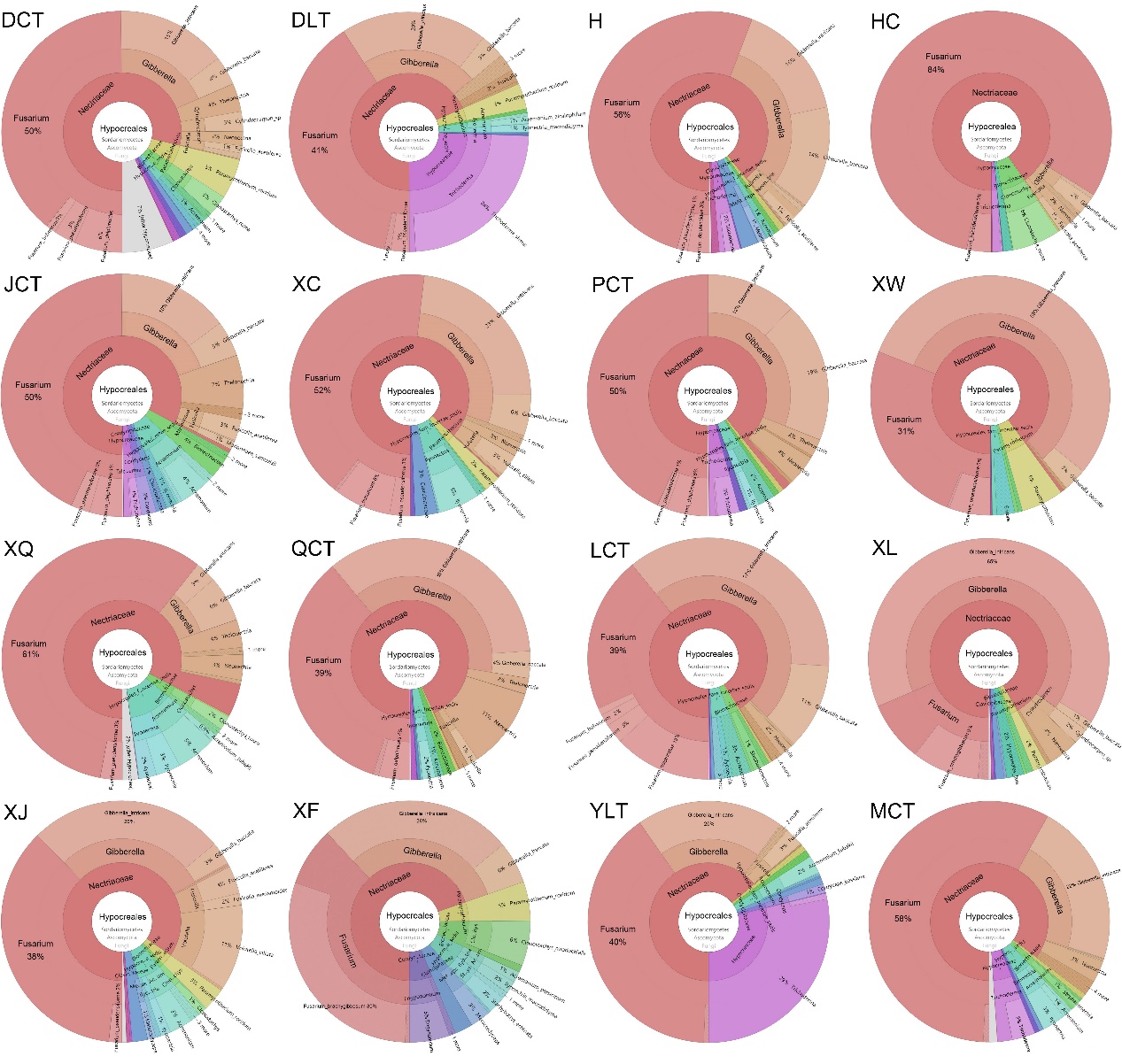


Supplementary Figure 7. Relationship between the rate of inhibition of plant dry weight and main soil properties. The regression lines indicate statistical significance at *P*<0.05.


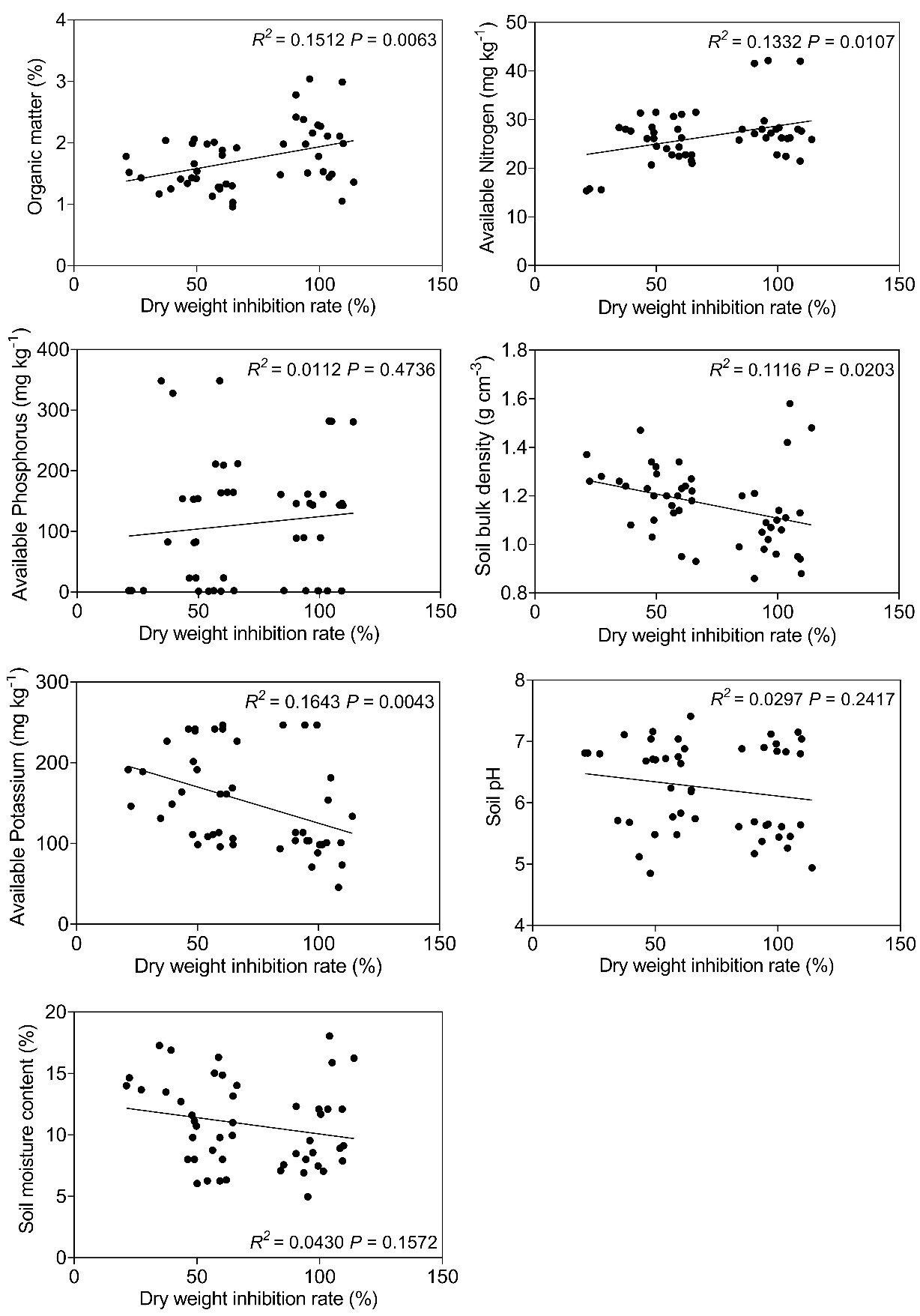


Supplementary Figure 8. Relationship between the rate of inhibition of plant dry weight and main soil phenolic acids. The regression lines indicate statistical significance at *P* <0.05.


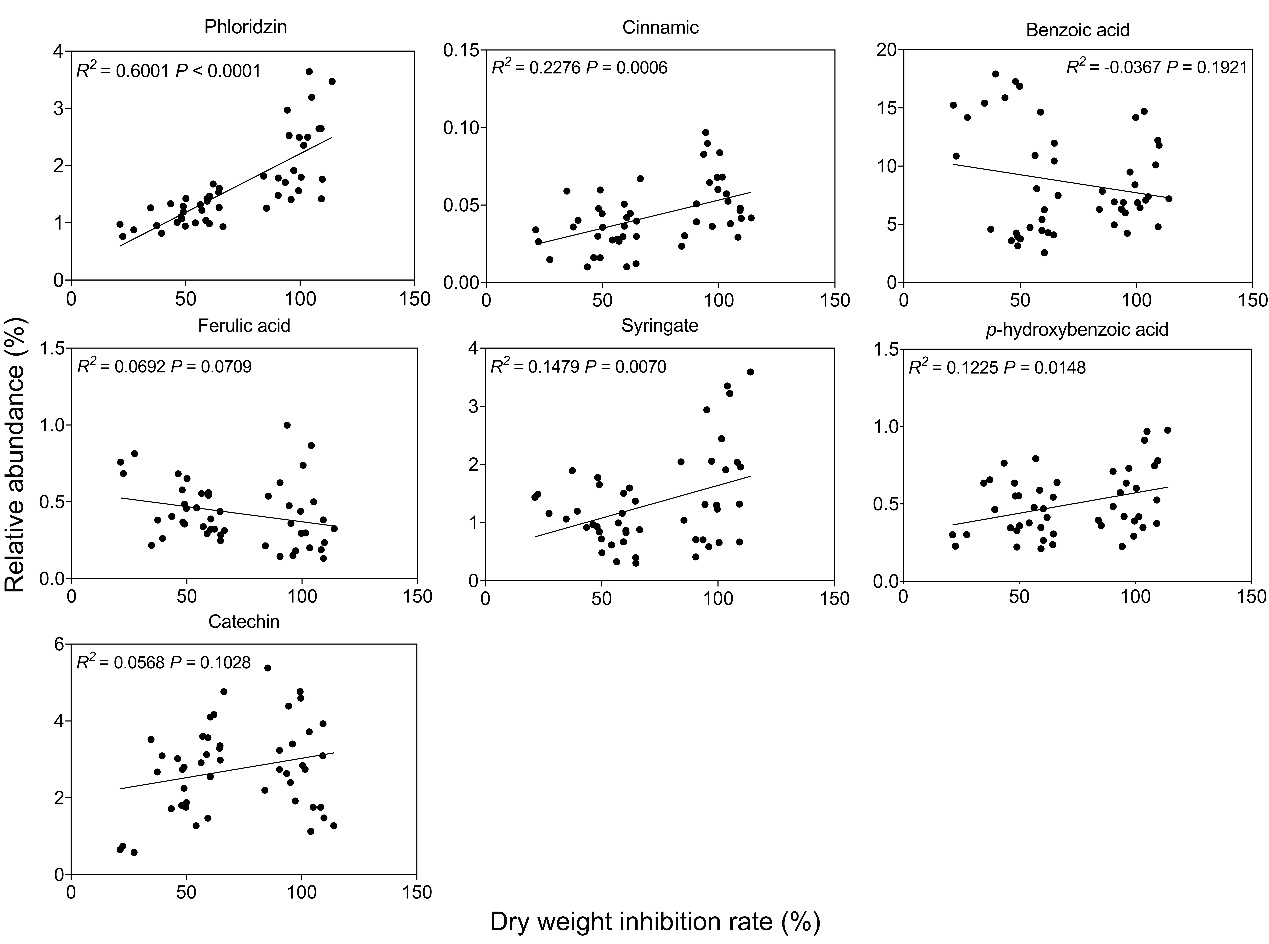


Supplementary Figure 9. Relative abundances of fungal taxa at the family level in rhizospheric soil samples from replanted orchards in the ABG. A: Rhizospheric soil samples from healthy apple trees. B: Comparison of fungal taxa in rhizospheric soil samples from diseased and healthy apple trees. C: Heatmap showing the relative abundance of families in different samples (variables clustered on the vertical axis). The phylogenetic tree was constructed using the neighbour-joining method. The colour intensity corresponds to the relative abundance of the dominant family. D-M: Comparison of fungal taxa in rhizospheric soil samples from diseased and healthy apple trees in each replanted orchard. UPGMA cluster analysis was performed using the Bray-Curtis method, and the clustering results were integrated with the species' relative abundance in each sample. The left side shows the UPGMA clustering tree structure, and the right side displays the species' relative abundance distribution for each sample.


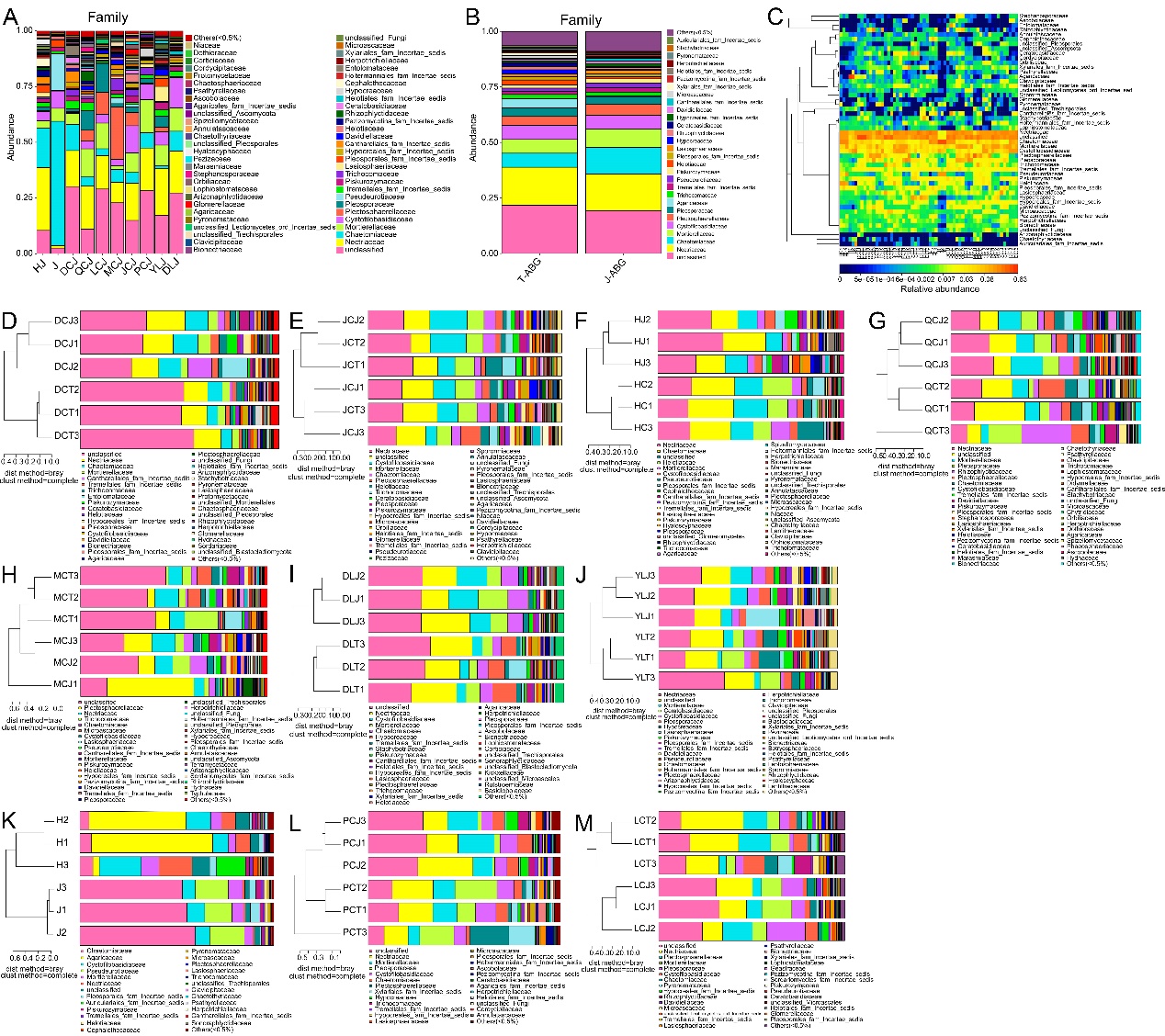


Supplementary Figure 10. Relative abundances of fungal taxa at the phylum level in rhizospheric soil samples from replanted orchards in the ABG. A: Rhizospheric soil samples from healthy apple trees. B: Comparison of fungal taxa in rhizospheric soil samples from diseased and healthy apple trees. C: Heatmap showing the relative abundance of phyla in different samples (variables clustered on the vertical axis). The phylogenetic tree was constructed using the neighbour-joining method. The colour intensity corresponds to the relative abundance of the dominant phylum. D-M: Comparison of fungal taxa in rhizospheric soil samples from diseased and healthy apple trees in each replanted orchard. UPGMA cluster analysis was performed using the Bray-Curtis method, and the clustering results were integrated with the species' relative abundance in each sample. The left side shows the UPGMA clustering tree structure, and the right side displays the species' relative abundance distribution for each sample.


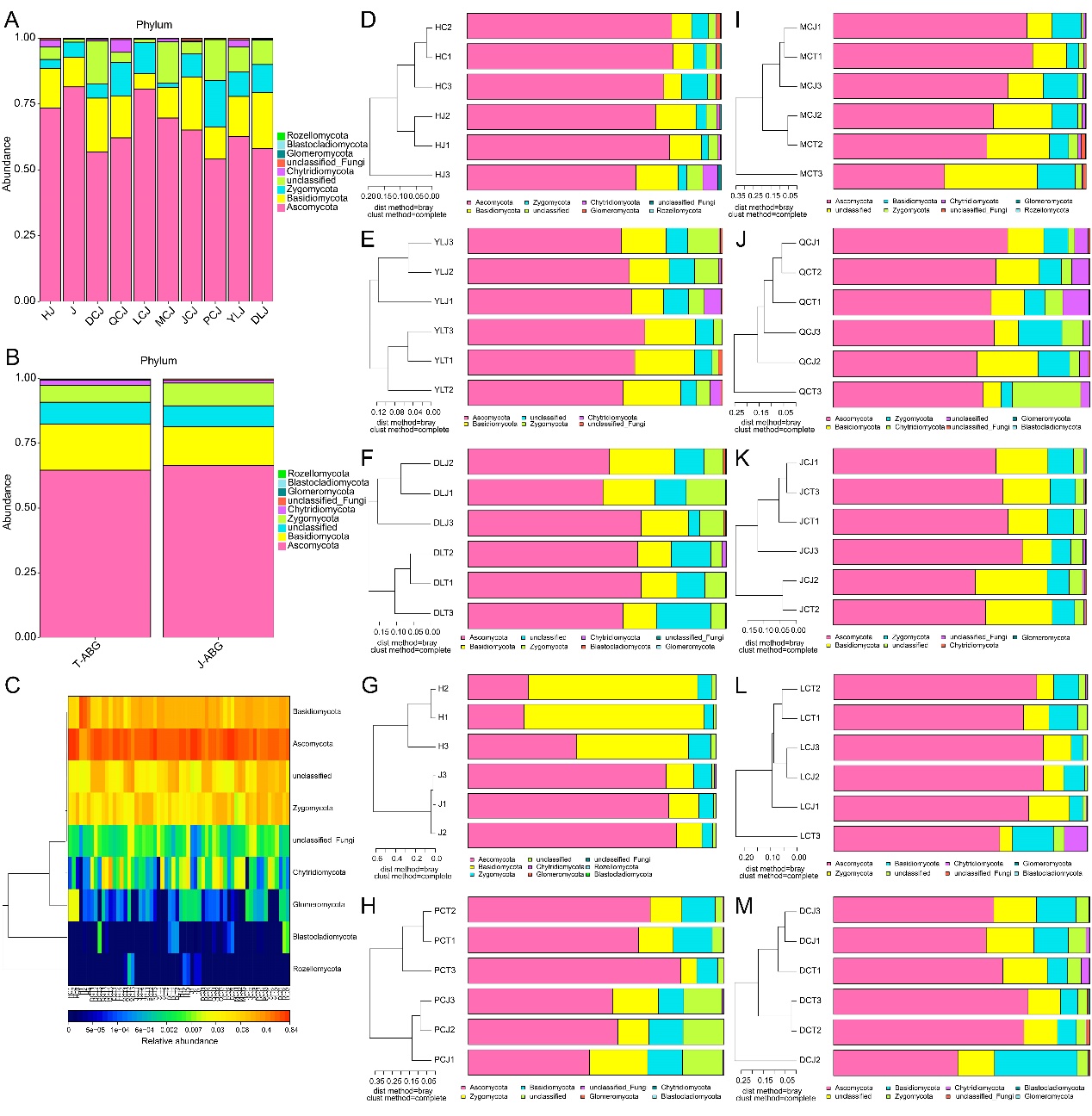


Supplementary Figure 11. Relative abundances of fungal taxa at the genus level in rhizospheric soil samples from replanted orchards in the ABG. A: Rhizospheric soil samples from healthy apple trees. B: Comparison of fungal taxa in rhizospheric soil samples from diseased and healthy apple trees. C: Heatmap showing the relative abundance of genera in different samples (variables clustered on the vertical axis). The phylogenetic tree was constructed using the neighbour-joining method. The colour intensity corresponds to the relative abundance of the dominant genus. D-M: Comparison of fungal taxa in rhizospheric soil samples from diseased and healthy apple trees in each replanted orchard. UPGMA cluster analysis was performed using the unweighted Unifrac distance matrix, and the clustering results were integrated with the species' relative abundance in each sample. The left side shows the UPGMA clustering tree structure, and the right side displays the species' relative abundance distribution for each sample.


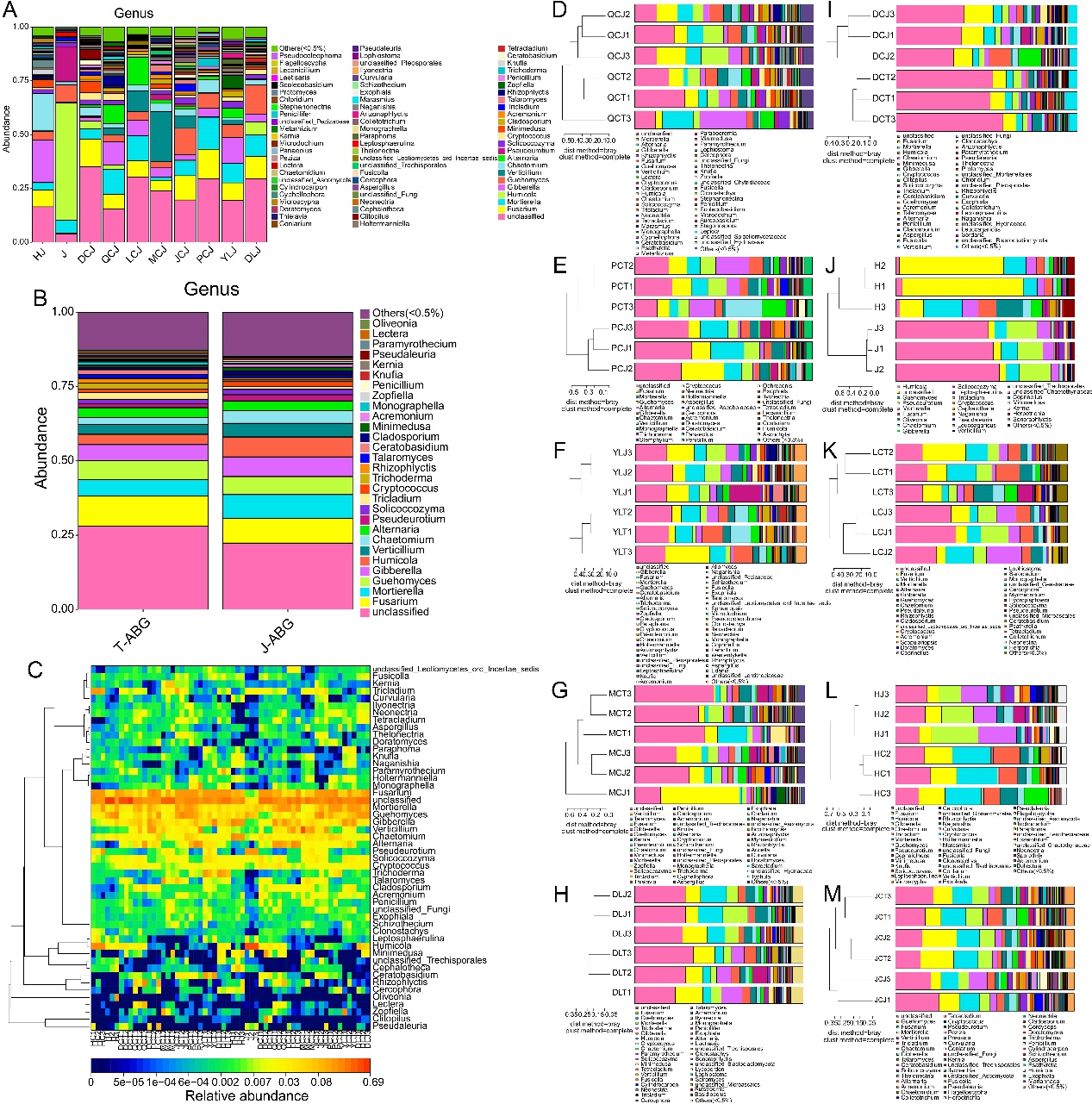


Supplementary Figure 12. Hierarchical clustering tree (A), difference matrix heatmap (B), and non-metric multidimensional scaling (NMDS) results (C) for fungal communities at the OTU level in rhizospheric soil from replanted orchards in the ABG and NL, analyzed using the unweighted UniFrac method. Different colored dots represent different sample groups.


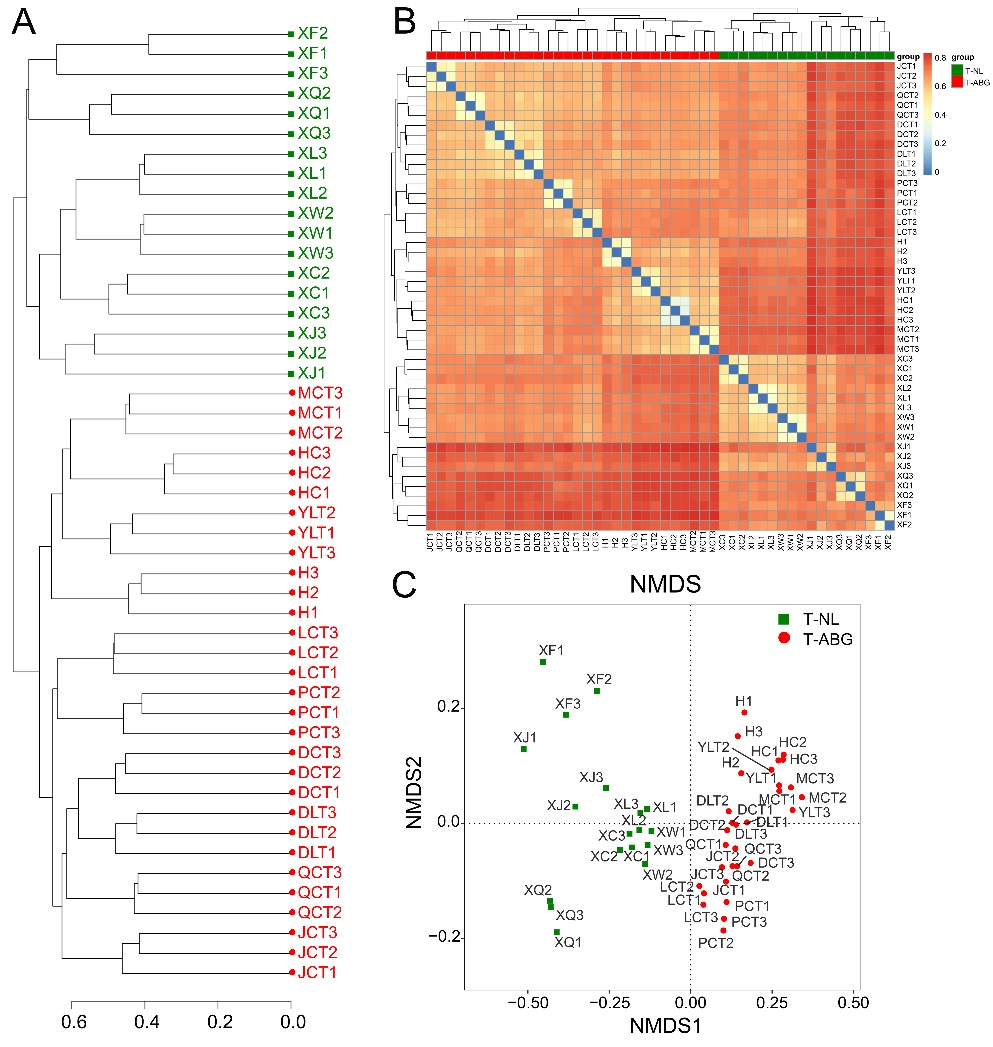


Supplementary Figure 13. Bar chart generated from the LEfSe analysis. The top note of the figure displays the differential species information. The ordinate represents the relative abundance, and the abscissa indicates the sample name (each column corresponds to a sample; only the group name is labeled in the figure, not the individual sample names). Different groups are highlighted in yellow boxes. T-NL: Rhizospheric soil samples from replanted orchards in NL. T-ABG: Rhizospheric soil samples from replanted orchards in ABG.


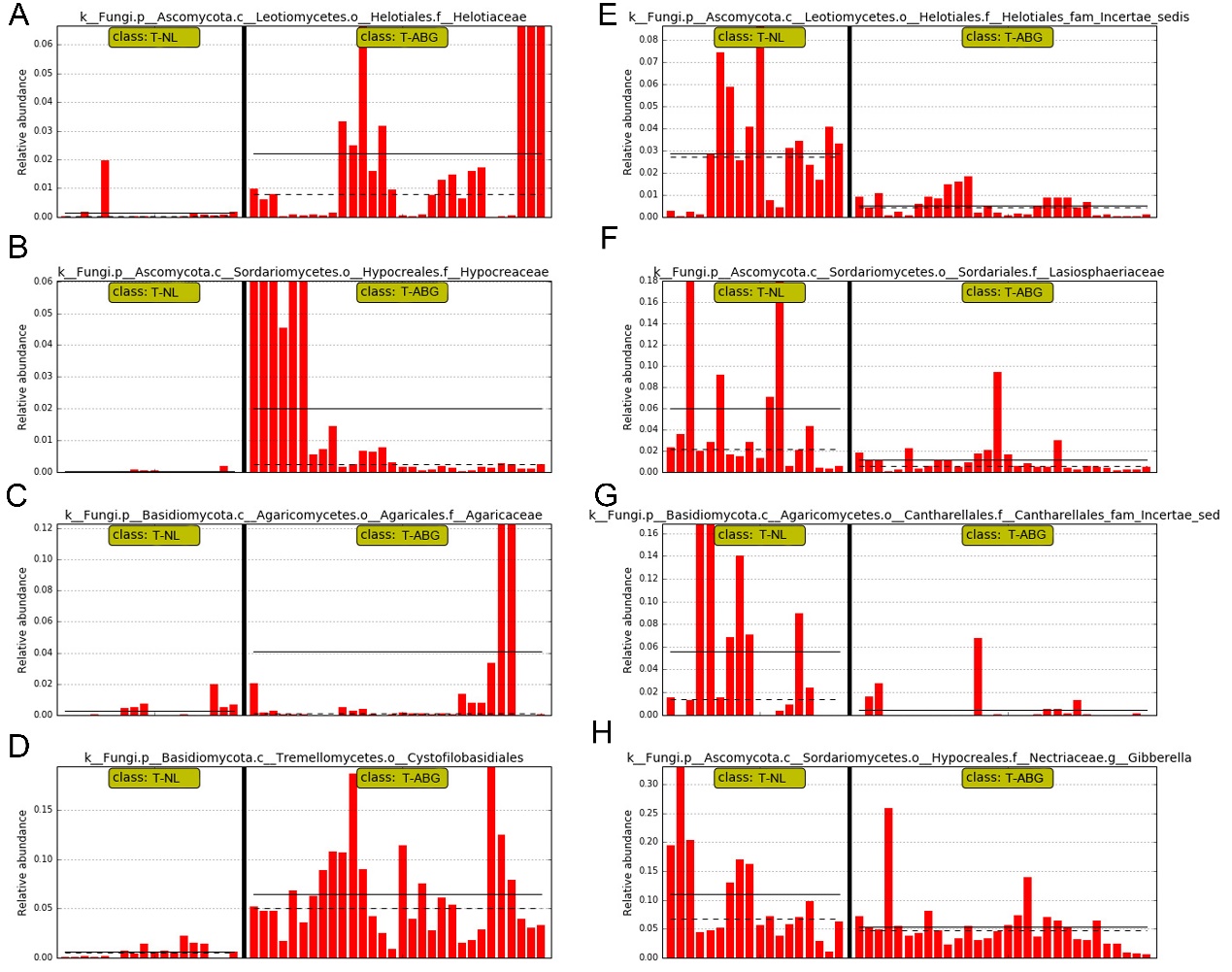


Supplementary Figure 14. A-B: Metastats analysis of fungal taxa at the family (A) and genus (B) levels in rhizospheric soil from replanted orchards in the ABG and NL. Each column represents the relative abundance of the differential species. C-D: ANOVA-T test was used to analyze the differences in relative abundance of fungal taxa in rhizospheric soil between diseased and healthy apple trees in the ABG. The top 20 species (*p*<0.05) were selected for histogram plotting. Significance levels are indicated as follows: *0.01 < *p* < 0.05, **0.001 < *p* < 0.01, ***0.0001 < *p* < 0.001.


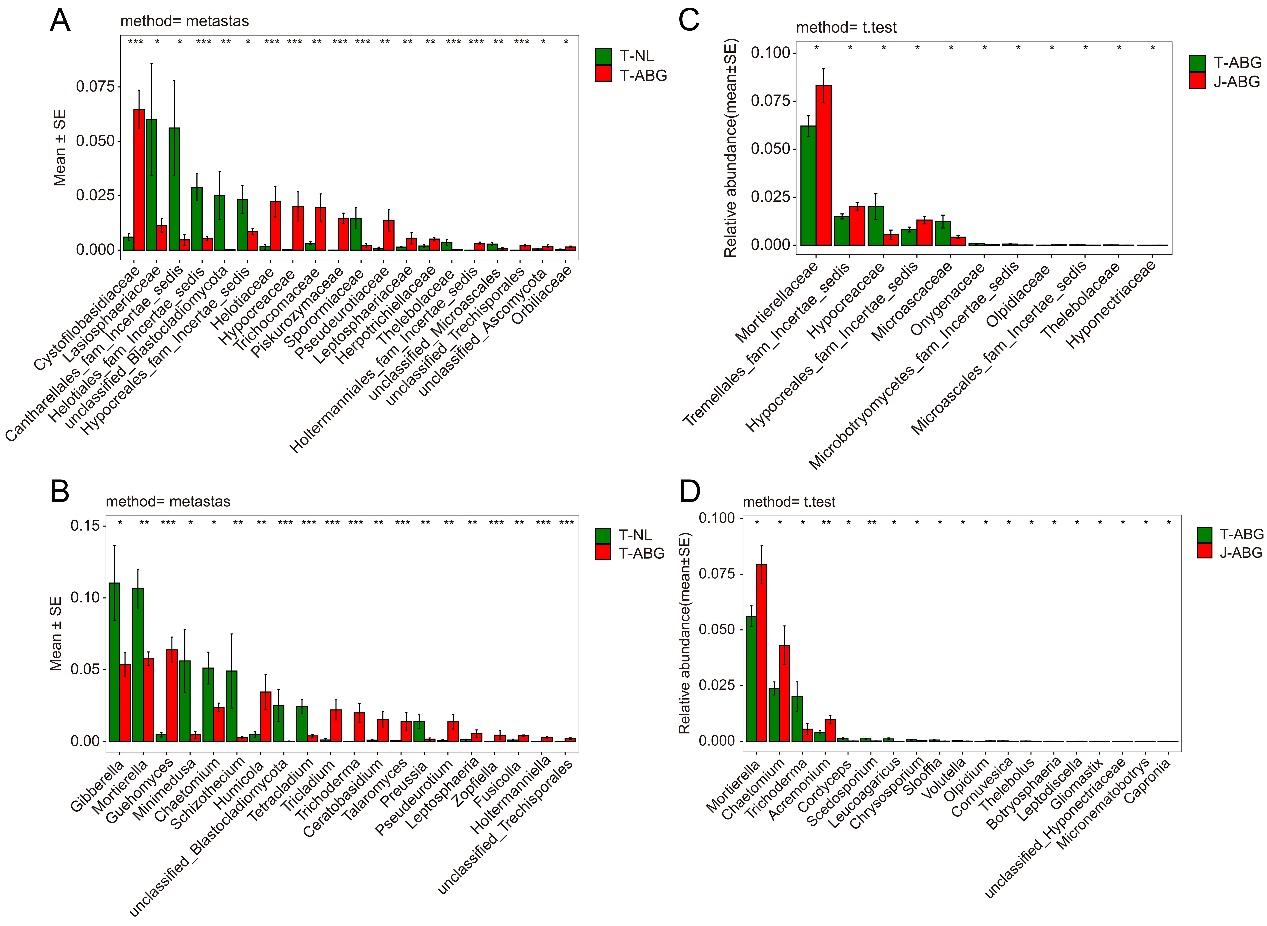


Supplementary Figure 15. Difference matrix heatmap (A) and non-metric multidimensional scaling (NMDS) for fungal communities in rhizospheric soil from diseased and healthy apple trees in the ABG, analyzed using the unweighted UniFrac method. B, D, F, H, J, L, N, P, R, T: Difference matrix heatmap for each orchard. C, E, G, I, K, M, O, Q, S, U: Non-metric multidimensional scaling (NMDS) for each orchard. Different colored dots represent different samples.


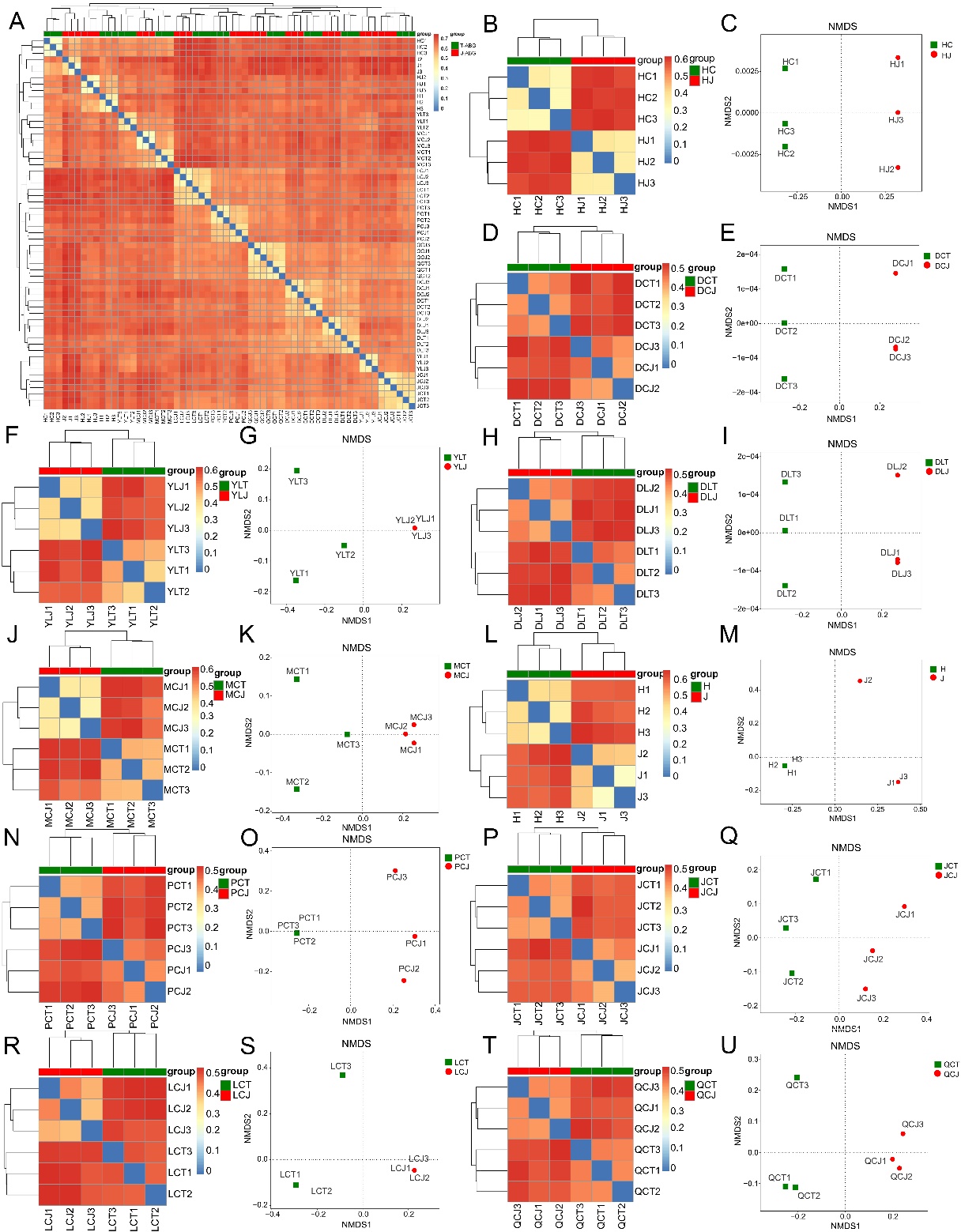


Supplementary Figure 16. Redundancy analysis (RDA) of abundant fungal genera and soil properties for NL (A) and ABG (B) samples from 16 sampling areas. Environmental factors are indicated by red arrows, and major genera are indicated by blue arrows. Soil properties include available phosphorus (AP), available potassium (AK), organic matter (SOM), soil bulk density (ρb), available nitrogen (AN), pH, soil moisture content (ω), and phloridin.


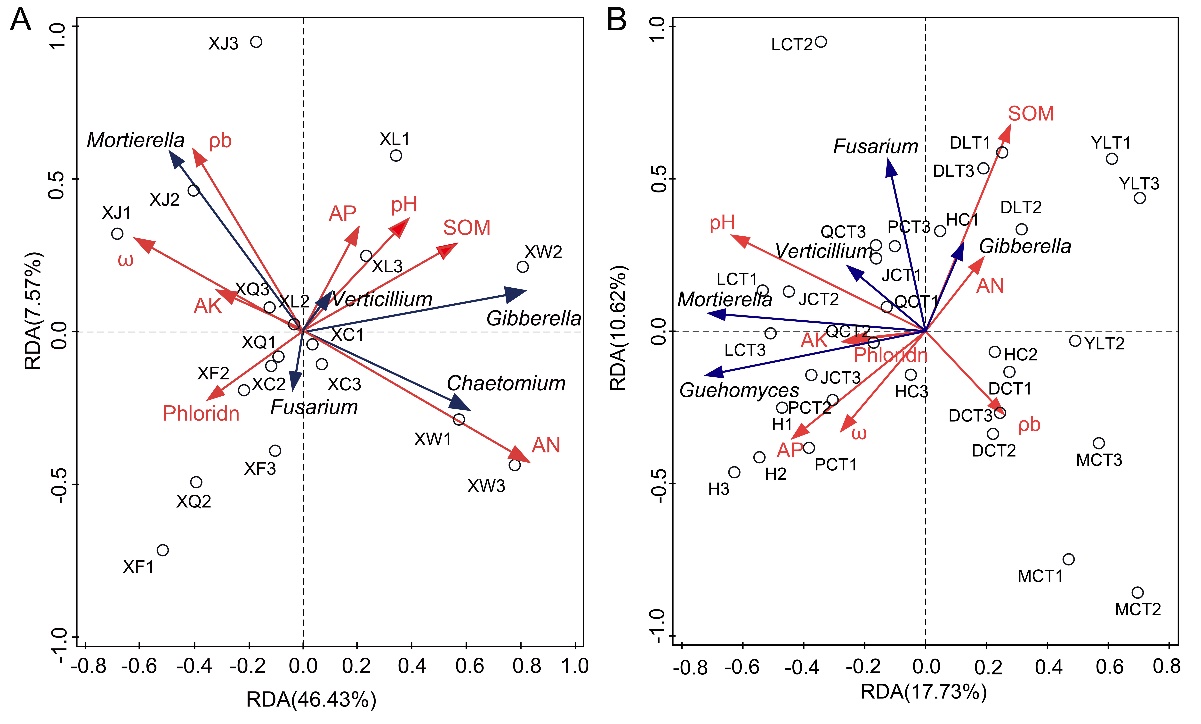


Supplementary Figure 17. Structural equation models (SEMs) of soil properties (ρb, AP, pH, AK, and SOM), the relative abundance of *Mortierella* and *Fusarium*, apple replant disease severity, cinnamic acid (Ca), *p*-hydroxybenzoic acid (Pha), phloridin (Pd), and syringate (St) (A). Structural equation models (SEMs) of soil phenolic acids (Ca, Pha, Pd, and St), the relative abundance of *Mortierella* and *Fusarium*, apple replant disease severity, soil bulk density (ρb), pH, available phosphorus (AP), available potassium (AK), and organic matter (SOM) (B). Arrows indicate significant relationships, dotted lines indicate non-significant relationships, and solid lines indicate significant effects. Blue lines represent positive effects, and red lines represent negative effects. Numbers above arrow lines are standardized path coefficients. *R²* indicates the proportion of variance explained by the model. ***P* < 0.01; **P* < 0.05.


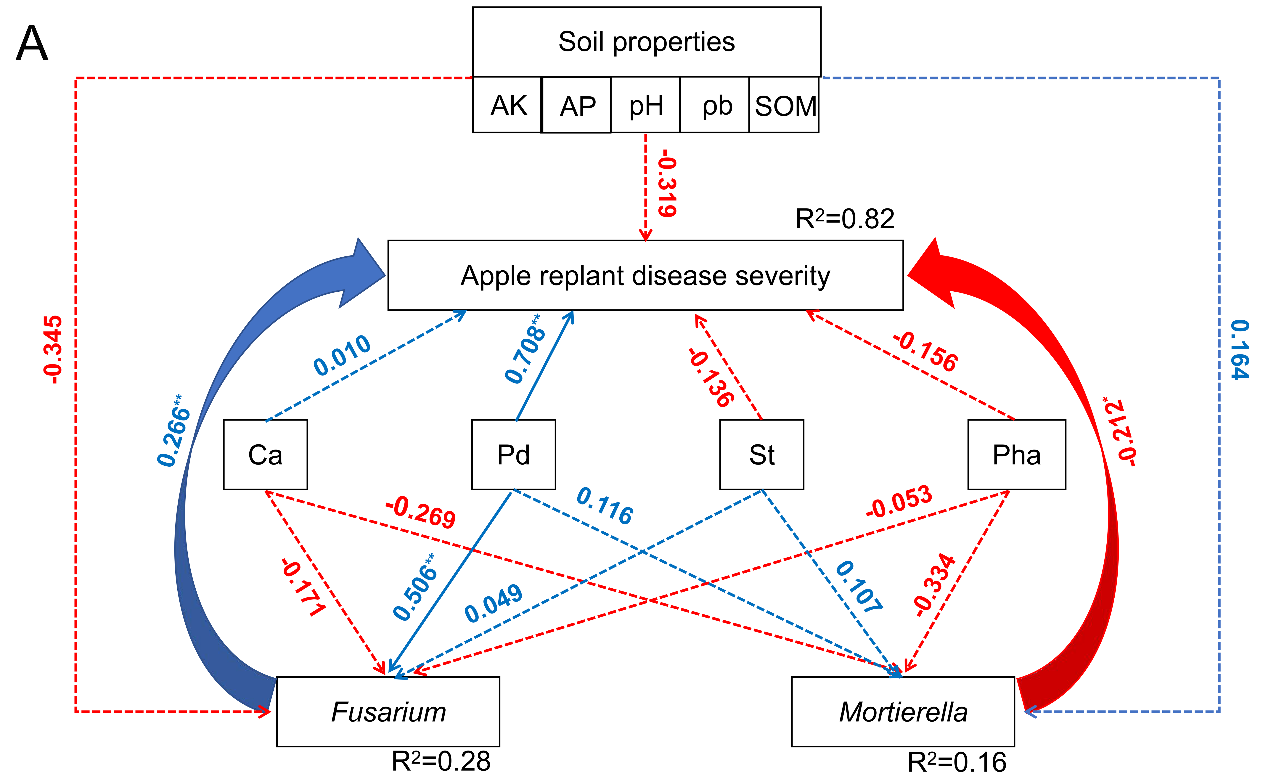


Supplementary Figure 18. A (*Malus hupehensis* Rehd. seedling) and B (M.9T337): Control treated with sterile distilled water. C-D: Pathogenicity and disease symptoms of M.9T337 (D) and *Malus hupehensis* Rehd. seedling (C) infected by strain HC139. E-N: Culture characteristics and microscopic appearance of strain HC139. E-G: *Phoma macrostoma* HC139 (isolated from soil in Penglai, China). E: Culture on corn-agar medium. F: Culture on potato-dextrose agar medium. G: Culture on oatmeal agar medium. H-L: Aerial mycelium. M-N: Conidia. Scale bars: 10 μm (M-N); 15 μm (H-L).


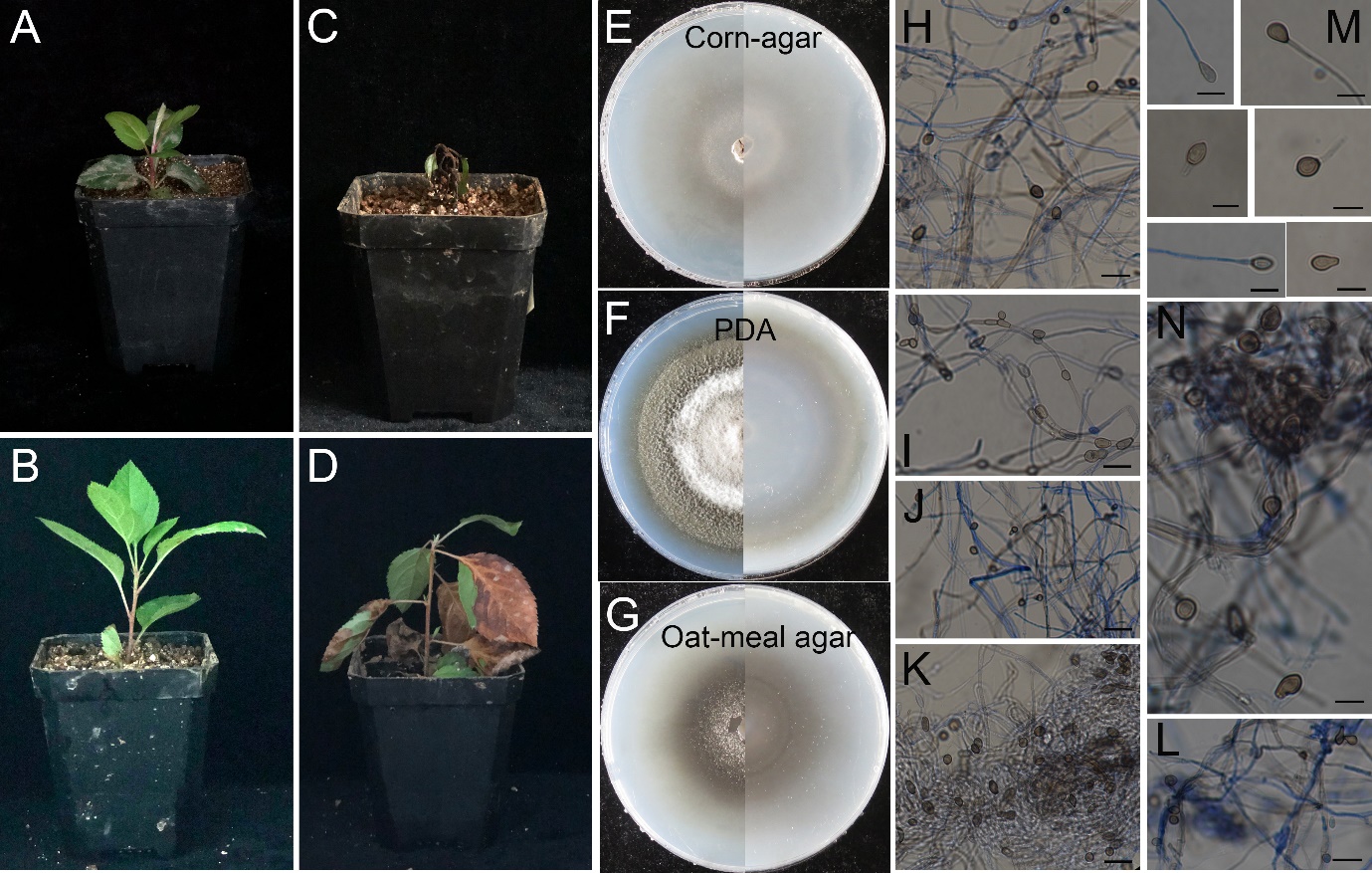


Supplementary Figure 19. The ML consensus tree inferred from the combined ITS, TEF, and TUB2 sequence alignment. Support values (ML bootstrap and posterior probability values) are indicated at the branches. Support for each branch in the inferred tree was evaluated using 1000 bootstrap replications. The scale bar indicates 0.06 expected changes per site. Clade numbers and Latin names are provided on the right of the tree and these are used for reference in the treatment of the species. The tree is rooted to *Peyronellaea eucalypti* ([CBS 142522](https://www.ncbi.nlm.nih.gov/nuccore/MH553546.1)). Strain HC131, YR15, Q61, and HC39 are indicated in bold and red


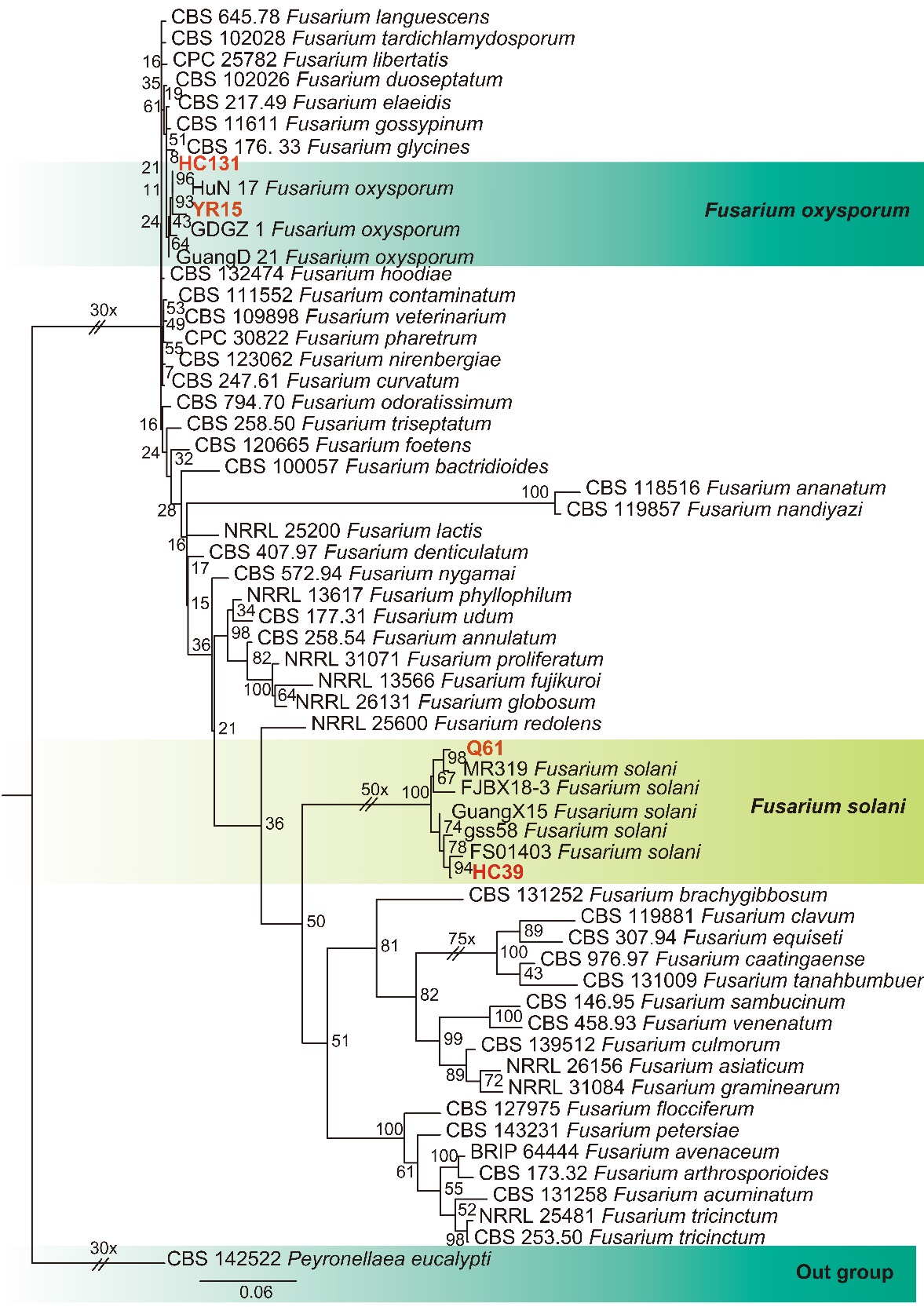


Supplementary Figure 20. The ML consensus tree inferred from the combined ITS and TUB2 sequence alignment. Support values (ML bootstrap and posterior probability values) are indicated at the branches. Support for each branch in the inferred tree was evaluated using 1000 bootstrap replications. The scale bar indicates 0.3 expected changes per site. Clade numbers and Latin names are provided on the right of the tree and these are used for reference in the treatment of the species. The tree is rooted to *Verrucoconiothyrium eucalyptigen* (CBS 142535). Strain HC139 is indicated in bold and red.


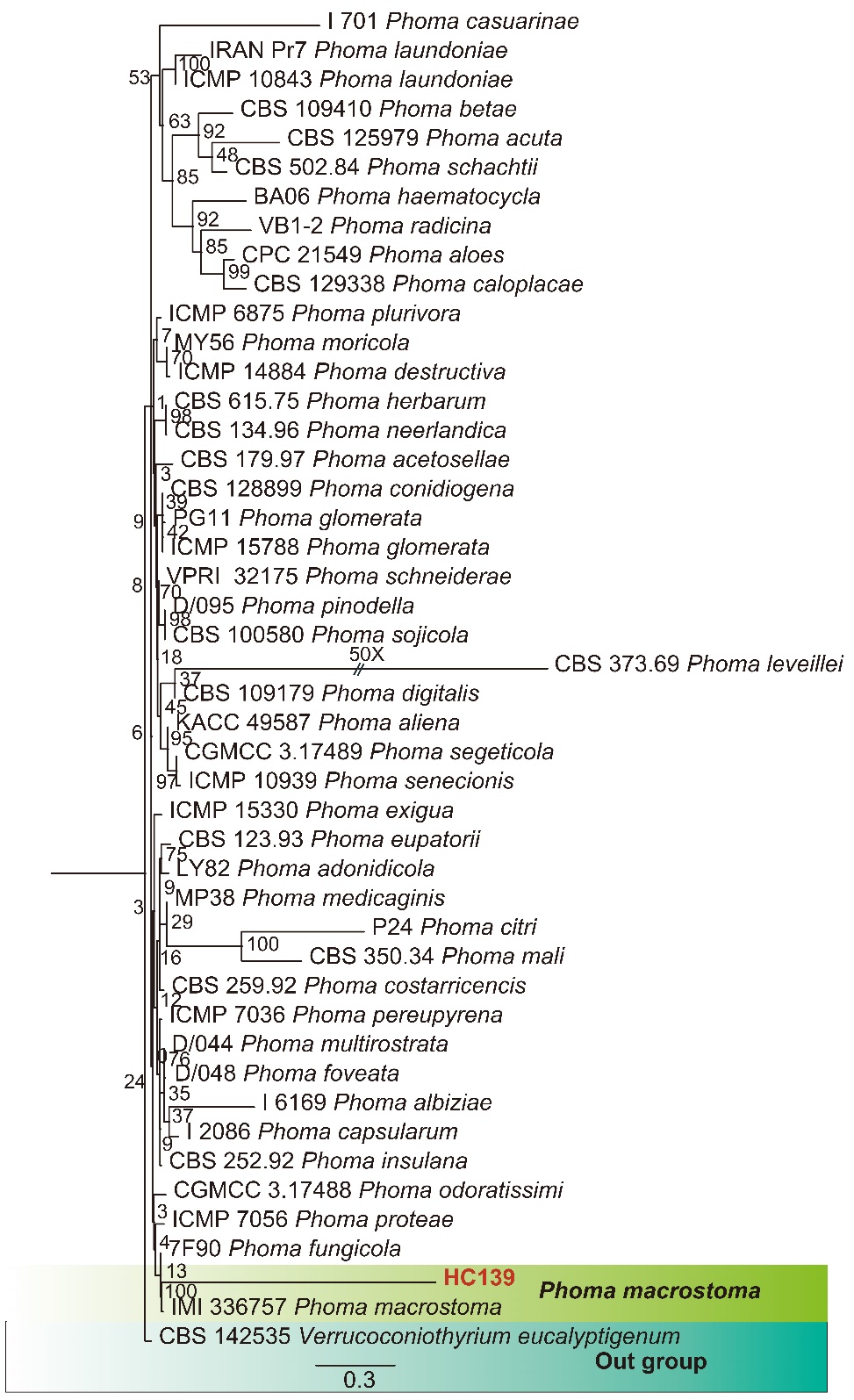


Supplementary Figure 21. The ML consensus tree inferred from the ITS sequence alignment. Support values (ML bootstrap and posterior probability values) are indicated at the branches. Support for each branch in the inferred tree was evaluated using 1000 bootstrap replications. The scale bar indicates 4.0 expected changes per site. Clade numbers and Latin names are provided on the right of the tree and these are used for reference in the treatment of the species. The tree is rooted to *Paradendryphiella salina* (= *E. annulata*) (CBS 302.84T). Names of sections and monotypic lineages (MTL) for each taxon are given in the right column. Strain YR9 is indicated in bold and red.


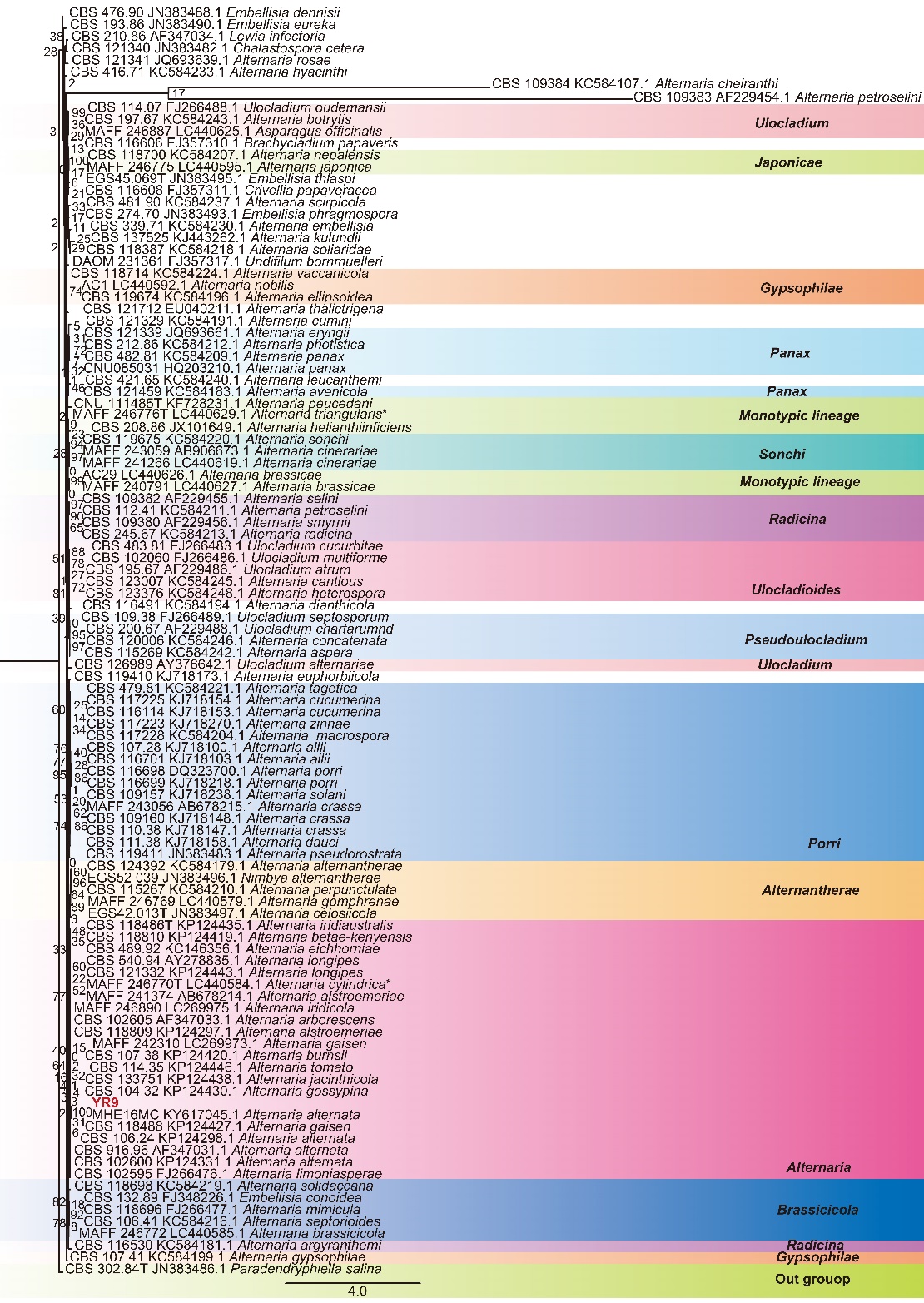


Supplementary Figure 22. The ML consensus tree inferred from the combined ITS and TUB2 sequence alignment. Support values (ML bootstrap and posterior probability values) are indicated at the branches. Support for each branch in the inferred tree was evaluated using 1000 bootstrap replications. The scale bar indicates 0.3 expected changes per site. Clade numbers and Latin names are provided on the right of the tree and these are used for reference in the treatment of the species. The tree is rooted to *Didymella glomerata* (CBS 284.76). Strain XW23 is indicated in bold and red.


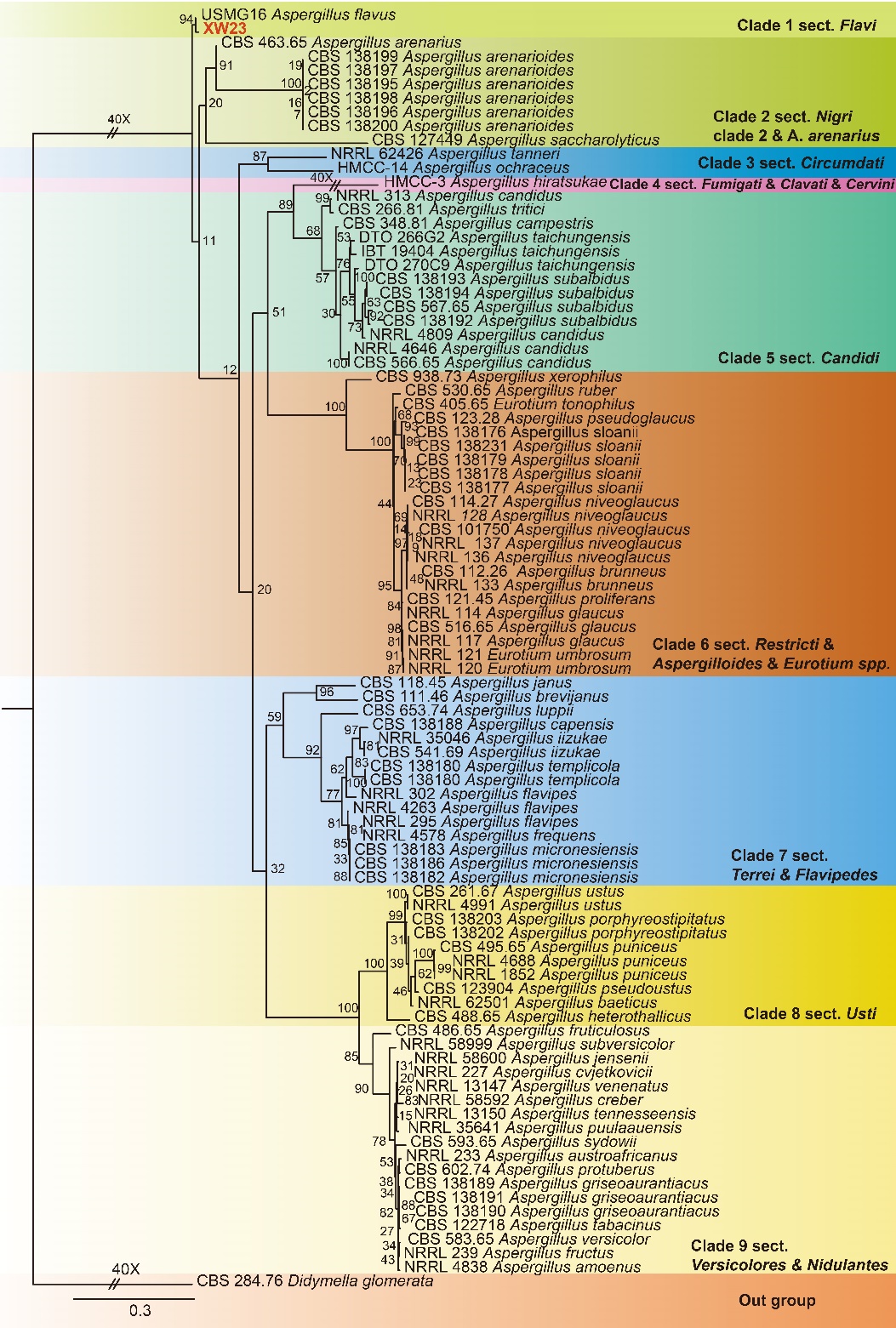


Supplementary Figure 23. The ML consensus tree inferred from the combined ITS and TUB2 sequence alignment. Support values (ML bootstrap and posterior probability values) are indicated at the branches. Support for each branch in the inferred tree was evaluated using 1000 bootstrap replications. The scale bar indicates 0.5 expected changes per site. Clade numbers and Latin names are provided on the right of the tree and these are used for reference in the treatment of the species. The tree is rooted to *Coniothyrium clematidis-rectae* (CBS 507.63). Strain Q9 is indicated in bold and red.


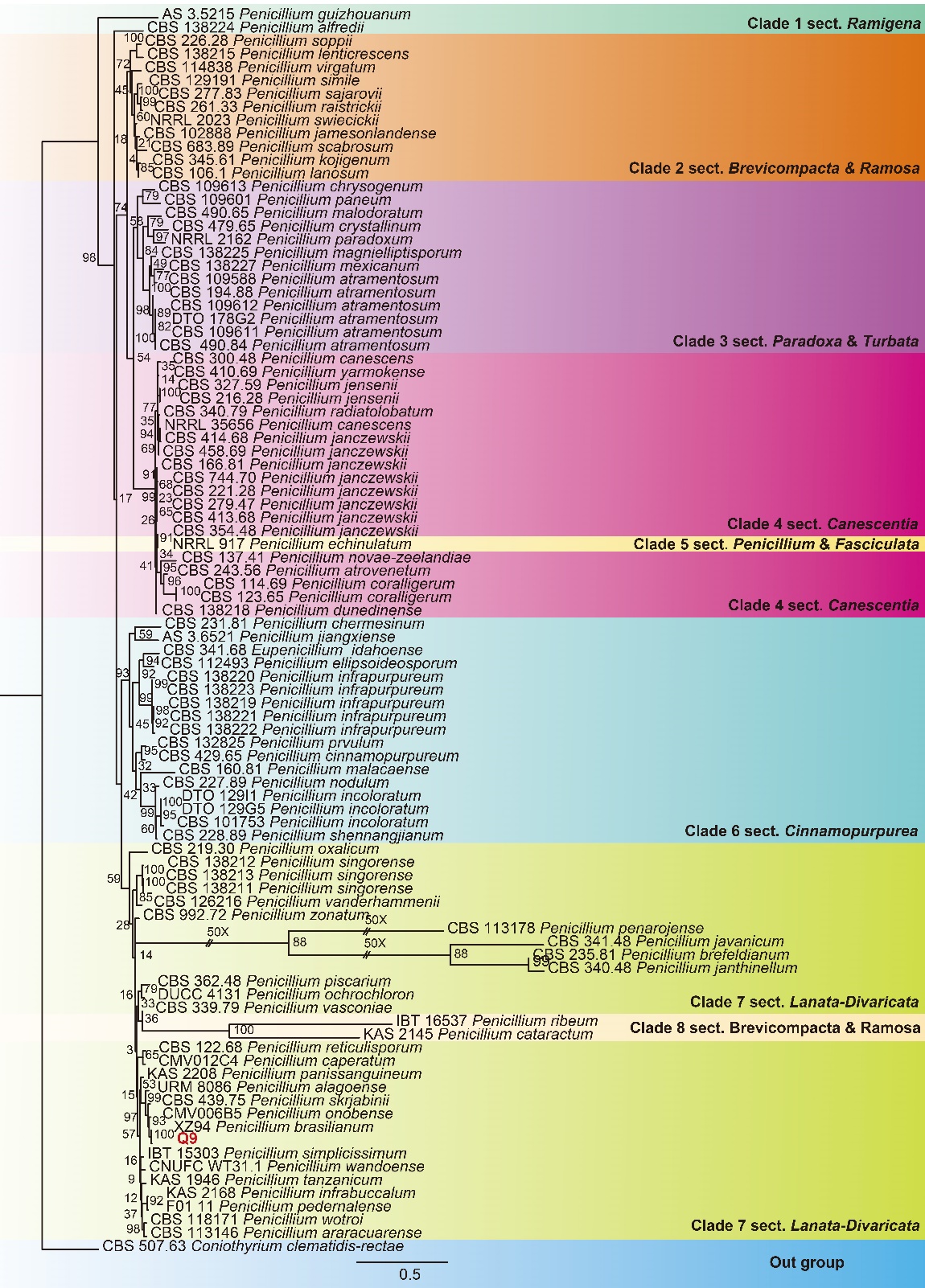


Supplementary Figure 24. The ML consensus tree inferred from the combined ITS and TUB2 sequence alignment. Support values (ML bootstrap and posterior probability values) are indicated at the branches. Support for each branch in the inferred tree was evaluated using 1000 bootstrap replications. The scale bar indicates 0.07 expected changes per site. Clade numbers and Latin names are provided on the right of the tree and these are used for reference in the treatment of the species. The tree is rooted to *Septomyrothecium maraitiense* (MUCL 47202). Strain XW39 is indicated in bold and red.


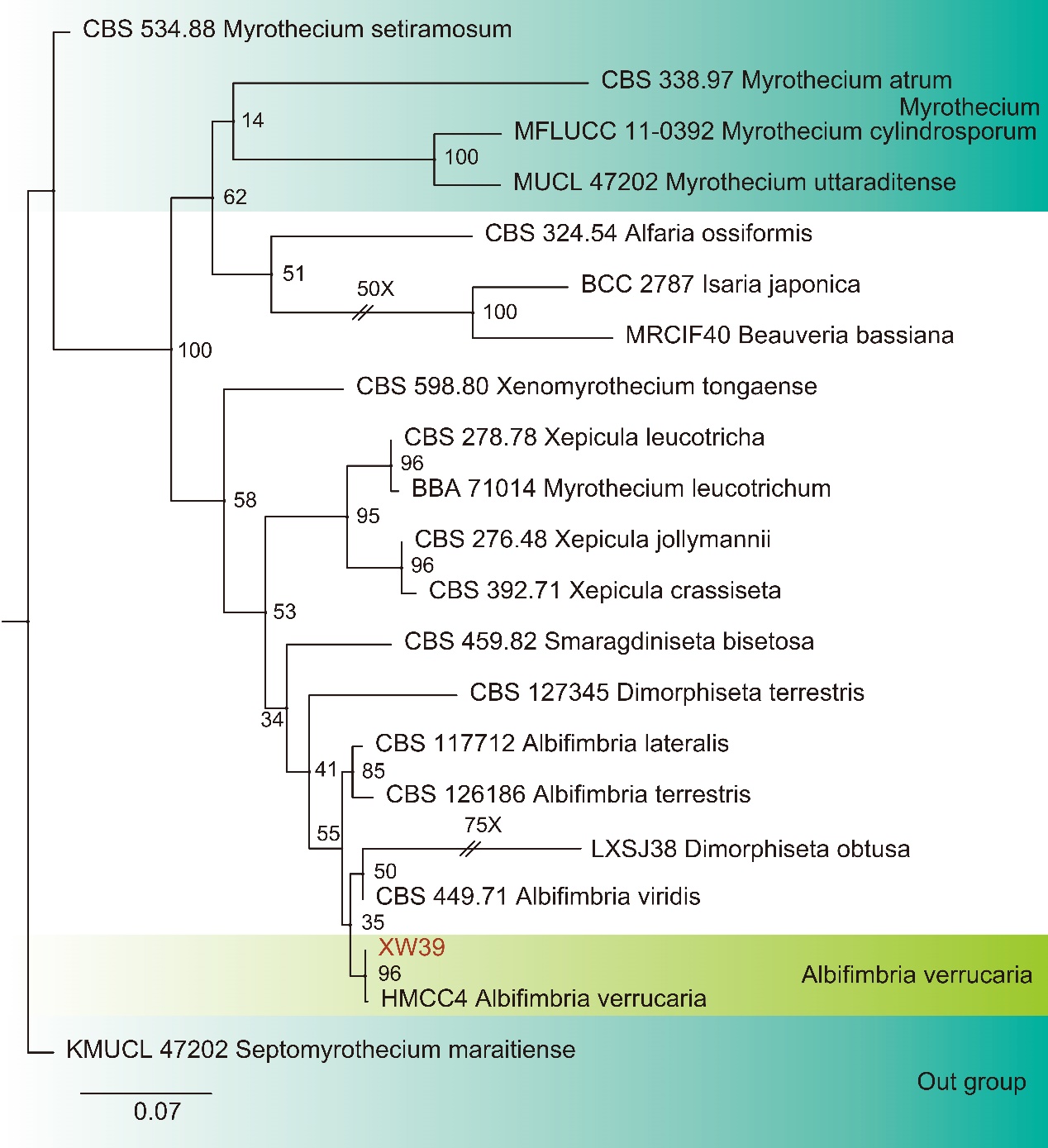


Supplementary Figure 25. Determination of the ability of strain MR5 to produce extracellular enzyme activity. A-B: Plate assay. C-D: Colorimetry. A, C: Polygalacturonases (PGAse) activity. B, D: Endo-1,4-β-glucanase activity (CMCellulase).


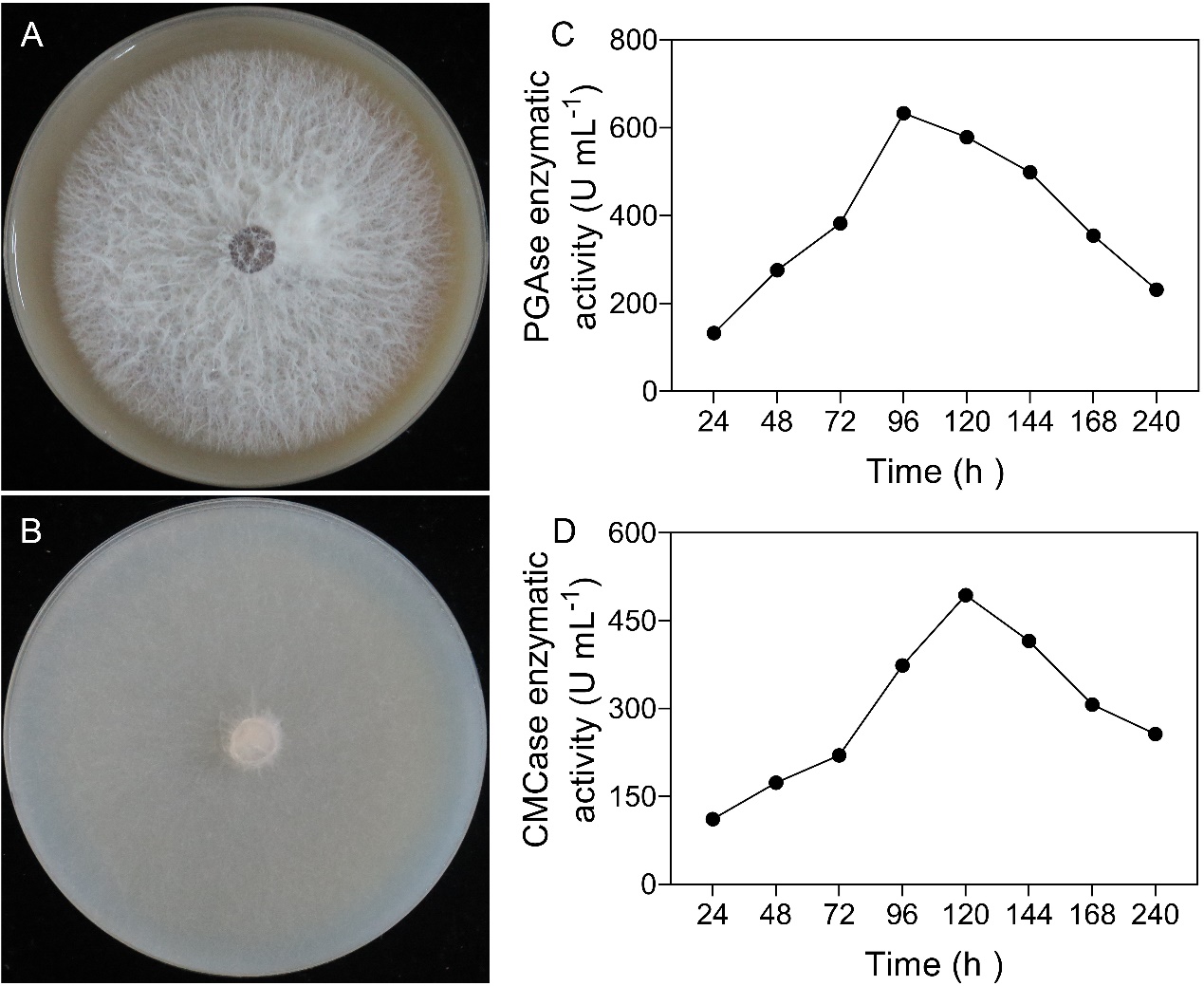


Spplementary Figure 26. Effects of 11 fungicides on conidia germination of *Fusarium proliferatum* f.sp. *malus domestica*.


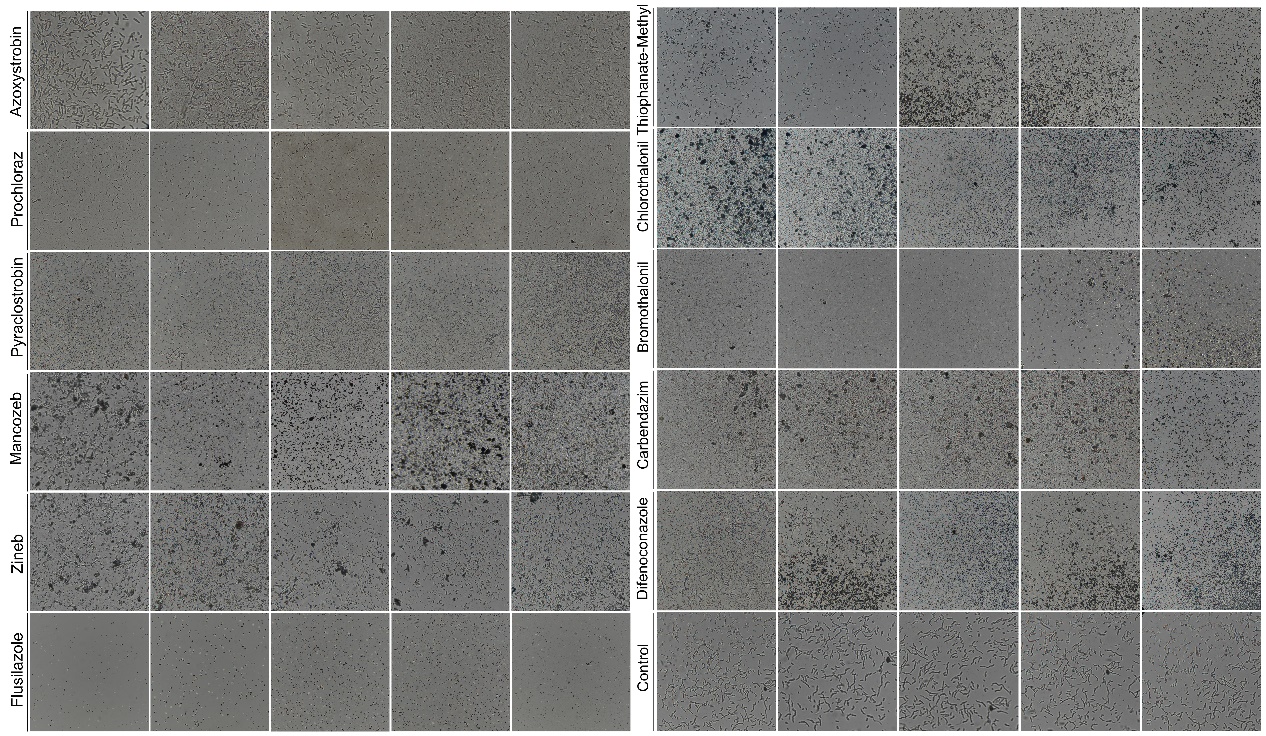

Supplement: Supplementary file 1 — Supplementary Material 1. [file 44154_2025_258_MOESM1_ESM.docx]
