# Supplementary material for: Apple replant disease: unraveling the fungal enigma hidden in the rhizosphere
Source: Stress Biol. 2025 Nov 27;5(1):71. doi: 10.1007/s44154-025-00258-1 (PMC12657686; doi:10.1007/s44154-025-00258-1)
Supplement: Supplementary file 2 — Supplementary Material 2. [file 44154_2025_258_MOESM2_ESM.docx]

Supplementary materials

Supplementary Table 1. Basic information of the sampled orchards.

| Sampling location | Address | Location numbera | Latitude and longitude | Construction situation | Row spacing | Rootstock | Soil texture |
| --- | --- | --- | --- | --- | --- | --- | --- |
| Around Bohai Gulf | Guanshui Town, Muping City, Shandong Province | MCT | Lon: 121.30903 | Over 20 years old orchard established for 5 years | 4 × 1 | *Malus* × robusta (CarriŠre) Rehder | Sandy loam |
|  |  | MCJ | Lat: 37.17033 |  |  |  |  |
|  | Liujiagou Town, Penglai City, Shandong Province | PCT | Lon: 120.883503 | Over 20 years old orchard established for 3 years | 3 × 4 | *Malus hupehensis* Rehd. | Sandy loam |
|  |  | PCJ | Lat: 37.741059 |  |  |  |  |
|  | Wan Tou Village, Laizhou City, Shandong Province | LCT | Lon: 119.814701 | Over 20 years old orchard established for 3 years | 4 × 1.5 | *Malus* × robusta (CarriŠre) Rehder | Sandy loam |
|  |  | LCJ | Lat: 37.095159 |  |  |  |  |
|  | Guanli Town, Qixia City, Shandong Province | QCT | Lon: 120.73525 | Over 20 years old orchard established for 4 years | 4 × 4 | *Malus* × robusta (CarriŠre) Rehder | Sandy loam |
|  |  | QCJ | Lat: 37.16557 |  |  |  |  |
|  | Yiyuan Town, Zibo City, Shandong Province | YLT | Lon: 118.434563 | Over 20 years old orchard | 3 × 3 | *Malus baccata* (Linn.) Borkh. | Sandy loam |
|  |  | YLJ | Lat: 36.085704 |  |  |  |  |
|  | Xiongyue Town, Dalian City, Liaoning Province | DLT | Lon: 122.162251 | Over 25 years old orchard | 5 × 5 | *Malus baccata* (Linn.) Borkh. | Sandy loam |
|  |  | DLJ | Lat: 40.181132 |  |  |  |  |
|  |  | DCT | Lon: 122.162251 | Over 20 years old orchard established for 5 years | 3 × 4 | *Malus hupehensis* Rehd. | Sandy loam |
|  |  | DCJ | Lat: 40.181132 |  |  |  |  |
|  | Jinzhou District, Dalian City, Liaoning Province | JCT | Lon: 121.76626 | Over 20 years old orchard established for 5 years | 4 × 1 | M.9T337 | Sandy loam |
|  |  | JCJ | Lat: 39.25402 |  |  |  |  |
|  | Suizhong County, Huludao City, Liaoning Province | HC | Lon: 119.89260 | Over 25 years old orchard established for 3 years | 4 × 1 | *Malus* × robusta (CarriŠre) Rehder | Sandy loam |
|  |  | HJ | Lat: 40.03144 |  |  |  |  |
|  | Xingcheng County, Huludao City, Liaoning Province | H | Lon: 120.72937 | Over 20 years old orchard established for 5 years | 4 × 1 | Rootstock: Malus × robusta (CarriŠre) Rehder | Sandy loam |
|  |  | J | Lat: 40.61941 |  |  |  |  |
| Northwest Loess region | Qianxian County, Xianyang City, Shaanxi Province | XQT | Lon: 108.24741 | Over 25 years old orchard established for 3 years | 4 × 1 | *Malus baccata* (Linn.) Borkh. | Silty clay loam |
|  |  | XQJ | Lat: 34.52726 |  |  |  |  |
|  | Chunyu County, Xianyang City, Shaanxi Province | XCT | Lon: 108.58117 | Over 25 years old orchard established for 3 years | 4 × 1 | *Malus baccata* (Linn.) Borkh. | Silty clay loam |
|  |  | XCJ | Lat: 34.79797 |  |  |  |  |
|  | Jingning County, Pingliang, Gansu Province | XJT | Lon: 105.73349 | Over 25 years old orchard established for 3 years | 4 × 1 | *Malus baccata* (Linn.) Borkh. | Silty clay loam |
|  |  | XJJ | Lat: 35.52524 |  |  |  |  |
|  | Luochuan County, Yan'an City, Shaanxi Province | XLT | Lon: 109.43571 | Over 26 years old orchard established for 4years | 4 × 1 | *Malus baccata* (Linn.) Borkh. | Sandy loam |
|  |  | XLJ | Lat: 35.76213 |  |  |  |  |
|  | [Fu County, Yan'an City, Shaanxi Province](http://map.sogou.com/) | XFT | Lon: 109.38414 | Over 25 years old orchard established for 4 years | 4 × 1 | *Malus baccata* (Linn.) Borkh. | Sandy loam |
|  |  | XFJ | Lat: 35.99650 |  |  |  |  |
|  | Wanrong County, Yuncheng City, Shanxi Province | XWT | Lon: 110.84356 | Over 26 years old orchard established for 5 years | 4 × 1 | *Malus baccata* (Linn.) Borkh. | Sandy loam |
|  |  | XWJ | Lat: 35.41704 |  |  |  |  |

^a^Numbering rules for the Around Bohai Gulf Planting Area: The first letter is the phonetic alphabet of the region, the second letter "C" represents the repeated orchard, "L" represents the old apple orchard, the last letter "T" represents the rhizosphere soil of the weak/dead trees and "J" represents the rhizosphere soil of the healthy trees. Numbering rules for the Loess Plateau Planting Area: The first letter represents the Loess Plateau Planting Area, the second letter is the phonetic alphabet of the region, the last letter as above.

Supplementary Table 2. The culture medium used for fungal isolation in this study, as well as its preparation method and function.

| Culture medium | Composition^a^ | Function |
| --- | --- | --- |
| Potato dextrose agar (PDA) | Potato 200 g, Dextrose 20 g, Agar 16 g. Add ampicillin and streptomyc in 50 mg·L^-1^ each. | Isolation and culture of Fungus. |
| Potato sucrose medium | Potato 200 g, Sucrose 20 g, Agar 16 g. Add ampicillin and streptomyc in 50 mg·L^-1^ each. | Isolation and culture of Fungus. |
| V8 medium | U.S. imports V8 vegetable juice 200 g, CaCO_3_ 2 g, Agar 16 g. | Isolation and culture of *Phytophthora* and induction of oospores. The formation of the sexual stage of many *Fusarium* species. |
| Carrot agar medium (CA) | Carrot 200 g, Agar 16 g. | Isolation and culture of *Phytophthora* and induction of oospores. |
| Oats agar medium (OA) | Oats 60 g, Agar 16 g. | Isolation and culture of *Pythium* and induction of oospores. |
| Water agar (WA) | Agar 20 g. | Induction of germinating conidia of *Fusarium* and isolation of *Fusarium* species from plant material, particularly roots. |
| Corn Meal Agar (CMA) | Corn 200 g, Agar 20 g. | Isolation of *Pythium* and *Phytophthora*. |
| Richard medium | KNO_3_ 10 g, KH_2_PO_4_ 5 g, MgSO_4_·7H_2_O 2.5 g, FeCl_3_ 0.02 g, Sucrose 50 g, Agar 16 g. | Isolation of *Rhizoctonia*. |
| Pimaricin + ampicillin + rifampicin agar (PARP) medium | CMA + pimaricin 5 mg·L^-1^ + ampicillin 50 mg·L^-1^ + rifampicin 10 mg·L^-1^. | Isolation of *Pythium* and *Phytophthora*. |
| Saltwater Nutrient Agar (SNA) Medium | KNO_3_ 1 g, KH_2_PO_4_ 1 g, MgSO_4_·7H_2_O 0.5 g, KCl 0.5 g, Sucrose 0.2 g, Dextrose 0.2 g, Agar 20 g. | The identification of *Fusarium*. |
| KCL Carnation Leaf-Piece Agar (KCLA) | Sterile carnation leaf pieces, KCl 8 g, Agar 16 g. | Inducing *Fusarium* to produce Macroconidia and Microconidia. When KCl is added to CLA the fungal cultures produce more and longer microconidial chains. |
| Czapek's medium | NaNO_3_ 3 g, K_2_HPO_4_ 1 g, MgSO_4_·7H_2_O 0.5 g, KCl 0.5 g, FeSO_4_ 0.01 g, Sucrose 30 g, Agar 16 g. | Identification and Conservation of *Penicillium* and *Aspergillus.* |

^a^Transferred to an Erlenmeyer flask containing 1 L of deionized water according to the composition. After gentle stirring, the mixture was heated in a microwave oven until complete solubilized. Adjust the pH to 7.0 - 7.2 and autoclave it for later use. The solution was cooled at room temperature until the temperature dropped to 40-50°C, and then an antibiotic was added and dissolved homogeneously.

Supplementary Table 3. Morphological identification and phylogenetic analysis methods of isolates.

| Species | | Identification method | Morphological observation | Culture characteristics | Phylogenetic analyses |
| --- | --- | --- | --- | --- | --- |
| *Fusarium* | *F. oxysporum* (HC131, YR15) | *Fusarium* was identified based on the *Fusarium* laboratory manual (Leslie and Summerell 2006). | For microscopic observations, the isolates were newly plated onto Carnation Leaf Agar (CLA) and/or Spezieller Nahrst-offarmer agar (SNA). Plates were maintained at 25°C in the dark for 7-14 d. | The isolate was plated onto potato dextrose agar (PDA: 40 g potato, 20 g dextrose, 20 g agar in 1.0 L distilled water) plates. After incubation in the dark for 7 d at 25°C. | The maximum likelihood (ML) analysis of identities was carried out based on ITS, TEF, and TUB2 sequences. |
|  | 1. *solani* (Q61, HC39) |  |  |  |  |
|  | *F. proliferatum* (MR5) |  |  |  | The maximum likelihood (ML) analysis of identities was carried out based on ITS, mtSSU, TEF, TUB2, RPB2, and IGS sequences. |
| *Alternaria* | *Alternaria alternata* (YR9) | For microscopic observations of the diagnostic morphology comparable to those of Simmons’s standard conditions (2007), sporulation was induced according to methods reported by Nishikawa and Nakashima (2013). | For microscopic observations, the isolates were newly plated onto V8 juice agar (V8) and/or potato carrot agar (PCA). Plates were maintained at 25°C in the dark for 7-14 d. | The isolate was plated onto potato dextrose agar (PDA: 40 g potato, 20 g dextrose, 20 g agar in 1.0 L distilled water) plates. After incubation in the dark for 7 d at 25°C. | The maximum likelihood (ML) analysis of identities was conducted based on ITS sequence. |
| *Aspergillus* | *Aspergillus flavus* XW23 | *Aspergillus* species were identified using the Manual of *Aspergillus* Identification (Klich 2002; Diba et al. 2007; Thathana et al. 2017). | For microscopic observations, the isolates were newly plated onto czapek dox agar (CZ), czapek yeast agar (CYA), malt extract agar (MEA), and czapek yeast 20% sucrose agar, and the plates were maintained at 25°C in the dark for 7-14 d. | The isolate was plated onto potato dextrose agar (PDA: 40 g potato, 20 g dextrose, 20 g agar in 1.0 L distilled water) plates. After incubation in the dark for 7 d at 25°C. | The maximum likelihood (ML) analysis of identities was conducted based on ITS and TUB2 sequences. |
| *Penicillium* | *Penicilliumbrasilianum* Q9 | *Penicillium* species were identified based on the standardised methods for laboratories identifying and describing *Penicillium* species (Visagie et al. 2014). | For microscopic observations, the isolates were newly plated onto malt extract agar (MEA), and czapek yeast 20% sucrose agar, and the plates were maintained at 25°C in the dark for 7-14 d. | The isolate was plated onto czapek yeast agar (CYA), malt extract agar (MEA), potato dextrose agar (PDA: 40 g potato, 20 g dextrose, 20 g agar in 1.0 L distilled water) plates. After incubation in the dark for 7 d at 25°C (Sang et al. 2014). | The maximum likelihood (ML) analysis of identities was conducted based on ITS and TUB2 sequences. |
| *Albifimbria* | *Albifimbria verrucaria* XW39 | For microscopic observations comparable to those of Domsch and Lombard’s standard conditions (1980; 2016). | For microscopic observations, the isolates were newly plated onto PDA, OA, and CMA, and the plates were maintained at 25°C in the dark for 7-14 d. | The isolate was plated onto potato dextrose agar (PDA: 40 g potato, 20 g dextrose, 20 g agar in 1.0 L distilled water) plates. After incubation in the dark for 7 d at 25°C. | The maximum likelihood (ML) analysis of identities was conducted based on ITS and TUB2 sequences. |
| *Phoma* | *Phoma macrostoma* HC139 | *Phoma* was identified based on the *Phoma* identification manual (Boerema et al. 2004). | For microscopic observations, the isolates were newly plated onto malt extract agar (MEA), and oat-meal agar, and the plates were maintained at 25°C in the dark for 7-14 d. | The isolate was plated onto corn-agar, oat-meal agar, and potato dextrose agar (PDA; 40 g potato, 20 g dextrose, 20 g agar in 1.0 L distilled water) plates. After incubation in the dark for 7 d at 25°C (Kukhar et al. 2020). | The maximum likelihood (ML) analysis of identities was conducted based on ITS and TUB2 sequences. |

Supplementary Table 4. Oligonucleotide primers and probes used for the quantitative real-time PCR detection of species.

| Locus^1^ | Primer name | Oligonucleotide sequence (5′-3′) | PCR cycles |
| --- | --- | --- | --- |
| ITS | ITS1/ITS4 | TCCGTAGGTGAACCTGCGC  TCCTCCGCTTATTGATATGC | Lombard et el. 2015; White et al. 1990 |
| TEF1-α | EF1/EF2 | ATGGGTAAGGARGACAAGAC  GGARGTACCAGTSATCATGTT | Mohd 2021; O’Donnell et el. 1998 |
| TUB2 | BT2A/BT2B | GGTAACCAAATCGGTGCTGCTTTC  ACCCTCAGTGTAGTGACCCTTGGC | Crous et al. 2021 |
| 18S gene | 18sF/18sR | GTAGTCATATGCTTGTCTC  TCCGCAGGTTCACCTACGGA | Liang et el. 2009 |
| IGS | CNS1/CNL12 | GAGACAAGCATATGACTACTG  CTGAACGCCTCTAAGTCAG | Mbofung et el. 2007 |
| mtSSU rDNA | NMS1/NMS2 | CAGCAGTGAGGAATATTGGTCAATG  GCGGATCATCGAATTAAATAACAT | Li et el. 1994 |
| RPB2 | RPB2-F/RPB2-R | GGGGWGAYCAGAAGAAGGC  CCCATRGCTTGYTTRCCCAT | Liu et el. 1999 |

^1^ITS: internal transcribed space, TEF1-α: translation elongation factor 1-alpha, TUB2: β-tubulin, IGS: intergenic spacer region of the rDNA, mtSSU rDNA: mitochondrial small subunit ribosomal DNA, RPB2: RNA polymerase II second largest subunit.

Supplementary Table 5. Related reference strains of *Fusarium* species used for phylogenetic analysis.

| Species | Culture accession^1^ | GenBank accession | | | Host/substrate | Origin |
| --- | --- | --- | --- | --- | --- | --- |
|  |  | TEF1 | ITS | TUB2 |  |  |
| *Fusarium curvatum* | CBS 247.61 = BBA 8398 = DSM 62308 = NRRL 22545 | MH484967.1 | n.a. | n.a. | *Matthiola incana* | Germany |
| *Fusarium duoseptatum* | CBS 102026 = NRRL 36115 | MH484987.1 | n.a. | MH485078.1 | *Musa sapientum* cv. *Pisang ambon* | Malaysia |
| *Fusarium elaeidis* | CBS 217.49 = NRRL 36358 | MH484961.1 | n.a. | MH485052.1 | *Elaeis* sp. | Zaire |
| *Fusarium foetens* | CBS 120665 | MH485009.1 | n.a. | MH485100.1 | *Nicotiana tabacum* | Iran |
| *Fusarium glycines* | CBS 176.33 = NRRL 36286 | MH484959.1 | n.a. | MH485050.1 | *Linum usitatissium* | Unknown |
| *Fusarium gossypinum* | CBS 116611 | MH484998.1 | n.a. | MH485089.1 | *Gossypium hirsutum* | Ivory Coast |
| *Fusarium hoodiae* | CBS 132474 | MH485020.1 | n.a. | MH485111.1 | *Hoodia gordonii* | South Africa |
| *Fusarium languescens* | CBS 645.78 = NRRL 36531 | MH484971.1 | n.a. | MH485062.1 | *Solanum lycopersicum* | Morocco |
| *Fusarium libertatis* | CBS 144748 = CPC 25782 | MH485023.1 | n.a. | MH485114.1 | *Aspalathus* sp. | South Africa |
| *Fusarium odoratissimum* | CBS 794.70 = BBA 11103 = NRRL 22550 | MH484969.1 | n.a. | MH485060.1 | *Albizzia julibrissin* | Iran |
| *Fusarium pharetrum* | CBS 144750 = CPC 30822 | MH485042.1 | n.a. | MH485133.1 | *Aliodendron dichotomum* | South Africa |
| *Fusarium trachichlamydosporum* | CBS 102028 = NRRL 36117 | MH484988.1 | n.a. | MH485079.1 | *M. sapientum* cv. *Pisang awak legor* | Malaysia |
| *Fusarium triseptatum* | CBS 258.50 = NRRL 36389T | MH484964.1 | n.a. | MH485055.1 | *Ipomoea batatas* | USA |
| *Fusarium udum* | CBS 177.31 | MH484957.1 | n.a. | MH485048.1 | *Digitaria eriantha* | South Africa |
| *Fusarium veterinarium* | CBS 109898 = NRRL 36153T | MH484990.1 | n.a. | MH485081.1 | *Shark peritoneum* | The Netherlands |
| *Fusarium equiseti* | CBS 307.94 | KR071777.1 | MH862468.1 | n.a. | culture from neotype of *Fusarium equiseti* | Germany |
| *Fusarium culmorum* | CBS 139512 | KT855186.1 | n.a. | n.a. | Unknown | Unknown |
| *Fusarium graminearum* | CBS 123657 = NRRL 31084 | HM744693.1 | n.a. | n.a. | Unknown | northern Asia |
| *Fusarium asiaticum* | NRRL 26156 = AS2 | AF212452.1 | NR_121320.1 | AF107856.1 | Culture from type material of *Fusarium asiaticum* | USA |
| *Fusarium tricinctum* | NRRL 25481 = ATCC 38183 | MH582379.1 | HM068317.1 | AB587077.1 | Culture from epitype of *Selenosporium tricinctum* | USA |
| *Fusarium acuminatum* | CBS 131258 = NRRL 54218 | JQ429337.1 | MH865933.1 | n.a. | Unknown | Iran |
| *Fusarium sambucinum* | CBS 146.95 | KM231941.1 | KM231813.1 | KM232078.1 | *Solanum tuberosum* | United Kingdom |
| *Fusarium venenatum* | CBS 458.93 | KM231942.1 | KM231814.1 | KM232079.1 | inter wheat | Austria |
| *Fusarium brachygibbosum* | CBS 131252 = SUF160 | JX118981.1 | JX162372.1 | n.a. | Unknown | Unknown |
| *Fusarium clavum* | CBS 119881 = NRRL 34037 = Feq 2 | MN170457.1 | GQ505727.1 | MN078862.1 | Unknown | Africa (Namibia) |
| *Fusarium tanahbumbuense* | CBS 131009 = NRRL 43297 | MN170506.1 | MW534656.1 | n.a. | *Triticum sp.* | Iran |
| *Fusarium caatingaense* | CBS 976.97 = URM 6777 | MN170449.1 | MH668814.1 | n.a. | *Juniperus chinensis* (Cupressaceae). | USA |
| *Fusarium tricinctum* | CBS 253.50 = NRRL 25481 = MAFF 235551 = JZ2-5 | KR071775.1 | MH856607.1 | AB587079.1 | Unknown | Finland |
| *Fusarium redolens* | NRRL 25600 = NRRL28181 = PUF026 | MT409453.1 | MT435064.1 | AY329040.1 | Unknown | Unknown |
| *Fusarium nygamai* | CBS 572.94 = PUF025 | MK639065.1 | HQ165928.1 | U34426.1 | Unknown | China |
| *Fusarium nirenbergiae* | CBS 123062 = An7 = FU13 | MH485010.1 | MW663997.1 | MH485101.1 | ulip roots | USA |
| *Fusarium avenaceum* | [BRIP 64444 = PUF034](http://www.biobw.org/China-strain/bio-74148.html) | KU529164.1 | KU529155.1 | n.a. | Unknown | China |
| *Fusarium arthrosporioides* | CBS 173.32 = F-2302 = NRRL 26416 = BBA64327 | KU304441.1 | MH855264.1 | KU304454.1 | Unknown | Denmark |
| *Fusarium flocciferum* | CBS 127975 = NRRL 54147 | MH582390.1 | MH864782.1 | U85570.1 | Unknown | Unknown |
| *Fusarium petersiae* | CBS 143231 | MG386160.1 | MG386078.2 | n.a. | Garden soil | Netherlands |
| *Fusarium ananatum* | CBS 118516 = CBS 130392 | KU604416.1 | KU604024.1 | MN534089.1 | type material of *Fusarium ananatum* | Netherlands |
| *Fusarium denticulatum* | CBS 407.97 | MN534000.1 | NR_138359.1 | MN534067.1 | *Ipomoea batatas* | USA |
| *Fusarium fujikuroi* | NRRL 13566 | AF160279.1 | n.a. | n.a. | Unknown | Unknown |
| *Fusarium globosum* | NRRL 26131 | KF466417.1 | n.a. | n.a. | Unknown | Unknown |
| *Fusarium lactis* | NRRL 25200 | AF160272.1 | NR_111887.1 | U61551.1 | Culture from neotype of *Fusarium lactis* | USA |
| *Fusarium phyllophilum* | NRRL 13617 = PEN6 | KF466421.1 | KR909206.1 | KF466443.1 | Unknown | USA |
| *Fusarium proliferatum* | [NRRL 31071](http://www.biobw.org/China-strain/bio-10474.html) | AF291058.1 | AF291061.1 | AF291055.1 | *[Clivia miniata](https://nt.ars-grin.gov/fungaldatabases/javascript:showExternalData('Clivia miniata')" \o "https://nt.ars-grin.gov/fungaldatabases/javascript:showExternalData('Clivia miniata'))* | China |
| *Fusarium bactridioides* | CBS 100057 | MN533993.1 | KC464615.1 | MT011044.1 | *[Narcissus poeticus](https://nt.ars-grin.gov/fungaldatabases/javascript:showExternalData('Narcissus poeticus')" \o "https://nt.ars-grin.gov/fungaldatabases/javascript:showExternalData('Narcissus poeticus'))* | China |
| *Fusarium annulatum* | CBS 258.54 = PUF023 | HQ165854.1 | HQ165926.1 | MT011041.1 | cornea | China |
| *Fusarium andiyazi* | CBS 119857 | KU604447.1 | KR071651.1 | KU603866.1 | *[Cucumis melo](https://nt.ars-grin.gov/fungaldatabases/javascript:showExternalData('Cucumis melo var. makuwa')" \o "https://nt.ars-grin.gov/fungaldatabases/javascript:showExternalData('Cucumis melo var. makuwa'))* [var. makuwa](https://nt.ars-grin.gov/fungaldatabases/javascript:showExternalData('Cucumis melo var. makuwa')" \o "https://nt.ars-grin.gov/fungaldatabases/javascript:showExternalData('Cucumis melo var. makuwa')) | South Korea |
| *Fusarium oxysporum* | GuangD21 = WZ-176 | MK682409.1 | MN856310.1 | MK682440.1 | bitter gourd | China |
| *Fusarium oxysporum* | GDGZ-1 = DG-2 | KX253983.1 | MK429839.1 | KX253987.1 | Ctenanthe oppenheimiana | China |
| *Fusarium oxysporum* | HuN17 = 14-090 | MF445547.1 | MF445471.1 | LC592361.1 | bitter grand | China |
| *Fusarium solani* | GuangX15 = F174 | KY785023.1 | MF401578.1 | KY785034.1 | Bitter grand | China |
| *Fusarium solani* | FJBX18-3 = D911 | MN295049.1 | MN298761.1 | MH371458.1 | *Passiflora edulis* | China |
| *Fusarium solani* | gss58 = C09 | MH341208.1 | MH290450.1 | MF662654.1 | ginseng | China |
| *Peyronellaea eucalypti* | [CBS 142522](https://www.ncbi.nlm.nih.gov/nuccore/MH553546.1) | KY979893.1 | KY979755.1 | KY979921.1 | *Eucalyptus pellita* | Malaysia |
| *Fusarium solani* | FS-01403 | KJ572787.1 | KJ572781.1 | KJ572782.1 | ginseng | China |
| *Fusarium solani* | MR319 | GQ121907.1 | GQ121887.1 | GQ121902.1 | plant with brown root rot | Argentina |

^1^ATCC: American Type Culture Collection, USA; BBA: Biologische Bundesanstalt für Landund Forstwirtschaft, Berlin-Dahlem, Germany; CBS: Westerdijk Fungal Biodiverity Institute (WIFB), Utrecht, The Netherlands; CPC: Collection of P .W. Crous; DSM: Deutsche Sammlung von Mikroorganismen und Zellkulturen GmbH, Braunschweig, Germany; IHEM: Institute of Hygiene and Epidemiology-Mycology Laboratory, Brussels, Belgium; NRRL: Agricultural Research Service Culture Collection, USA. AC: Personal collection of JN; MAFF: Genetic Resources Center, National Agriculture and Food Research Organization, Tsukuba, Japan; MUCC (Japan): Culture Collection, Laboratory of Plant Pathology, Mie University, Tsu, Japan.

Supplementary Table 6. Related reference strains of *Phoma* species used for phylogenetic analysis.

| Species | Culture accession^1^ | GenBank accession | | Host/substrate | Origin |
| --- | --- | --- | --- | --- | --- |
|  |  | ITS | TUB2 |  |  |
| *Phoma multirostrata* | D/044 | EU573022.1 | EU541430.1 | [Cicer arietinum](https://nt.ars-grin.gov/fungaldatabases/javascript:showExternalData('Cicer arietinum')" \o "https://nt.ars-grin.gov/fungaldatabases/javascript:showExternalData('Cicer arietinum')) | India |
| *Phoma foveata* | D/048 | EU573021.1 | EU541431.1 | *[Solanum tuberosum](https://nt.ars-grin.gov/fungaldatabases/javascript:showExternalData('Solanum tuberosum')" \o "https://nt.ars-grin.gov/fungaldatabases/javascript:showExternalData('Solanum tuberosum'))* | Australia |
| *Phoma plurivora* | ICMP 6875 | EU573019.1 | EU552931.1 | [Dactylis glomerata](https://nt.ars-grin.gov/fungaldatabases/javascript:showExternalData('Dactylis glomerata')" \o "https://nt.ars-grin.gov/fungaldatabases/javascript:showExternalData('Dactylis glomerata')) | New Zealand |
| *Verrucoconiothyrium eucalyptigen* | CBS 142535 | KY979771.1 | KY979935.1 | Eucalyptus | Australia |
| *Phoma fungicola* | 7F90 | KC357253.1 | KC357256.1 | *Ipomoea batatas* | China |
| *Phoma medicaginis* | MP38 | KF181255.1 | KJ396335.1 | *[Lens culinaris](https://nt.ars-grin.gov/fungaldatabases/javascript:showExternalData('Lens culinaris')" \o "https://nt.ars-grin.gov/fungaldatabases/javascript:showExternalData('Lens culinaris'))* | Washington |
| *Phoma macrostoma* | IMI 336757 | DQ474111.1 | n.a. | poplar | China |
| *Phoma glomerata* | PG11 | GU724511.1 | n.a. | poplar | China |
| *Phoma senecionis* | ICMP 10939 | KT309401.1 | KT309812.1 | Unknown | China |
| *Phoma herbarum* | CBS 615.75 | KF251212.1 | KF252703.1 | *Rosa multiflora* | Unknown |
| *Phoma schachtii* | CBS 502.84 | MH861770.1 | n.a. | Unknown | Unknown |
| *Phoma neerlandica* | CBS 134.96 | KT389535.1 | KT389834.2 | *Delphinium* sp. | Unknown |
| *Phoma matteuciicola* | CBS 259.92 | GU237812.1 | GU237627.1 | Unknown | Unknown |
| *Phoma odoratissimi* | CGMCC 3.17488 | KP330435.1 | KP330391.1 | Viburnum odoratissimum | China |
| *Phoma betae* | CBS 109410 | MK249659.1 | MK255063.1 | *Beta vulgaris* | USA |
| *Phoma aloes* | CPC 21549 | KF777183.1 | n.a. | Aloidendron dichotomum | South Africa |
| *Phoma radicina* | VB1-2 | MK764998.1 | n.a. | Alfafa | China |
| *Phoma laundoniae* | IRAN Pr7 | MG701149.1 | MG701150.1 | Unknown | Iran |
| *Phoma digitalis* | CBS 109179 | GU237744.1 | GU237604.1 | Unknown | Unknown |
| *Phoma laundoniae* | ICMP 10843 | KT309802.1 | KT309392.1 | *Prunus persica* | New Zealand |
| *Phoma citri* | P24 | KJ686384.1 | n.a. | Unknown | India |
| *Phoma adonidicola* | LY82 | JQ934841.1 | JQ934853.1 | Adonis palaestina Boiss. | China |
| *Phoma caloplacae* | CBS 129338 | JQ238641.1 | n.a. | Unknown | Unknown |
| *Phoma casuarinae* | I 701 | JN003224.1 | n.a. | Unknown | India |
| *Phoma capsularum* | I 2086 | JN003223.1 | n.a. | Unknown | India |
| *Phoma albiziae* | I 6169 | JN003220.1 | n.a. | Unknown | India |
| *Phoma schneiderae* | VPRI 32175 | DQ660980.1 | n.a. | *Lupinus albus* | Australia |
| *Phoma conidiogena* | CBS 128899 | MH865146.1 | n.a. | Unknown | USA: Wisconsin |
| *Phoma acuta* | CBS 125979 | MH863858.1 | n.a. | Unknown | Unknown |
| *Phoma sojicola* | CBS 100580 | MH862710.1 | n.a. | Unknown | Hungary |
| *Phoma leveillei* | CBS 373.69 | MH871068.1 | n.a. | Unknown | Unknown |
| *Phoma mali* | CBS 350.34 | MH855562.1 | n.a. | Unknown | United Kingdom: Northern Ireland |
| *Phoma aliena* | KACC 49587 | MW412741.1 | n.a. | A leaf of *Ambrosia trifida* | South Korea |
| *Phoma moricola* | MY56 | MT626622.1 | n.a. | Unknown | China |
| *Phoma haematocycla* | BA06 | MH245096.1 | KT309405.1 | *Atricaria chamomilla* | Iran |
| *Phoma insulana* | CBS 252.92 | GU237810.1 | GU237618.1 | Unknown | Unknown |
| *Phoma eupatorii* | CBS 123.93 | GU237764.1 | GU237608.1 | Unknown | Unknown |
| *Phoma acetosellae* | CBS 179.97 | GU237793.1 | GU237575.1 | Unknown | Unknown |
| *Phoma segeticola* | CGMCC 3.17489 | KP330443.1 | KP330399.1 | Cephalonoplos segetum | China |
| *Phoma pinodella* | D/095 | EU573027.1 | EU541418.1 | Unknown | Hungary |
| *Phoma glomerata* | ICMP 15788 | EU573017.1 | EU541426.1 | Unknown | Hungary |
| *Phoma exigua* | ICMP 15330 | EU573008.1 | EU541428.1 | Unknown | Hungary |
| *Phoma pereupyrena* | ICMP 7036 | KT310030.1 | KT309606.1 | Unknown | Niue |
| *Phoma proteae* | ICMP 7056 | KT310039.1 | KT309615.1 | Pennisetum clandestinum | New Zealand |
| *Phoma destructiva* | ICMP 14884 | KT309887.1 | KT309473.1 | *Lycopersicon esculentum* | New Zealand |

^1^See Table S5.

Supplementary Table 7. Related reference strains of *Alternaria* species used for phylogenetic analysis.

| Alternaria section | Fungal name and isolate numbers | Culture accession^1,2^ | Host/substrate | ITS | Origin |
| --- | --- | --- | --- | --- | --- |
| Alternantherae | *Alternaria alternantherae* ( = *Nimbya alternantherae*) | EGS52.039 | *Alternanthera philoxeroides* | JN383496 | Unknown |
|  |  | CBS 124392; HSAUP2798 | *Solanum melongena* | KC584179 | China |
|  | *A. celosiicola* ( = *A. cristata*) | EGS42.013T | Celosia cristata | JN383497 | USA |
|  | *A. gomphrenae* ( = *N. gomphrenae*) | MAFF 246769; MUCC 1623ET | Gomphrena globosa | LC440579 | Japan |
|  | *A. perpunctulata* ( = *N. perpunctulata*) | CBS 115267; EGS51.130T | *Alternanthera philoxeroides* | KC584210 | USA |
| *Alternaria* | *A. alstroemeriae* | MAFF 241374 | *Alstroemeria sp.* | AB678214 | Japan |
|  |  | CBS 118809; EGS52.068T | *Alstroemeria sp.* | KP124297 | Austria |
|  | *A. alternata* | CBS 916.96; EGS34.016ET | *Arachis hypogaea* | AF347031 | India |
|  | *A. alternata f. sp. citri pathotype rough lemon* ( = *A. limoniasperaeT*) | CBS 102595; EGS45.100; BMP0316 | *Citrus jambhiri* | FJ266476 | USA |
|  | *A. alternata f. sp. citri pathotype tangerine* ( = *A. toxicogenicaT*) | CBS 102600; EGS39.181; ATCC 38963 | *Citrus reticulata* | KP124331 | USA |
|  | *A. alternata f. sp. mali* ( = *A. maliT*) | CBS 106.24; EGS38.029; ATCC 13963 | *Malus sylvestris* | KP124298 | USA |
|  | *A. alternata* | MHE16MC | Pelargonium leaves and roots | KY617045.1 | South Africa |
|  | *A. arborescens species complex* | CBS 102605; EGS39.128; BMP0308T ( = *A.alternata* tomato pathotype) | *Solanum lycopersicum* | AF347033 | USA |
|  | *A. betae-kenyensis* | CBS 118810; EGS49.159T | *Beta vulgaris* var. cicla | KP124419 | Kenya |
|  | *A. burnsii* | CBS 107.38; EGS06.185T | Cuminum cyminum | KP124420 | India |
|  | *A. cylindrica^*^* | MAFF 246770T | *Petunia × atkinsiana* | LC440584 | Japan |
|  | *A. eichhorniae* | CBS 489.92; ATCC 22255T | *Eichhornia crassipes* | KC146356 | India |
|  | *A. gaisen* f. sp. *fragariae* ( = *A. alternata strawberry pathotype*) | MAFF 242310; MUCC 1609 | *Fragaria × ananassa* ‘HS-138’ | LC269973 | Japan |
|  | *A. gaisen* f. sp. *pyri* ( = *A. alternata Japanese pear pathotype*) | CBS 118488; EGS90.0391ET | *Pyrus pyrifolia* var. culta ‘Nijisseiki’ | KP124427 | Japan |
|  | *A. gossypina* | CBS 104.32T | *Gossypium sp.* | KP124430 | Zimbabwe |
|  | *A. iridiaustralis* | CBS 118486T; EGS43.014 | *Iris sp.* | KP124435 | Austria |
|  | *A. iridicola* | MAFF 246890; MUCC 2149ET | Japan, Iris japonica | LC269975 | Japan |
|  | *A. jacinthicola* | CBS 133751T | *Eichhornia crassipes* | KP124438 | Mali |
|  | *A. longipes* ( = *A. alternata tobacco pathotype*) | CBS 540.94; EGS30.033R | USA, *Nicotiana tabacum* | AY278835 | USA |
|  |  | CBS 121332; EGS30.048R | USA, *Nicotiana tabacum* | KP124443 | USA |
|  | *A. tomato* | CBS 114.35 | Unknown, *Solanum lycopersicum* | KP124446 | Unknown |
| Brassicicola | *A. brassicicola* | MAFF 246772; MUCC 1694 | Japan, *Brassica oleracea* var. sabellica | LC440585 | Japan |
|  | *A. conoidea* ( = *Embellisia conoidea*) | CBS 132.89 | *Ricinus communis* | FJ348226 | Saudi Arabia |
|  | *A. mimicula* | CBS 118696; EGS01.056; BMP0324T | USA, *Solanum lycopersicum* | FJ266477 | USA |
|  | *A. septorioides* | CBS 106.41; EGS52.089T | *Reseda odorata* | KC584216 | Netherlands |
|  | *A. solidaccana* | CBS 118698; EGS36.158T | Soil | KC584219 | Bangladesh |
| Chalastospora | *A. cetera* ( = *Chalastospora cetera*) | CBS 121340; CBS 110898; EGS41.072; BMP0033T | *Elymus scabrus* | JN383482 | Austria |
| Cheiranthus | *A. cheiranthi* | CBS 109384; EGS41.188; BMP0148; BMP0148R Italy, Cheiranthus cheiri | Unknown | KC584107 | Unknown |
| Crivellia | *A. papavericola* ( = *Crivellia homothallica, Brachycladium papaveris*) | CBS 116606; P351T | *Papaver somniferum* | FJ357310 | USA |
|  | *A. penicillata* ( = *Cr. papaveracea, B.penicillatum*) | CBS 116608; P354.8ET | *Papaver rhoeas* | FJ357311 | Austria |
| Dianthicola | *A. dianthicola* | CBS 116491; EGS51.022R | *Dianthus × allwoodii* | KC584194 | New Zealand |
| Embellisia | *A. embellisia* ( = *E. allii*) | CBS 339.71R | *Allium sativum* | KC584230 | USA |
| Embellisioides | *A. hyacinthi* ( = *E. hyacinthi*) | CBS 416.71; EGS19.102T | *Hyacinthus orientalis* | KC584233 | Netherlands |
| Euphorbiicola | *A. euphorbiicola* | CBS 119410; EGS41.029R | *Euphorbia pulcherrima* | KJ718173 | USA |
|  |  | CBS 121329; EGS04.1581T | *Cuminum cyminum* | KC584191 | India |
|  | *A. eureka* ( = *E. eureka*) | CBS 193.86; EGS36.103T | *Medicago rugosa* | JN383490 | Australia |
| Gypsophilae | *A. ellipsoidea* | CBS 119674; EGS49.104T | *Dianthus barbatus* | KC584196 | USA |
|  | *A. gypsophilae* | CBS 107.41; EGS07.025T | Unknown, *Gypsophila elegans* | KC584199 | Unknown |
|  | *A. nobilis* | AC1 | Japan, Dianthus barbatus | LC440592 | Japan |
|  | *A. vaccariicola* | CBS 118714; EGS46.003T | USA, Vaccaria hispanica | KC584224 | USA |
| Infectoriae | *A. infectoria* | CBS 210.86; EGS27.193T | *Triticum aestivum* | AF347034 | UK |
| Japonicae | *A. japonica* | MAFF 246775; MUCC 1622ET | Japan, Raphanus sativus | LC440595 | Japan |
|  | *A. nepalensis* | CBS 118700; EGS45.073T | *Brassica sp.* | KC584207 | Nepal |
| Nimbya | *A. scirpicola* ( = *N. scirpicola*) | CBS 481.90; EGS19.042R | *Scirpus sp.* | KC584237 | UK |
| Panax | *A. avenicola* | CBS 121459; EGS50.185T | *Avena sp.* | KC584183 | Norway |
|  | *A. dendropanacis* | CNU 085031T | *Dendropanax morbifer* | HQ203210 | Korea |
|  | *A. eryngii* | CBS 121339; EGS41.005; BMP0336R | *Eryngium sp.* | JQ693661 | Unknown |
|  | *A. panax* | CBS 482.81; EGS29.180R | *Aralia racemosa* | KC584209 | USA |
|  | *A. photistica* | CBS 212.86; EGS35.172; BMP0041T | *Digitalis purpurea* | KC584212 | UK |
| Phragmosporae | *A. phragmospora* ( = *E. phragmospora*) | CBS 274.70; EGS27.098T | Soil | JN383493 | Netherlands |
| Porri | *Alternaria allii* | CBS 107.28; EGS48.084T | *Allium cepa* | KJ718100 | Puerto Rico |
|  |  | CBS 116701; EGS33.134R | *Allium cepa* var. viviparum | KJ718103 | USA |
|  | *A. crassa* | MAFF 243056 | Japan, Datura stramonium | AB678215 | Japan |
|  |  | CBS 110.38ET | Datura stramonium | KJ718147 | Cyprus |
|  |  | CBS 109160; EGS45.075; BMP0180 ( = *A.capsici*) | *Capsicum annuum* | KJ718148 | Australia |
|  | *A. cucumerina* | CBS 117225; EGS41.127R | *Cucumis melo* | KJ718154 | USA |
|  |  | CBS 116114; EGS35.123 ( = *A. loofahae*T) | *Luffa acutangula* | KJ718153 | USA |
|  | *A. dauci* | CBS 111.38NT | *Daucus carota* | KJ718158 | Italy |
|  | *A. macrospora* | CBS 117228; EGS50.190T | Gossypium barbadense | KC584204 | USA |
|  | *A. porri* | CBS 116699; EGS48.152ET | *Allium cepa* | KJ718218 | USA |
|  |  | CBS 116698; EGS48.147R | *Allium cepa* | DQ323700 | USA |
|  | *A. pseudorostrata* | CBS 119411; EGS42.060; BMP0174T | *Euphorbia pulcherrima* | JN383483 | USA |
|  | *A. solani* | CBS 109157; EGS44.098R | *Solanum tuberosum* | KJ718238 | USA |
|  | *A. tagetica* | CBS 479.81; EGS33.081R | *Tagetes erecta* | KC584221 | UK |
|  | *A. zinniae* | CBS 117223; EGS44.035R | *Zinnia elegans* | KJ718270 | New Zealand |
| Pseudoalternaria | *A. rosae* | CBS 121341; EGS41.130T | *Rosa rubiginosa* | JQ693639 | New Zealand |
| Pseudoulocladium | *A. chartarum* ( = *Ulocladium chartarum*) | CBS 200.67; ATCC 18044; BMP0359ET | *Populus sp.* | AF229488 | Canada |
|  | *A. aspera* ( = *Ul. arborescens*) | CBS 115269; EGS44.109T | *Pistacia vera* | KC584242 | Japan |
|  | *A. concatenata* ( = *Ul. capsici*) | CBS 120006; HSAUPIII00035T | *Capsicum annuum* | KC584246 | China |
|  | *A. septospora* ( = *Ul. septosporum* ) | CBS 109.38 | wood pulp | FJ266489 | Italy |
| Radicina | *A. petroselini* | CBS 112.41; EGS06.196T | *Petroselinum sativum* | KC584211 | Unknown |
|  |  | CBS 109383; EGS09.159; BMP0144R | *Petroselinum crispum* | AF229454 | USA |
|  | *A. radicina* | CBS 245.67; EGS03.145; ATCC 6503NT | *Daucus carota* | KC584213 | USA |
|  | *A. selini* | CBS 109382; EGS25.198T | *Petroselinum crispum* | AF229455 | Saudi Arabia |
|  | *A. smyrnii* | CBS 109380; EGS37.093; BMP0147R | *Smyrnium olusatrum* | AF229456 | UK |
| Soda | *A. kulundii* | CBS 137525; M313T | Soil | KJ443262 | Russia |
| Sonchi | *A. cinerariae* | MAFF 243059; MUCC 1701ET | Pericallis cruenta | AB906673 | Japan |
|  |  | MAFF 241266; MUCC 1613 | Farfugium japonicum | LC440619 | Japan |
|  | *A. sonchi* | CBS 119675; EGS43.131R | Sonchus asper | KC584220 | Canada |
| Teretispora | *A. leucanthemi* ( = *Teretispora leucanthemi*) | CBS 421.65; ATCC 16028; EGS10.059T | Chrysanthemum maximum | KC584240 | Netherlands |
| Ulocladioides | *A. atra* ( = *Ul. atrum*) | CBS 195.67; ATCC 18040; BMP0355ET | Soil | AF229486 | USA |
|  | *A. cucurbitae* ( = *Ul. cucurbitae*) | CBS 483.81; EGS31.021; BMP0351R | *Cucumis sativus* | FJ266483 | New Zealand |
|  | *A. multiformis* ( = *Ul. multiforme*) | CBS 102060; EGS31.005T | Soil | FJ266486 | Canada |
|  | *A. cantlous* ( = *Ul. cantlous*) | CBS 123007; HSAUP0209T | Cucumis melo | KC584245 | China |
|  | *A. heterospora* ( = *Ul. solani*) | CBS 123376; HSAUP 0521T | *Solanum lycopersicum* | KC584248 | China |
| Ulocladium | *A. alternariae* ( = *Sinomyces alternariae*) | CBS 126989; EGS46.004 | *Daucus carota* | AY376642 | USA |
|  | *A. botrytis* ( = *Ul. botrytis*) | MAFF 246887 | *Asparagus officinalis* | LC440625 | Japan |
|  |  | CBS 197.67; ATCC 18042ET | Air | KC584243 | USA |
|  | *A. oudemansii* ( = *Ul. oudemansii*) | CBS 114.07; ATCC 18047; IMI 124940; MUCL 18563; QM 1744T | Unknown | FJ266488 | Unknown |
| Undifilum | *A. bornmuelleri* ( = *Undifilum bornmuelleri*) | DAOM 231361 | Securigera varia | FJ357317 | Austria |
| Monotypic lineage | *A. argyranthemi* | CBS 116530; EGS44.033T | *Argyranthemum sp.* | KC584181 | New Zealand |
|  | *A. brassicae* | AC29 | *Brassica rapa* | LC440626 | Japan |
|  |  | MAFF 240791 | *Raphanus sativus* | LC440627 | Japan |
|  | *A. dennisii* ( = *E. dennisii*) | CBS 476.90; EGS30.121T | Isle of Man, Senecio jacobaea | JN383488 | Unknown |
|  | *A. helianthiinficiens* | CBS 208.86; EGS36.184T | *Helianthus annuus* | JX101649 | USA |
|  | *A. peucedani* | CNU 111485T | Peucedanum japonicum | KF728231 | Korea |
|  | *A. soliaridae* | CBS 118387; EGS33.024T | Soil | KC584218 | USA |
|  | *A. thalictrigena* | CBS 121712; CPC 13410T | *Thalictrum sp.* | EU040211 | Germany |
|  | *A. thlaspis* ( = *E. thlaspis*) | EGS45.069T | Thlaspis caerulescentis | JN383495 | UK |
|  | *A. triangularis^*^* | MAFF 246776T | Japan, Bupleurum rotundifolium | LC440629 | Japan |
| Out group | *Paradendryphiella salina* ( = *E. annulata*) | CBS 302.84T | Cancer pagurus | JN383486 | North Sea |

^1^AC: Personal collection of JN; ATCC: American Type Culture Collection, Virginia, USA; BMP: Personal collection of Dr . B.M. Pryor, School of Plant Sciences, University of Arizona, Arizona, USA; CBS: Westerdijk Fungal Biodiversity Institute, Utrecht, The Netherlands; CNU: Culture Collection of Chungnam National University, Daejeon, Korea; CPC: Personal collection of Dr . P.W. Crous, housed at CBS; DAOM: Canadian Collection of Fungal Cultures, Ottawa, Canada; EGS: Personal collection of Dr . E.G. Simmons; HSAUP: Department of Plant Pathology, Shandong Agricultural University, China; MAFF: Genetic Resources Center, National Agriculture and Food Research Organization, Tsukuba, Japan; MUCC (Japan): Culture Collection, Laboratory of Plant Pathology, Mie University, Tsu, Japan; P: Personal collection of Dr. P. Inderbitzin, Department of Plant Pathology, Cornell University, New York, USA.

^2^Ex-type, - neotype, and -epitype strain indicated with T, NT, and ET; R: representative strain by Simmons (2007). Fungal names between parentheses refer to the former name or the name under the pathotype concept (Nishimura 1980).

^*^Novel taxa proposed in the taxonomy section.

Supplementary Table 8. Related reference strains of *Aspergillus* species used for phylogenetic analysis.

| Species | Culture accession^1^ | GenBank accession | | Host/substrate | Origin |
| --- | --- | --- | --- | --- | --- |
|  |  | ITS | TUB2 |  |  |
| *Aspergillus oryzae* | DG-B4 | HQ285549 | HQ285488 | Unknown | Unknown |
| *Aspergillus flavus* | USMG16 | KF434091 | KF434080 | Unknown | Unknown |
| *Aspergillus hiratsukae* | HMCC-3 | MH644011 | MH644026 | *Lolium multiflorum* | China |
| *Aspergillus ochraceus* | HMCC-14 | MH644012 | MH644027 | *Lolium multiflorum* | China |
| *Aspergillus amoenus* | NRRL 4838 | EF652480 | JN853946 | Unknown | Unknown |
| *A. arenarioides* | CBS 138195 = DTO 129G8 | KJ775557 | KJ775070 | indoor house dust | Thailand |
|  | CBS 138196 = DTO 267B6 | KJ775558 | KJ775082 | indoor house dust | Micronesia |
|  | CBS 138197 = DTO 267C7 | KJ775559 | KJ775083 | indoor house dust | Micronesia |
|  | CBS 138198 = DTO 268E1 | KJ775560 | KJ775089 | indoor house dust | Micronesia |
|  | CBS 138199 = DTO 268E2 | KJ775561 | KJ775090 | indoor house dust | Micronesia |
|  | CBS 138200 = DTO 268E3 | KJ775562 | KJ775091 | indoor house dust | Micronesia |
| *A. arenarius* | CBS 463.65 = NRRL 5012 = ATCC 16830 = IMI 055632 = IMI 055632ii = WB | EU021615 | EU021674 | Unknown | Unknown |
| *A. aureofulgens* | CBS 653.74 = NRRL 6326 | EF669617 | EU014079 | Unknown | Unknown |
| *A. austroafricanus* | NRRL 233 | JQ301891 | JN853963 | Unknown | Unknown |
| *A. baeticus* | NRRL 62501 = CCF 4226 = CMFISB 2153 | HE615086 | HE615092 | Unknown | Unknown |
| *A. brevijanus* | CBS 111.46 = NRRL 1935 = ATCC 16828 = CBS 119.45 = IMI 016066ii = IMI | EF669582 | EU014078 | Unknown | Unknown |
| *A. brunneus* | CBS 112.26 = CBS 524.65 = NRRL 131 = NRRL 134 = ATCC 1021 = IFO5862 = IMI 211378 = QM 7406 = Thom 4481 = Thom 5633.4 = WB 131 | EF652060 | EF651907 | Unknown | Unknown |
|  | NRRL 133 | EF652061 | EF651908 | Unknown | Unknown |
| *A. campestris* | CBS 348.81 = NRRL 13001 = ATCC 44563 = IMI 259099 | EF669577 | EU014091 | Unknown | Unknown |
| *A. candidus* | CBS 566.65 = NRRL 303 = ATCC 1002 = IMI 16264 = IMI 91889 = LSHBAc .27 = NCTC 595 = QM 1995 = Thom 106 = WB 303NRRL 4646 | EF669592 | EU014089 | Unknown | Unknown |
|  | NRRL 4646 | EF669605 | EU014090 | Unknown | Unknown |
| *A. capensis* | CBS 138188 = DTO 179E6 | KJ775550 | KJ775072 | indoor house dust | Unknown |
| *A. compatibilis* | CBS 488.65 = NRRL 5096 = ATCC 16847 = IMI 139277 = QM 8916 = WB 5096 | EF652499 | EF652323 | Unknown | Unknown |
| *A. creber* | NRRL 58592 | JQ301889 | JN853980 | Unknown | Unknown |
| *A. cvjetkovicii* | NRRL 227 | EF652440 | EF652264 | Unknown | Unknown |
| *A. flavipes* | NRRL 302 = ATCC 24487 = IMI 171885 = QM 9566 = Thom 4640.474 = WB 302 | EF669591 | EU014085 | Unknown | Unknown |
| *A. fructus* | NRRL 239 | EF652449 | EF652273 | Unknown | Unknown |
| *A. fruticans* | CBS 486.65 = NRRL 4903 = ATCC 16823 = IMI 139279 = O-1077 = QM | EF652483 | EF652307 | Unknown | Unknown |
| *A. glaucus* | CBS 516.65 = NRRL 116 = ATCC 16469 = IMI 211383 = LCP 64 859 = Thom | EF652052 | EF651887 | Unknown | Unknown |
|  | NRRL 120 | EF652054 | EF651889 | Unknown | Unknown |
|  | NRRL 121 | EF652055 | EF651890 | Unknown | Unknown |
| *A. griseoaurantiacus* | CBS 138189 = DTO 245F5 | KJ775551 | KJ775079 | indoor house dust | Mexico |
|  | CBS 138190 = DTO 267D2 | KJ775552 | KJ775084 | Unknown | Unknown |
|  | CBS 138191 = DTO 267D8 | KJ775553 | KJ775086 | indoor house dust | Micronesia |
| *A. iizukae* | CBS 541.69 = NRRL 3750 = IMI 141552 = QM 9325 | EF669597 | EU014086 | Unknown | Unknown |
|  | NRRL 35046 | EF669596 | EU014087 | Unknown | Unknown |
| *A. janus* | CBS 118.45 = NRRL 1787 = IMI 16065 = NCTC 6970 | EF669578 | EU014076 | Unknown | Unknown |
| *A. jensenii* | NRRL 58600 | JQ301892 | JN854007 | Unknown | Unknown |
| *A. micronesiensis* | CBS 138182 = DTO 245D7 | KJ775546 | KJ775078 | indoor house dust | Mexico |
|  | CBS 138183 = DTO 267D5 | KJ775548 | KJ775085 | indoor house dust | Micronesia |
|  | CBS 138186 = DTO 267H5 | KJ775549 | KJ775088 | indoor house dust | Thailand |
|  | NRRL 295 | EF669588 | EU014081 | Unknown | Unknown |
|  | NRRL 4263 | EF669600 | EU014083 | Unknown | Unknown |
|  | NRRL 4578 | EF669602 | EU014082 | Unknown | Unknown |
| *A. niveoglaucus* | CBS 101750 | HE615135 | HE801331 | Unknown | Unknown |
|  | CBS 114.27 = CBS 517.65 = NRRL 127 = ATCC 10075 = IMI 32050 = LSHBA | EF652058 | EF651905 | Unknown | Unknown |
|  | NRRL 128 | EF652059 | EF651906 | Unknown | Unknown |
|  | NRRL 136 | EF652062 | EF651909 | Unknown | Unknown |
|  | NRRL 137 | EF652063 | EF651910 | Unknown | Unknown |
| *A. porphyreostipitatus* | CBS 138202 = DTO 132D1 | KJ775563 | KJ775071 | indoor house dust | Thailand |
|  | CBS 138203 = DTO 266D9 | KJ775564 | KJ775080 | indoor house dust | Mexico |
| *A. proliferans* | CBS 121.45 = NRRL 1908 = IMI 016105ii = IMI 016105iii = IMI 16105 = LSHB | EF652064 | EF651891 | Unknown | Unknown |
|  | NRRL 114 | EF652051 | EF651886 | Unknown | Unknown |
|  | NRRL 117 | EF652053 | EF651888 | Unknown | Unknown |
| *A. protuberus* | CBS 602.74 = NRRL 3505 = ATCC 18990 = QM 9804 | EF652460 | EF652284 | Unknown | Unknown |
| *A. pseudoglaucus* | CBS 123.28 = NRRL 40 = ATCC 10066 = IMI 016122 = IMI 016122ii = LSHBA | EF652050 | EF651917 | Unknown | Unknown |
| *A. pseudoustus* | CBS 123904 = NRRL 5856 = IBT 28161 | FJ531147 | FJ531168 | Unknown | Unknown |
| *A. puniceus* | CBS 495.65 = NRRL 5077 = ATCC 16800 = IMI 126692 = QM 9812 = WB 5077 | EF652498 | EF652322 | Unknown | Unknown |
|  | NRRL 1852 | EF652425 | EF652249 | Unknown | Unknown |
|  | NRRL 4688 | EF652469 | EF652293 | Unknown | Unknown |
| *A. puulaauensis* | NRRL 35641 | JQ301893 | JN853979 | Unknown | Unknown |
| *A. ruber* | CBS 530.65 = NRRL 52 = ATCC 16441 = IMI 211380 = QM 1973 = Thom | EF652066 | EF651920 | Unknown | Unknown |
| *A. saccharolyticus* | CBS 127449 = IBT 28509 | HM853552 | HM853553 | Unknown | Unknown |
| *A. sloanii* | CBS 138176 = DTO 244I8 | KJ775539 | KJ775073 | indoor house dust | United Kingdom |
|  | CBS 138177 = DTO 245A1 | KJ775540 | KJ775074 | indoor house dust | United Kingdom |
|  | CBS 138231 = DTO 245A6 | KJ775541 | KJ775075 | indoor house dust | United Kingdom |
|  | CBS 138178 = DTO 245A8 | KJ775542 | KJ775076 | indoor house dust | United Kingdom |
|  | CBS 138179 = DTO 245A9 | KJ775543 | KJ775077 | indoor house dust | United Kingdom |
| *A. subalbidus* | CBS 567.65 | KJ866983 | EU076295 | Unknown | Unknown |
|  | CBS 138192 = DTO 129E3 | KJ775554 | KJ775068 | indoor house dust | Thailand |
|  | CBS 138193 = DTO 129F9 | KJ775555 | KJ775069 | indoor house dust | Thailand |
|  | CBS 138194 = DTO 266I9 | KJ775556 | KJ775081 | indoor house dust | Micronesia |
|  | NRRL 4809 | EF669609 | EU014092 | Unknown | Unknown |
| *A. subversicolor* | NRRL 58999 | JQ301894 | JN853970 | Unknown | Unknown |
| *A. sydowii* | CBS 593.65 = NRRL 250 = IMI 211384 = NRRL 254 | EF652450 | EF652274 | Unknown | Unknown |
| *A. tabacinus* | CBS 122718 = NRRL 4791 = IFO 4098 = QM 9766 = WB 4791 | EF652478 | EF652302 | Unknown | Unknown |
| *A. taichungensis* | DTO 266G2 | KJ775572 | KJ866980 | Unknown | Unknown |
|  | DTO 270C9 | KJ775573 | KJ866981 | Unknown | Unknown |
|  | IBT 19404 | EU076301 | EU076297 | Unknown | Unknown |
| *A. tanneri* | NRRL 62426 = NIH 1005 | JN853798 | JN896582 | Unknown | Unknown |
| *A. templicola* | CBS 138180 = DTO 267H4 | KJ775544 | KJ775087 | indoor house dust | Thailand |
|  | CBS 138180 = DTO 270C6 | KJ775545 | KJ775092 | indoor house dust | Mexico |
| *A. tennesseensis* | NRRL 13150 | JQ301895 | JN853976 | Unknown | Unknown |
| *A. tonophilus* | CBS 405.65 = NRRL 5124 = ATCC 16440 = ATCC 36504 = IMI 108299 = QM | EF652081 | EF651919 | Unknown | Unknown |
| *A. tritici* | CBS 266.81 | EU076302 | EU076293 | Unknown | Unknown |
|  | NRRL 313 | EF669594 | EU014093 | Unknown | Unknown |
| *A. ustus* | NRRL 4991 | EF652492 | EF652316 | Unknown | Unknown |
|  | CBS 261.67 = NRRL 275 = ATCC 1041 = ATCC 16818 = IMI 211805 = QM | EF652455 | EF652279 | Unknown | Unknown |
| *A. venenatus* | NRRL 13147 | JQ301896 | JN854003 | Unknown | Unknown |
| *A. versicolor* | CBS 583.65 = NRRL 238 = ATCC 9577 = IFO 33027 = IMI 229970 = JCM | EF652442 | EF652266 | Unknown | Unknown |
| *A. xerophilus* | CBS 938.73 = NRRL 6131 | EF652085 | EF651923 | Unknown | Unknown |
| *Didymella glomerata* | CBS 284.76 | FJ427005.1 | FJ427116.1 | Populus nigra | Russia |

^1^See Table S5.

Supplementary Table 9. Related reference strains of *Penicillium* species used for phylogenetic analysis.

| Species | Culture accession^1^ | GenBank accession | | Host/substrate | Origin |
| --- | --- | --- | --- | --- | --- |
|  |  | ITS | TUB2 |  |  |
| *Penicillium brasilianum* | [XZ94](http://www.biobw.org/China-strain/bio-11326.html) | MF039289 | MF036174 | Unknown | Unknown |
| *Penicillium onobense* | CMV006B5 | MK450706 | MK451076 | soil | South Africa |
| *Penicillium panissanguineum* | [KAS 2208](https://www.ncbi.nlm.nih.gov/nuccore/MN969182.1) | KT887861 | KT887822 | termite mounds | Tanzania |
| *Penicillium alagoense* | URM 8086 | MK804502 | MK802333 | endophytic fungi | Brazil |
| *Penicillium simplicissimum* | [IBT 15303](http://www.biobw.org/China-strain/bio-11326.html) | AF203084 | DQ834935 | Unknown | Unknown |
| *Penicillium cataractum* | [KAS 2145](https://www.ncbi.nlm.nih.gov/nuccore/MN969180.1) | KT887847 | KT887808 | Unknown | Canada |
| *Penicillium wandoense* | CNUFC - WT31 - 1 | n.a. | MK080564 | Unknown | Unknown |
| *Penicillium infrabuccalum* | KAS 2168 | KT887849 | KT887810 | ants | Canada |
| *Penicillium araracuarense* | [CBS 113149](https://www.ncbi.nlm.nih.gov/nuccore/GU981642.1) | GU981594 | GU981639 | leaf litter exposed in a litter bag | Colombia |
| *Penicillium caperatum* | [CMV012C4](https://www.ncbi.nlm.nih.gov/nuccore/MN969242.1) | MK450678 | MK451245 | soil | South Africa |
| *Penicillium vasconiae* | CBS 339.79 | MH861218 | GU981653 | Unknown | Spain |
| *Penicillium pedernalense* | [F01-11](https://www.ncbi.nlm.nih.gov/nuccore/MH878176.1) | KU255398 | KU255396 | shrimp heads waste composting | Ecuador |
| *Penicillium reticulisporum* | CBS 122.68 | n.a. | MN969394 | Unknown | Japan |
| *Penicillium ochrochloron* | DUCC4131 | n.a. | MH844696 | Unknown | Unknown |
| *Penicillium tanzanicum* | [KAS 1946](https://www.ncbi.nlm.nih.gov/nuccore/NR_158820.1) | KT887841 | KT887802 | Unknown | Tanzania |
| *P. alfredii* | CBS 138224 = DTO 269A4 | KJ775684 | KJ775177 | indoor house dust | Micronesia |
| *P. atramentosum* | CBS 109588 = DTO 249C3 | n.a. | KJ866976 | Unknown | Unknown |
|  | CBS 109601 = DTO 249C4 | n.a. | KJ866977 | Unknown | Unknown |
|  | CBS 109611 = IBT 10565 | n.a. | KJ866972 | Unknown | Unknown |
|  | CBS 109612 = IBT 14762 | n.a. | KJ866973 | Unknown | Unknown |
|  | CBS 109613 = DTO 250G3 | n.a. | KJ866978 | Unknown | Unknown |
|  | CBS 194.88 = IBT 21504 | n.a. | KJ866974 | Unknown | Unknown |
|  | CBS 490.84 = IBT 11800 | n.a. | KJ866975 | Unknown | Unknown |
|  | DTO 178G2 | n.a. | KJ775095 | indoor house dust | South Africa |
| *P. atrovenetum* | CBS 243.56 | n.a. | KJ866971 | Unknown | Unknown |
| *P. brefeldianum* | CBS 235.81 = NRRL 710 = FRR 710 = IFO 31731 = IMI 216896 = LCP | n.a. | GU981623 | Unknown | Unknown |
| *P. canescens* | CBS 300.48 = ATCC 10419 = DSM1215 = FRR 910 = IMI 028260 = MUCL | n.a. | JX140946 | Unknown | Unknown |
|  | NRRL 35656 | n.a. | DQ658166 | Unknown | Unknown |
| *P. chermesinum* | CBS 231.81 = NRRL 2048 = FRR 2048 = IFO 31745 = IMI 191730 | AY742693 | KJ834441 | coffee plant | USA |
| *P. cinnamopurpureum* | CBS 429.65 = CBS 847.68 = NRRL 162 = ATCC 18489 = CSIR 936 = FAT | EF626950 | EF626948 | the conidial heads of *Aspergillus* species | USA |
| *P. coralligerum* | CBS 114.69 | n.a. | KJ866970 | Unknown | Unknown |
|  | CBS 123.65 = ATCC 16968 = FRR 3465 = IFO 9578 = IHEM 4511 = IMI | n.a. | KJ834444 | culture from neotype of *Penicillium coralligerum* | France |
| *P. crystallinum* | CBS 479.65 = NRRL 5082 = ATCC 16833 = IMI 139270 | n.a. | EF669682 | culture from neotype of *Penicillium coralligerum* | USA |
| *P. dunedinense* | CBS 138218 = DTO 244G1 | n.a. | KJ775171 | indoor house dust | New Zealand |
| *P. echinulatum* | NRRL 917 | n.a. | KJ866964 | Unknown | Unknown |
| *P. ellipsoideosporum* | CBS 112493 = AS 3.5688 | JX012224 | JQ965104 | Unknown | Unknown |
| *P. granatense* | CBS 166.81 | n.a. | KJ866967 | Unknown | Unknown |
| *P. guizhouanum* | AS 3.5215 | KJ890410 | KJ890408 | Unknown | Unknown |
| *P. idahoense* | CBS 341.68 = NRRL 5274 = ATCC 22055 = FRR 881 = IMI 148393 | KC411747 | EF626953 | Unknown | Unknown |
| *P. incoloratum* | CBS 101753 = AS 3.4672 | KJ834508 | KJ834457 | Phaseolus angularis seed | China |
|  | DTO 129G5 | KJ775689 | KJ775182 | indoor house dust | Thailand |
|  | DTO 129I1 | KJ775690 | KJ775183 | indoor house dust | Thailand |
| *P. infrapurpureum* | CBS 138219 = DTO 235F6 | KJ775679 | KJ775172 | indoor house dust | Australia |
|  | CBS 138220 = DTO 235G2 | KJ775680 | KJ775173 | indoor house dust | Australia |
|  | CBS 138221 = DTO 235G5 | KJ775681 | KJ775174 | indoor house dust | Australia |
|  | CBS 138222 = DTO 235G6 | KJ775682 | KJ775175 | indoor house dust | Australia |
|  | CBS 138223 = DTO 235H5 | KJ775683 | KJ775176 | indoor house dust | Australia |
| *P. jamesonlandense* | CBS 102888 = DAOM 234087 = IBT 21984 = IBT 24411 | DQ267912 | DQ309448 | culture from neotype of *Penicillium coralligerum* | Unknown |
| *P. janczewskii* | CBS 221.28 = FRR 919 = IMI 191499 = NRRL 919 | n.a. | KJ834460 | soil under Pinus | Poland |
|  | CBS 279.47 | n.a. | KJ866968 | Unknown | Unknown |
|  | CBS 413.68 | n.a. | KJ866969 | Unknown | Unknown |
|  | CBS 414.68 | n.a. | KJ866960 | Unknown | Unknown |
|  | CBS 458.69 | n.a. | KJ86696 | Unknown | Unknown |
| *P. janthinellum* | CBS 340.48 = ATCC 10455 = IMI 040238 = NRRL 2016 = QM 6865 | GU981585 | GU981625 | culture from neotype of *Penicillium coralligerum* | Unknown |
| *P. javanicum* | CBS 341.48 = ATCC 9099 = CSIR 831 = FRR 707 = IFO 31735 = IMI | GU981613 | GU981657 | culture from neotype of *Penicillium coralligerum* | Unknown |
| *P. jensenii* | CBS 216.28 | n.a. | KJ866963 | Unknown | Unknown |
|  | CBS 327.59 = ATCC 18317 = FRR 909 = IFO 5764 = IMI 039768 = LCP | n.a. | JX140954 | Unknown | South Africa |
| *P. jianxiense* | AS 3.6521 | KJ890411 | KJ890409 | Unknown | Unknown |
| *P. kojigenum* | CBS 345.61 = ATCC 18227 = CCRC 31515 = FRR 3442 = IFO 9581 = IMI | AF033489 | KJ834463 | Roadside soil | United Kingdom: Scotland |
| *P. lanosum* | CBS 106.1 = ATCC 10458 = FRR 2009 = IFO 5851 = IFO 6099 = IMI | DQ304540 | DQ285627 | strain received from D. Malloch, University of Toronto | Canada |
| *P. lenticrescens* | CBS 138215 = DTO 129A8 | KJ775675 | KJ775168 | indoor house dust | New Zealand |
| *P. magnielliptisporum* | CBS 138225 = DTO 128H8 | n.a. | KJ775179 | indoor house dust | New Zealand |
| *P. malacaense* | CBS 160.81 = NRRL 35754 = ATCC 42241 = IJFM 7093 = IMI 253801 = VKMF- | n.a. | EU427268 | culture from neotype of *Penicillium coralligerum* | USA |
| *P. malodoratum* | CBS 490.65 = NRRL 5083 = IMI 172289 = ATCC 16834 | n.a. | EF669681 | culture from neotype of *Penicillium malodoratum* | USA |
| *P. mexicanum* | CBS 138227 = DTO 270F1 | n.a. | KJ775178 | culture from neotype of *Penicillium mexicanum* | Netherlands |
| *P. nigricans* | CBS 354.48 | n.a. | KJ866965 | Unknown | Unknown |
| *P. nigricans var. sulphureum* | CBS 744.70 | n.a. | KJ866966 | Unknown | Unknown |
| *P. nodulum* | CBS 227.89 | KC411703 | KJ834475 | type material of *Penicillium nodulum* | Netherlands |
| *P. novae-zeelandiae* | CBS 137.41 = ATCC 10473 = IFO 31748 = IMI 040584ii = NRRL 2128 = QM | n.a. | KJ834477 | Apothecium of *Sclerotinia* | New Zealand |
| *P. oxalicum* | CBS 219.30 = 1934 = VKMF-2886 | AF033438 | KF296462 | the fynbos biome | South Africa |
| *P. paradoxum* | NRRL 2162 = QM 7606 | n.a. | EF669683 | four loci | USA |
| *P. parvulum* | CBS 132825 = NRRL 35504 | EF422845 | EF506218 | the conidial heads of *Aspergillus* species | USA |
| *P. penarojense* | CBS 113178 = IBT 23262 | GU981570 | GU981646 | leaf litter exposed in a litter bag | Colombia |
| *P. piscarium* | CBS 362.48 = ATCC 10482 = FRR 1075 = IFO 8111 = IMI 040032 = NRRL | GU981600 | GU981668 | culture from neotype of *Penicillium piscarium* | Netherlands |
| *P. radiatolobatum* | CBS 340.79 = VKMF-1823 | n.a. | KJ866962 | isotype of *Penicillium radiatolobatum* | Netherlands |
| *P. raistrickii* | CBS 261.33 = ATCC 10490 = FRR 1044 = IFO 6104 = IMI | AY373927 | KJ834485 | culture from neotype of *Penicillium raistrickii* | USA |
| *P. ribeum* | IBT 16537 = LSHBB100 = NRRL 1044 = NRRL 2039 = QM 1936 = VKMF-337 | DQ267916 | DQ285625 | culture from neotype of *Penicillium ribium* | Canada |
| *P. sajarovii* | CBS 277.83 = CECT 2751 = IMI 259992 | KC411724 | KJ834489 | *Secale cereale* | Spain |
| *P. scabrosum* | CBS 683.89 = FRR 2950 = IBT 3736 = IMI 285533 = DAOM 214786 | DQ267906 | DQ285610 | Unknown | Canada |
| *P. shennangjianum* | CBS 228.89 | KC411705 | KJ834491 | *Mouldy pea* | China |
| *P. simile* | CBS 129191 = ATCC MYA-4591 | FJ376592 | FJ376595 | bioaerosol sample | Italy: Castel Gandolfo, Rome |
| *P. singorense* | CBS 138211 = DTO 129H7 | KJ775671 | KJ775164 | indoor house dust | Thailand |
|  | CBS 138212 = DTO 129H8 | KJ775672 | KJ775165 | indoor house dust | Thailand |
|  | CBS 138213 = DTO 131I8 | KJ775673 | KJ775166 | indoor house dust | Thailand |
| *P. skrjabinii* | CBS 439.75 = NRRL 13055 = FRR 1945 = IMI 196528 = VKMF-1940 | GU981576 | GU981626 | leaf litter | Netherlands |
| *P. soppii* | CBS 226.28 = ATCC 10496 = FRR 2023 = IFO 7766 = IMI 040217 = MUCL | AF033488 | DQ285616 | culture from neotype of *Penicillium soppii* | USA |
| *P. swiecickii* | NRRL 2023 = QM 1964 = IBT 18220 | AF033490 | KJ834494 | culture from neotype of *Penicillium swiecickii* | USA |
| *P. vanderhammenii* | CBS 126216 = IBT 23203 | GU981574 | GU981647 | leaf litter exposed in a litter bag | Colombia |
| *P. virgatum* | CBS 114838 = BBA 65745 | AJ748692 | KJ834500 | Glycine max rhixosphere | New Caledonia:Port Laguerre |
| *P. wotroi* | CBS 118171 = IBT 23253 | GU981591 | GU981637 | leaf litter exposed in a litter bag | Colombia |
| *P. yarmokense* | CBS 410.69 = FRR 520 = IMI 140346 = VKMF-1076 | n.a. | KJ834502 | soil | Syria |
| *P. zonatum* | CBS 992.72 = ATCC 24353 | GU981581 | GU981651 | culture from neotype of Penicillium zonatum | Netherlands |
| *Coniothyrium clematidis-rectae* | CBS 507.63 | FJ515606.1 | FJ515624.1 | *Clematis sp.* | Unknown |

^1^See Table S5.

Supplementary Table 10. Related reference strains of *Albifimbria* species used for phylogenetic analysis.

| Species | Culture accession^1^ | GenBank accession | | Host/substrate | Origin |
| --- | --- | --- | --- | --- | --- |
|  |  | ITS | TUB2 |  |  |
| *Albifimbria lateralis* | CBS 117712 | KU845881.1 | KU845957.1 | Unknown | USA |
| *Dimorphiseta terrestris* | CBS 127345 | KU846314.1 | KU846431.1 | soil | USA |
| *Xepicula leucotricha* | CBS 278.78 | KU847253.1 | KU847343.1 | soil | Colombia |
| *Xenomyrothecium tongaense* | CBS 598.80 | KU847246.1 | KU847336.1 | *Halimeda sp.* | Tonga |
| *Dimorphiseta obtusa* | LXSJ38 | MH793283.1 | MH793309.1 | soil | China |
| *Isaria japonica* | BCC 2787 | AY624200.1 | EF411251.1 | insect, Lepidoptera | China |
| *Beauveria bassiana* | MRCIF40 | EU573329.1 | EU604134.1 | *Abies alba* | Poland |
| *Albifimbria verrucaria* | HMCC4 | MH754508.1 | MH754511.1 | seed | China |
| *Alfaria ossiformis* (Outgroup) | CBS 324.54 | MH857348.1 | KU846015.1 | soil | USA |
| *Myrothecium leucotrichum* | [BBA 71014](https://www.ncbi.nlm.nih.gov/nuccore/MH870020.1) | AJ302000.1 | n.a. | Unknown | Germany |
| *Xepicula jollymannii* | CBS 276.48 | MH856342.1 | n.a. | Unknown | Malawi |
| *Xepicula crassiseta* | CBS 392.71 | MH860179.1 | KU847337.1 | soil | Spain |
| *Myrothecium setiramosum* | CBS 534.88 | AY254156.1 | n.a. | Unknown | Spain |
| *Myrothecium cylindrosporumense* | MFLUCC 11-0392 | KP744448.1 | n.a. | Unknown | Thailand |
| *Myrothecium uttaraditense* | n.a. | KU940137.1 | n.a. | Unknown | Thailand |
| *Septomyrothecium maraitiense* (Outgroup) | MUCL 47202 | KY389330.1 | KY366461.1 | Unknown | Belgium |
| *Smaragdiniseta bisetosa* | CBS 459.82 | MH861514.1 | KU847319.1 | rotten bark | India |
| *Albifimbria terrestris* | CBS 126186 | MH864009.1 | KU845959.1 | soil | Namibia |
| *Albifimbria viridis* | CBS 449.71 | MH860214.1 | KU845974.1 | *Vitis vinifera* | China |
| *Myrothecium atrum* | CBS 338.97 | AY254160.1 | n.a. | Unknown | Leon |

^1^See Table S5.

Supplementary Table 11. Preparation method of conidia suspension.

| Species | Conidia suspension | References |
| --- | --- | --- |
| *Fusarium* | Each isolate was inoculated in a 250 mL culture flask containing 100 mL of potato dextrose broth (PDB) medium. The cultures were shaken on a shaker at 60 rpm for 14 d. The density of the conidial suspension was measured using a haemocytometer and the inoculated spore culture was diluted using sterile distilled water to obtain a final concentration of 1 x 10^6^ spores·mL^-1^. | Gabrekiristos et al. 2018 |
| *Alternaria alternata* | Conidia produced on V8 medium as described above were washed with sterile distilled water. The density of the conidia suspension was the same as above. | Nishikawa and Nakashima 2013 |
| *Albifimbria verrucaria* | Isolates were grown on PDA amended with streptomycin sulfate for 10 d with a 12-h photoperiod in a growth chamber at 20 to 22°C. Spore suspensions with a final spore concentration of 10^6^·mL^-1^ were prepared. | Matić et al. 2019 |
| *Aspergillus flavus* | *A. flavus* was cultured at 30°C in Rose Bengal medium (B.R. Grade, Aoboxing Biotech Co. Ltd., Beijing) and harvested from 2- to 3-day-old cultures. The culture was washed with 10 ml of 0.5% sterile Tween 20 (R.T. Grade, Biodee Co. Ltd., Beijing) in distilled water (v/v). A suspension was obtained by gentle shaking and filtering through three layers of sterile gauze to remove mycelia and sporangioconidia prior to centrifugation (TGL-16C, Anting Scientific Instrument Co. Ltd., Shanghai) at 302 (× g) for 5 min. The supernatant was discarded and the pellet was resuspended in 10 ml of sterile 0.5% Tween 20. The conidial density of *A. flavus* was measured by hemacytometer, and adjusted to a final conidial suspension 1 x 10^6^ spores·mL^-1^ by appropriate dilution. | Xiong et al. 2010 |
| *Penicillium brasilianum* | Potato dextrose (PD) liquid medium was chosen to scale up the growth of *P. brasilianum*. Three PD agar plugs (1 cm^2^) of *P. brasilianum* were aseptically transferred to Erlenmeyer flasks (500 mL) containing 125 mL of sterile liquid medium. The flasks were incubated at 25°C on a rotary shaker (120 rpm) for two d. The density of the conidia suspension was the same as above. | Bazioli et al. 2020 |
| *Phoma macrostoma* | *Phoma macrostoma* was cultured at 28°C on PDA medium with light for 5-7 d. The culture was washed with 10 ml of 0.05% sterile Tween 80 (R.T. Grade, Biodee Co. Ltd., Beijing) in distilled water (v/v), and it was adjusted to a final conidial suspension 1 x 10^6^ spores·mL^-1^ by appropriate dilution. | Srisuksam et al. 2021 |

Supplementary Table 12. These tested agents used in the experiment.

| Fungicide | CAS | Molecular weight | Molecular formula | Active ingredient content | Test concentration (mg·L^-1^) | Dosage form | Producer |
| --- | --- | --- | --- | --- | --- | --- | --- |
| Azoxystrobin | 131860-33-8 | 403.3875 | C_22_H_17_N_3_O_5_ | 25% | 50,25,12.5,6.25,3.125 | Suspension concentrate (SC) | Syngenta Biotechnology Co., Ltd., China |
| Prochloraz | 67747-09-5 | 376.7 | C_15_H_16_C_l3_N_3_O_2_ | 45% | 25,12.5,6.25,3.125,1.5625 | Microemulsion (ME) | Shenzhen Noposion agrochemicals Co., Ltd., China |
| Pyraclostrobin | 175013-18-0 | 387.81700 | C_19_H_18_C_l_N_3_O_4_ | 30% | 200,100,50,25,12.5 | Suspension concentrate (SC) | Zhejiang welldone chemical Co., Ltd., China |
| Difenoconazole | 119446-68-3 | 406.26 | C_19_H_17_Cl_2_N_3_O_3_ | 10% | 50,25,12.5,6.25,3.125 | water dispersible granule (WDG) | Dongguan Rui Defeng Biological Technology Co., Ltd., China |
| Flusilazole | 85509-19-9 | 315.4 | C_16_H_15_F_2_N_3_Si | 8% | 5,2.5,1.25,0.625,0.3125 | Microemulsion(ME) | Tianjin Luheng Chemical Co., Ltd., China |
| Bromothalonil | 35691-65-7 | 265.94 | C_6_H_6_Br_2_N_2_ | 25% | 5,2.5,1.25,0.625,0.3125 | Wettable Powder (WP) | Jiangsu Tuoqiu Agrochemical Co., Ltd., China |
| Thiophanate-Methyl | 23564-05-8 | 342.394 | C_12_H_14_N_4_O_4_S_2_ | 70% | 25,12.5,6.25,3.125,1.5625 | Wettable Powder (WP) | Zhejiang welldone chemical Co., Ltd., China |
| Zineb | 8018-01-7 | 541.075 | [C](https://www.chembk.com/cn/search/C4H7N2S4Zn)_[4](https://www.chembk.com/cn/search/C4H7N2S4Zn)_[H](https://www.chembk.com/cn/search/C4H7N2S4Zn)_[7](https://www.chembk.com/cn/search/C4H7N2S4Zn)_[N](https://www.chembk.com/cn/search/C4H7N2S4Zn)_[2](https://www.chembk.com/cn/search/C4H7N2S4Zn)_[S](https://www.chembk.com/cn/search/C4H7N2S4Zn)_[4](https://www.chembk.com/cn/search/C4H7N2S4Zn)_[Zn](https://www.chembk.com/cn/search/C4H7N2S4Zn) | 80% | 25,12.5,6.25,3.125,1.5625 | Wettable Powder (WP) | Shandong Weifang Pesticide Testing Plant, China |
| Carbendazim | 10605-21-7 | 191.2 | C_9_H_9_N_3_O_2_ | 25% | 10,5,2.5,1.25,0.625 | Wettable Powder (WP) | Xuchang Jian'an District Changsheng Daily Chemical Industry Co., Ltd., China |
| Mancozeb | 8018-01-7 | 271.2 | C_8_H_12_MnN_4_S_8_Zn | 80% | 5,2.5,1.25,0.625,0.3125 | Wettable Powder (WP) | Tianjin Luheng Chemical Co., Ltd., China |
| Chlorothalonil | 1897-45-6 | 265.91 | C_8_N_2_Cl_4_ | 75% | 25,12.5,6.25,3.125,1.5625 | Wettable Powder (WP) | Shenzhen Noposion agrochemicals Co., Ltd., China |

Supplementary Table 13. Selected physico-chemical properties of soil associated with healthy trees without obvious symptoms of apple replant disease (ARD) and trees with symptoms attributed to ARD^a^

| Physico-chemical property | Soil pH | | | Soil bulk density (g·cm^-3^) | | | Soil moisture content (%) | | |
| --- | --- | --- | --- | --- | --- | --- | --- | --- | --- |
| Location numberb | ARD symptomatic | Healthy | *P* value | ARD symptomatic | Healthy | *P* value | ARD symptomatic | Healthy | *P* value |
| DC | 5.62 (0.02) | 5.62 (0.01) | 0.7953 | 1.05 (0.04) | 1.05 (0.03) | 0.9931 | 6.36 (1.22) | 6.36 (0.70) | 0.9969 |
| QC | 7.14 (0.04) | 7.10 (0.03) | 0.3769 | 1.16 (0.10) | 1.16 (0.06) | 0.9838 | 11.45 (1.88) | 11.46 (1.08) | 0.9963 |
| LC | 7.13 (0.03) | 7.10 (0.18) | 0.4709 | 1.01 (0.14) | 0.97 (0.06) | 0.6725 | 8.87 (0.21) | 8.86 (0.17) | 0.9814 |
| MC | 5.22 (0.16) | 5.15 (0.16) | 0.7386 | 1.44 (0.14) | 1.38 (0.05) | 0.5419 | 11.69 (0.99) | 11.67 (0.58) | 0.9887 |
| JC | 7.07 (0.31) | 7.11 (0.03) | 0.8747 | 1.22 (0.05) | 1.22 (0.04) | 0.9635 | 8.68 (2.05) | 8.69 (1.18) | 0.9952 |
| PC | 5.66 (0.36) | 5.78 (0.08) | 0.5828 | 1.05 (0.07) | 1.00 (0.06) | 0.5281 | 14.66 (0.53) | 14.63 (0.31) | 0.9494 |
| YL | 5.41 (0.10) | 5.33 (0.02) | 0.4327 | 1.12 (0.03) | 1.13 (0.05) | 0.7443 | 10.37 (2.98) | 10.30 (1.70) | 0.9801 |
| DL | 5.57 (0.32) | 5.66 (0.15) | 0.6545 | 0.97 (0.08) | 0.94 (0.05) | 0.7138 | 8.66 (0.79) | 8.63 (0.48) | 0.9629 |
| HC | 5.33 (0.23) | 5.22 (0.07) | 0.5983 | 1.48 (0.22) | 1.49 (0.05) | 0.9443 | 16.79 (1.29) | 16.72 (0.67) | 0.9479 |
| H | 5.01 (0.99) | 5.62 (0.01) | 0.3444 | 1.20 (0.09) | 1.18 (0.05) | 0.8209 | 16.96 (0.39) | 16.82 (0.28) | 0.7260 |
| XQ | 6.86 (0.13) | 6.82 (0.01) | 0.6601 | 1.11 (0.03) | 1.11 (0.01) | 0.9275 | 12.12 (0.11) | 12.09 (0.00) | 0.6279 |
| XC | 6.66 (0.43) | 6.72 (0.01) | 0.8026 | 1.28 (0.06) | 1.28 (0.04) | 0.8855 | 6.20 (0.08) | 6.18 (0.07) | 0.8113 |
| XJ | 6.77 (0.48) | 6.81 (0.00) | 0.8939 | 1.3 (0.07) | 1.30 (0.03) | 0.9372 | 14.08 (0.80) | 14.10 (0.28) | 0.9672 |
| XL | 6.66 (0.18) | 6.68 (0.02) | 0.9059 | 1.21 (0.08) | 1.19 (0.04) | 0.7407 | 8.04 (0.11) | 8.00 (0.00) | 0.5214 |
| XF | 6.18 (0.36) | 6.21 (0.02) | 0.8802 | 1.19 (0.05) | 1.19 (0.02) | 0.8446 | 10.68 (1.80) | 10.97 (1.27) | 0.8691 |
| XW | 6.92 (0.09) | 6.91 (0.02) | 0.9581 | 1.06 (0.17) | 1.05 (0.08) | 0.9440 | 7.67 (0.17) | 7.68 (0.16) | 0.9784 |

^a^All values are the mean of three replicates with the standard deviation of mean given in parentheses.

^b^The specific meaning of the number can be found in Supplementary Table 1.

Supplementary Table 14. Selected physico-chemical properties of soil associated with healthy trees without obvious symptoms of apple replant disease (ARD) and trees with symptoms attributed to ARD^a^

| Physico-chemical property | Available Nitrogen (mg·kg^-1^) | | | Available Phosphorus (mg·kg^-1^) | | | Available Potassium (mg·kg^-1^) | | | Organic matter (%) | | |
| --- | --- | --- | --- | --- | --- | --- | --- | --- | --- | --- | --- | --- |
| Location numberb | ARD symptomatic | Healthy | *P* value | ARD symptomatic | Healthy | *P* value | ARD symptomatic | Healthy | *P* value | ARD symptomatic | Healthy | *P* value |
| DC | 26.58 (0.39) | 26.09 (0.15) | 0.1441 | 161.62 (0.27) | 161.20 (0.15) | 0.1230 | 101.33 (1.62) | 98.38 (2.90) | 0.3887 | 1.53 (0.04) | 1.51 (0.01) | 0.4070 |
| QC | 28.97 (0.74) | 27.93 (0.32） | 0.1225 | 82.92 (0.58) | 82.10 (0.37) | 0.1767 | 234.94 (3.78) | 223.25 (11.73) | 0.3829 | 2.00 (0.09) | 2.03 (0.02) | 0.6624 |
| LC | 28.58 (0.23) | 27.63 (0.22） | 0.0211 | 142.9 (1.01) | 143.28 (0.20) | 0.5663 | 68.67 (0.87) | 63.19 (8.83) | 0.5688 | 2.15 (0.12) | 2.09 (0.05) | 0.5048 |
| MC | 30.25 (0.27) | 27.84 (3.58） | 0.5385 | 154.43 (1.62) | 153.58 (0.36) | 0.4403 | 170.24 (6.23) | 155.37 (23.6) | 0.5671 | 1.48 (0.10) | 1.42 (0.01) | 0.3808 |
| JC | 22.58 (0.37) | 22.25 (0.36） | 0.4711 | 165.13 (1.50) | 164.16 (0.31) | 0.3492 | 166.33 (0.94) | 163.75 (2.51) | 0.3721 | 1.33 (0.06) | 1.30 (0.01) | 0.5308 |
| PC | 31.46 (0.33) | 31.08 (0.25） | 0.2875 | 212.20 (0.87) | 210.73 (0.72) | 0.1676 | 241.33 (1.05) | 236.66 (5.03) | 0.4085 | 2.06 (0.26) | 1.94 (0.04) | 0.4860 |
| YL | 28.42 (0.07) | 27.81 (0.36） | 0.1710 | 90.18 (0.10) | 89.21 (0.29) | 0.2089 | 119.18 (1.50) | 108.44 (5.03) | 0.1030 | 2.55 (0.33) | 2.36 (0.04) | 0.3687 |
| DL | 42.47 (0.65) | 41.90 (0.18） | 0.2465 | 147.23 (2.20) | 146.03 (0.08) | 0.3966 | 104.69 (1.42) | 102.57 (0.84) | 0.1445 | 3.06 (0.03) | 2.94 (0.08) | 0.2124 |
| HC | 26.45 (0.42) | 26.08 (0.10） | 0.2339 | 283.42 (2.71) | 281.41 (0.39) | 0.2805 | 164.69 (1.12) | 156.21 (13.85) | 0.5735 | 1.35 (0.12) | 1.43 (0.04) | 0.3561 |
| H | 30.38 (4.64) | 28.00 (0.20） | 0.4254 | 351.17 (1.20) | 341.58 (6.80) | 0.2330 | 147.48 (2.40) | 131.07 (10.16) | 0.1846 | 1.35 (0.12) | 1.23 (0.03) | 0.2084 |
| XQ | 23.14 (0.41) | 22.21 (0.39） | 0.1133 | 1.85 (0.12) | 1.75 (0.05) | 0.2825 | 107.28 (5.19) | 96.71 (4.19) | 0.1092 | 1.42 (0.04) | 1.65 (0.31) | 0.5023 |
| XC | 24.84 (0.54) | 24.29 (0.15） | 0.1856 | 1.24 (0.04) | 1.20 (0.02) | 0.2371 | 106.82 (3.53) | 100.90 (3.84) | 0.2445 | 1.78 (0.0) | 1.59 (0.21) | 0.4717 |
| XJ | 15.95 (0.83) | 15.55 (0.12） | 0.4589 | 2.28 (0.13) | 2.21 (0.01) | 0.3772 | 190.88 (5.01) | 175.48 (14.68) | 0.3617 | 1.68 (0.06) | 1.58 (0.10) | 0.4010 |
| XL | 27.64 (1.44) | 26.15 (0.05） | 0.1485 | 24.52 (1.28) | 23.10 (0.10) | 0.1286 | 247.81 (2.35) | 242.52 (2.22) | 0.1116 | 1.68 (0.10) | 1.60 (0.14) | 0.6273 |
| XF | 23.15 (1.26) | 22.17 (0.58） | 0.3520 | 3.13 (0.38) | 2.06 (0.06) | 0.1162 | 111.88 (1.34) | 105.09 (3.65) | 0.1429 | 1.09 (0.07) | 1.04 (0.05) | 0.4917 |
| XW | 29.15 (0.17) | 28.58 (0.58） | 0.3924 | 2.08 (0.06) | 1.98 (0.08) | 0.3076 | 254.03 (11.17) | 246.71 (0.00) | 0.3201 | 2.17 (0.07) | 2.08 (0.10) | 0.4707 |

^a^All values are the mean of three replicates with the standard deviation of mean given in parentheses.

^b^The specific meaning of the number can be found in Supplementary Table 1.

Supplementary Table 15. Selected phenolic acids in the rhizosphere soil associated with healthy trees without obvious symptoms of apple replant disease (ARD) and trees with symptoms attributed to ARD^a^

| Physico-chemical property | Catechin (mg·100g^-1^) | | | Cinnamic acid (mg·100g^-1^) | | | Ferulic acid (mg·100g^-1^) | | | Total phenolic acid content (mg·100g^-1^) | | |
| --- | --- | --- | --- | --- | --- | --- | --- | --- | --- | --- | --- | --- |
| Location numberb | ARD symptomatic | Healthy | *P* value | ARD symptomatic | Healthy | *P* value | ARD symptomatic | Healthy | *P* value | ARD symptomatic | Healthy | *P* value |
| DC | 2.44 (0.28) | 2.55 (0.21) | 0.6166 | 0.06 (0.03) | 0.05 (0.03) | 0.8012 | 0.29 (0.07) | 0.25 (0.02) | 0.4412 | 14.13 (1.02) | 11.57 (0.13) | 0.0124 |
| QC | 2.74 (0.06) | 2.61 (0.32) | 0.5497 | 0.05 (0.01) | 0.07 (0.01) | 0.0560 | 0.37 (0.01) | 0.34 (0.10) | 0.6042 | 10.75 (0.43) | 8.80 (1.06) | 0.0416 |
| LC | 1.72 (0.22) | 1.71 (0.25) | 0.9919 | 0.04 (0.01) | 0.05 (0.03) | 0.5008 | 0.20 (0.03) | 0.21 (0.05) | 0.7336 | 17.28 (0.88) | 12.95 (1.26) | 0.0082 |
| MC | 1.76 (0.05) | 1.59 (0.13) | 0.1032 | 0.03 (0.02) | 0.05 (0.03) | 0.3345 | 0.48 (0.09) | 0.37 (0.04) | 0.1218 | 21.56 (0.71) | 18.95 (0.93) | 0.0178 |
| JC | 3.67 (0.45) | 4.09 (0.46) | 0.3256 | 0.03 (0.02) | 0.06 (0.02) | 0.1282 | 0.44 (0.12) | 0.30 (0.07) | 0.1403 | 12.03 (0.94) | 10.87 (0.51) | 0.1320 |
| PC | 4.16 (0.58) | 4.24 (0.52) | 0.8659 | 0.05 (0.02) | 0.07 (0.02) | 0.1279 | 0.33 (0.01) | 0.28 (0.05) | 0.1948 | 14.38 (1.17) | 11.94 (0.64) | 0.0341 |
| YL | 2.74 (0.10) | 2.55 (0.19) | 0.2106 | 0.07 (0.03) | 0.06 (0.03) | 0.6327 | 0.79 (0.19) | 0.74 (0.28) | 0.8172 | 13.28 (0.29) | 10.52 (0.68) | 0.0028 |
| DL | 3.52 (0.36) | 3.58 (0.40) | 0.8586 | 0.05 (0.01) | 0.05 (0.03) | 0.8191 | 0.14 (0.01) | 0.25 (0.06) | 0.0524 | 10.98 (0.51) | 8.31 (0.23) | 0.0012 |
| HC | 1.38 (0.32) | 1.13 (0.03) | 0.2460 | 0.04 (0.01) | 0.05 (0.01) | 0.5893 | 0.56 (0.28) | 0.72 (0.05) | 0.3886 | 16.97 (0.10) | 15.01 (0.34) | 0.0007 |
| H | 3.25 (0.24) | 3.24 (0.31) | 0.9944 | 0.04 (0.01) | 0.05 (0.03) | 0.8579 | 0.26 (0.04) | 0.19 (0.10) | 0.3309 | 22.27 (1.45) | 17.32 (0.43) | 0.0048 |
| XQ | 3.80 (0.75) | 3.88 (0.89) | 0.9168 | 0.05 (0.01) | 0.06 (0.04) | 0.9246 | 0.29 (0.09) | 0.31 (0.08) | 0.7750 | 22.25 (1.89) | 15.34 (0.51) | 0.0036 |
| XC | 1.54 (0.31) | 1.41 (0.03) | 0.5040 | 0.04 (0.01) | 0.05 (0.02) | 0.5586 | 0.55 (0.10) | 0.65 (0.13) | 0.3603 | 8.67 (0.26) | 7.31 (0.47) | 0.0121 |
| XJ | 0.66 (0.08 | 0.54 (0.02) | 0.0764 | 0.03 (0.01) | 0.06 (0.02) | 0.0527 | 0.75 (0.06) | 0.55 (0.38) | 0.4228 | 17.37 (2.35) | 10.67 (0.684) | 0.0091 |
| XL | 2.61 (0.39) | 2.47 (0.22) | 0.6358 | 0.01 (0.00) | 0.05 (0.03) | 0.1031 | 0.52 (0.15) | 0.25 (0.04) | 0.0377 | 8.64 (0.86) | 7.00 (0.68) | 0.0615 |
| XF | 3.08 (0.24) | 2.53 (0.55) | 0.1880 | 0.03 (0.01) | 0.05 (0.03) | 0.3103 | 0.36 (0.17) | 0.31 (0.06) | 0.6452 | 16.76 (0.64) | 14.11 (0.52) | 0.0051 |
| XW | 4.84 (0.50) | 4.73 (0.48) | 0.7847 | 0.06 (0.03) | 0.06 (0.03) | 0.8512 | 0.48 (0.05) | 0.53 (0.09) | 0.4369 | 16.53 (0.25) | 12.93 (0.08) | <0.0001 |

^a^All values are the mean of three replicates with the standard deviation of mean given in parentheses.

^b^The specific meaning of the number can be found in Supplementary Table 1.

Supplementary Table 16. Statistics of fungi sequencing results of rhizosphere soil samples of the replanted orchards.

| Sample  ID | Number of valid sequences | Number of optimization sequences | Optimize the number of sequence bases | Optimize sequence GC content (%) | Optimize the average length of the sequence | Optimize the length range of sequences |
| --- | --- | --- | --- | --- | --- | --- |
| XQ1 | 47372 | 43014 | 12232321 | 46.346 | 284 | 154 --> 552 |
| XQ2 | 45434 | 42227 | 12049825 | 45.612 | 285 | 160 --> 530 |
| XQ3 | 46781 | 43779 | 12299089 | 45.924 | 281 | 226 --> 541 |
| XC1 | 49184 | 46484 | 13561075 | 44.78 | 292 | 180 --> 535 |
| XC2 | 49504 | 46244 | 13238750 | 45.165 | 286 | 215 --> 555 |
| XC3 | 48632 | 45387 | 13543607 | 43.857 | 298 | 181 --> 529 |
| XJ1 | 49268 | 46800 | 12970630 | 48.05 | 277 | 193 --> 552 |
| XJ2 | 49352 | 46510 | 12763132 | 44.24 | 274 | 199 --> 567 |
| XJ3 | 49988 | 46703 | 13150578 | 44.539 | 282 | 202 --> 519 |
| XL1 | 46433 | 42216 | 11967007 | 46.245 | 283 | 197 --> 529 |
| XL2 | 48957 | 45649 | 13609221 | 45.746 | 298 | 197 --> 540 |
| XL3 | 47752 | 42319 | 11708444 | 46.537 | 277 | 213 --> 519 |
| XF1 | 31060 | 29241 | 8310829 | 46.083 | 284 | 209 --> 529 |
| XF2 | 48612 | 46076 | 13607133 | 44.347 | 295 | 161 --> 553 |
| XF3 | 42330 | 40476 | 12010882 | 47.128 | 297 | 174 --> 537 |
| XW1 | 48042 | 45121 | 12606350 | 48.301 | 279 | 187 --> 538 |
| XW2 | 47427 | 45016 | 12302157 | 48.169 | 273 | 190 --> 538 |
| XW3 | 48060 | 43898 | 12361655 | 48.1 | 282 | 174 --> 567 |
| HC1 | 45590 | 42811 | 12067040 | 47.085 | 282 | 186 --> 553 |
| HC2 | 47773 | 45432 | 12720731 | 47.286 | 280 | 179 --> 553 |
| HC3 | 45848 | 43192 | 12028748 | 46.821 | 278 | 184 --> 553 |
| H1 | 35848 | 34627 | 11587083 | 43.821 | 335 | 184 --> 519 |
| H2 | 42829 | 40992 | 13358097 | 43.923 | 326 | 184 --> 563 |
| H3 | 43670 | 41448 | 11626451 | 45.673 | 281 | 172 --> 566 |
| DCT1 | 46493 | 44501 | 13033337 | 46.368 | 293 | 185 --> 556 |
| DCT2 | 49738 | 47629 | 14078715 | 46.03 | 296 | 187 --> 549 |
| DCT3 | 41391 | 39380 | 11771251 | 46.64 | 299 | 188 --> 547 |
| QCT1 | 45664 | 43135 | 12169899 | 45.679 | 282 | 185 --> 542 |
| QCT2 | 46780 | 43200 | 12051180 | 45.835 | 279 | 183 --> 555 |
| QCT3 | 46267 | 44045 | 12981866 | 45.626 | 295 | 183 --> 541 |
| LCT1 | 28833 | 27563 | 7574785 | 46.342 | 275 | 185 --> 531 |
| LCT2 | 45229 | 43073 | 11962480 | 47.107 | 278 | 186 --> 553 |
| LCT3 | 48552 | 45759 | 13214227 | 46.327 | 289 | 186 --> 527 |
| MCT1 | 49085 | 45593 | 12847785 | 49.767 | 282 | 183 --> 553 |
| MCT2 | 49835 | 46109 | 13167414 | 47.057 | 286 | 183 --> 558 |
| MCT3 | 45150 | 42547 | 12212993 | 45.638 | 287 | 182 --> 553 |
| JCT1 | 45742 | 41933 | 11903996 | 47.274 | 284 | 184 --> 547 |
| JCT2 | 47534 | 43875 | 12354788 | 46.625 | 282 | 188 --> 574 |
| JCT3 | 45641 | 42121 | 11800592 | 46.834 | 280 | 180 --> 564 |
| PCT1 | 48148 | 38138 | 10635762 | 45.602 | 279 | 185 --> 541 |
| PCT2 | 47699 | 42529 | 11718448 | 45.415 | 276 | 185 --> 555 |
| PCT3 | 46905 | 42308 | 11607452 | 47.16 | 274 | 185 --> 519 |
| YLT1 | 47823 | 44636 | 12999748 | 46.671 | 291 | 188 --> 553 |
| YLT2 | 42794 | 40436 | 11433576 | 46.102 | 283 | 184 --> 555 |
| YLT3 | 44995 | 43239 | 12005457 | 46.399 | 278 | 185 --> 575 |
| DLT1 | 49282 | 47333 | 13388103 | 47.012 | 283 | 186 --> 553 |
| DLT2 | 36556 | 35035 | 9940859 | 47.102 | 284 | 186 --> 546 |
| DLT3 | 46496 | 44610 | 12460208 | 46.556 | 279 | 187 --> 556 |
| HJ1 | 46190 | 43008 | 12242903 | 47.938 | 285 | 195 --> 553 |
| HJ2 | 49893 | 45886 | 12989531 | 47.789 | 283 | 188 --> 563 |
| HJ3 | 49356 | 45081 | 13338654 | 47.213 | 296 | 195 --> 563 |
| J1 | 49821 | 48089 | 13316869 | 48.027 | 277 | 172 --> 553 |
| J2 | 46405 | 44939 | 12500958 | 48.577 | 278 | 172 --> 530 |
| J3 | 48172 | 46702 | 12983354 | 47.967 | 278 | 183 --> 552 |
| DCJ1 | 45135 | 42893 | 12250866 | 46.514 | 286 | 204 --> 556 |
| DCJ2 | 43475 | 41408 | 12391261 | 45.494 | 299 | 204 --> 556 |
| DCJ3 | 43871 | 42003 | 11941007 | 46.477 | 284 | 204 --> 556 |
| QCJ1 | 47990 | 45971 | 13016137 | 46.021 | 283 | 186 --> 555 |
| QCJ2 | 47263 | 43848 | 12664863 | 45.179 | 289 | 185 --> 538 |
| QCJ3 | 45022 | 42782 | 12124714 | 45.592 | 283 | 185 --> 525 |
| LCJ1 | 47834 | 45749 | 12582280 | 46.264 | 275 | 185 --> 520 |
| LCJ2 | 46873 | 45261 | 12355739 | 46.809 | 273 | 185 --> 525 |
| LCJ3 | 46034 | 43935 | 12104908 | 46.866 | 276 | 185 --> 546 |
| MCJ1 | 49960 | 48192 | 13105895 | 49.422 | 272 | 183 --> 519 |
| MCJ2 | 49995 | 47111 | 13017093 | 48.014 | 276 | 184 --> 553 |
| MCJ3 | 47884 | 45555 | 12518116 | 47.326 | 275 | 184 --> 555 |
| JCJ1 | 46511 | 41584 | 11946551 | 46.648 | 287 | 182 --> 546 |
| JCJ2 | 48480 | 44346 | 12680693 | 46.816 | 286 | 180 --> 549 |
| JCJ3 | 48436 | 44204 | 12706887 | 47.402 | 287 | 180 --> 553 |
| PCJ1 | 47439 | 43410 | 12020800 | 44.867 | 277 | 184 --> 522 |
| PCJ2 | 48599 | 43703 | 12152241 | 46.831 | 278 | 185 --> 519 |
| PCJ3 | 48988 | 44726 | 12657647 | 46.107 | 283 | 185 --> 574 |
| YLJ1 | 47968 | 45841 | 12899715 | 46.359 | 281 | 185 --> 544 |
| YLJ2 | 45206 | 41204 | 11648302 | 45.214 | 283 | 184 --> 574 |
| YLJ3 | 44500 | 40867 | 11562168 | 45.604 | 283 | 181 --> 574 |
| DLJ1 | 49948 | 48375 | 13394096 | 45.108 | 277 | 186 --> 547 |
| DLJ2 | 45808 | 44164 | 12276979 | 45.68 | 278 | 187 --> 548 |
| DLJ3 | 47798 | 46307 | 13062359 | 46.342 | 282 | 185 --> 547 |

Supplementary Table 17. Alpha-diversity of fungal communities (mean±SD) from rhizospheric soil. Values in columns followed by the same letter are not significantly different according to Duncan test at *p* < 0.05.

| Sample ID | Coverage | Sobs | Chao | ACE | Shannon | Simpson |
| --- | --- | --- | --- | --- | --- | --- |
| DCJ | 99.80% | 451.00±15.00fghi | 508.76±32.01efg | 508.00±32.67efgh | 3.81±0.07abcd | 129.83±5.41defgh |
| DCT | 99.77% | 536.67±13.69bcde | 604.33±14.71bc | 602.27±11.13bc | 3.71±0.06abcd | 149.42±4.40abcd |
| DLJ | 99.80% | 444.00±15.00fghi | 522.92±13.46def | 526.28±12.77cdefg | 3.83±0.03abcd | 122.49±5.46fgh |
| DLT | 99.77% | 476.33±16.60efghi | 553.93±12.42bcde | 552.40±13.14bcdef | 3.89±0.04abcd | 139.02±3.59cdef |
| HJ | 99.77% | 553.33±12.14abcd | 621.85±4.88bc | 620.24±4.07b | 3.91±0.19abcd | 154.53±2.85abc |
| HC | 99.73% | 487.00±6.66cdefgh | 592.56±27.63bcd | 589.69±16.36bcd | 3.31±0.08def | 144.92±3.26bcde |
| J | 99.77% | 296.00±20.66kl | 438.24±30.6ghi | 489.93±34.52efgh | 1.96±0.08g | 95.79±6.68ijk |
| H | 99.73% | 406.67±28.61ij | 541.09±25.68cdef | 553.48±15.99bcdef | 2.74±0.43f | 119.00±9.24fgh |
| JCJ | 99.87% | 494.33±4.67cdefg | 521.93±7.15def | 522.26±5.27defg | 4.22±0.04a | 145.64±2.54bcde |
| JCT | 99.80% | 558.67±29.45abc | 624.60±44.37b | 612.19±36.38b | 4.29±0.07a | 162.32±6.44ab |
| LCJ | 99.73% | 481.00±36.50defghi | 609.12±52.46bc | 607.81±54.19b | 3.26±0.16def | 131.02±8.34defgh |
| LCT | 99.73% | 468.00±24.98efghi | 520.30±19.50def | 528.06±14.75cdefg | 3.83±0.07abcd | 126.81±7.14efgh |
| MCJ | 99.77% | 465.67±13.68efghi | 578.13±10.60bcde | 563.91±7.36bcde | 3.48±0.33bcde | 135.64±4.06cdefg |
| MCT | 99.83% | 497.67±28.18cdef | 555.04±6.29bcde | 551.84±8.49bcdef | 3.69±0.33abcd | 149.80±8.62abcd |
| PCJ | 99.83% | 414.33±12.60hij | 471.44±16.74fgh | 476.24±18.94fghi | 3.78±0.11abcd | 120.31±3.28fgh |
| PCT | 99.73% | 472.67±29.87efghi | 568.41±16.02bcde | 562.28±23.83bcde | 3.76±0.27abcd | 130.93±6.41defgh |
| QCJ | 99.73% | 616.33±24.44a | 699.41±19.76a | 706.44±26.33a | 4.21±0.10a | 162.16±5.30ab |
| QCT | 99.70% | 600.67±33.01ab | 711.99±34.05a | 709.64±24.54a | 4.04±0.32abc | 161.22±3.76ab |
| XC | 99.90% | 337.33±13.78k | 373.88±19.47i | 360.06±21.35j | 3.85±0.13abcd | 111.96±7.12hi |
| XF | 100.00% | 238.00±33.15lm | 247.93±34.10j | 243.84±33.47k | 3.40±0.29cde | 79.49±13.07jkl |
| XJ | 100.00% | 220.00±44.50m | 229.28±46.26j | 226.44±45.18k | 3.61±0.36abcde | 70.53±11.95l |
| XL | 99.90% | 413.00±20.07hij | 440.57±19.24ghi | 438.95±19.61hi | 3.75±0.13abcd | 114.45±5.98ghi |
| XQ | 100.00% | 228.33±9.35m | 241.08±12.54j | 234.99±10.69k | 3.83±0.10abcd | 76.85±7.20kl |
| XW | 99.83% | 355.00±17.79jk | 421.09±9.19hi | 413.03±8.00ij | 2.95±0.29ef | 97.51±5.53ij |
| YLJ | 99.80% | 576.00±10.97ab | 620.24±11.22bc | 617.00±6.68b | 4.17±0.16ab | 168.04±2.72a |
| YLT | 99.90% | 421.33±14.66ghij | 461.17±10.41fgh | 453.16±9.06ghi | 3.84±0.14abcd | 121.06±4.78fgh |

Supplementary Table 18. Morphological and phylogenetic identification results of isolates.

| Species | | Culture characteristics | Morphological observations | Phylogenetic analyses |
| --- | --- | --- | --- | --- |
| *Alternaria alternata* YR9 | | PDA: Fast-growing, reaching 48 and 77 mm in diameter at 25°C after 5 and 7 d, respectively; aerial hypha cottony, sometimes sparse, pale gray; reverse center black; sporulation abundant; diffusible pigment absent (Figure 7. A c). | Conidia dark to yellowish brown, broad ovoid to subsphaeroid, obclavate to long ellipsoid, 30-36 μm × 14-20 μm with 3-5 transverse and 0-1 longitudinal septa, formed in long chains, surface usually smooth (Figure 7 A, d-m). | The maximum likelihood (ML) analysis results indicated that strain YR9 had sequences similar to the *Alternaria alternata* (ITS: KY617045.1) (Figure S21). |
| *Fusarium* | *F. oxysporum* (HC131, YR15) | PDA: Fast-growing, reaching 47-50 and 78-83 mm in diameter at 25°C after 5 and 7 d, respectively; Mycelia may be floccose, abundant and range in color from white to pale violet, reverse center dark magenta pigment or no pigment at all (Figure 7 B-C, c). | Macroconidia: short to medium length, straight to slightly curved, relatively slender and thin walled; apical cell tapered and curved, sometimes with a slight hook; basal cell foot shaped to pointed. Usually 3-septate, 27-46 μm × 3-5 μm. Microconidia: oval, elliptical or kidney shaped and usually 0-septate, 5-12 μm × 2.5-3.5 μm, abundant in the aerial mycelia. Microconidia form in false heads from monophialides. Chlamydospores: Usually formed singly or in pairs, but also may be found in clusters or in short chains. May be either terminal or intercalary in aerial. Smooth or rough walled (Figure 7 B, d-m; Figure 7 C, d-l). | The maximum likelihood (ML) analysis results indicated that strain HC131 had sequences similar to the *F. oxysporum* (ITS: MF445471.1, TUB2: LC592361.1, EF-1α: MF445547.1). The maximum likelihood (ML) analysis results indicated that strain YR15 had sequences similar to the *F. oxysporum* (ITS: MK429839.1, TUB2: KX253987.1, EF-1α: KX253983.1) (Figure S19). |
|  | *F. proliferatum* (MR5) | PDA: Fast-growing, reaching 48 and 74 mm in diameter at 25°C after 5 and 7 d, respectively; convex, aerial mycelia dense, white, colony margin radial, white and light purple; reverse violet pigments in the centre, white at the margin (Figure 7 F, c). | Macroconidia: slender, almost straight, 30-52.5 μm × 2.5-4 μm, and usually 3- to 5-septate, apical cell curved, basal cell poorly developed. Microconidia: club shaped with a flattened base, 0 septate, 5-16.5 μm × 2.0-4.0 μm, abundant in the aerial mycelia, form in chains and in false heads from monophialides and polyphialides. Chlamydospores are absent Figure 7 F, d-q). | The maximum likelihood (ML) analysis results indicated that strain MR5 had sequences similar to the *F. proliferatum* (TUB2: MH398240.1, EF-1α: KX656221.1, IGS: MH398241.1) (Duan et al. 2022). |
|  | *F.solani* (Q61, HC39) | PDA: Fast-growing, reaching 58-65and 76-83mm in diameter at 25°C after 5 and 7 d, respectively; white to cream with sparse mycelium, reverse brown pigments in the centre, white at the margin (Figure 7 D-E, c). | Macroconidia: relatively wide, straight, stout and robust, xapical cell blunt and rounded, basal cell poorly developed. Microconidia: oval, ellipsoid, reniform and fusiform with 0 or 1, abundant in the aerial mycelia. Conidiogenous cells: Monophialides, often quite long. Chlamydospores: usually singly or in pairs, intercalary in the hyphae or formed terminally on short lateral branches, globose to oval in shape and smooth or rough walled (Figure 7 D-E, d-q ). | The maximum likelihood (ML) analysis results indicated that strain HC39 had sequences similar to the *F.solani* (ITS: KJ572781.1, TUB2: KJ572782.1, EF-1α:KJ572787.1). The maximum likelihood (ML) analysis results indicated that strain Q61 had sequences similar to the *F.solani* (ITS: GQ121887.1, TUB2: GQ121902.1, EF-1α: GQ121907.1) (Figure S19). |
| *Aspergillus flavus* XW23 | | PDA: Fast-growing, initially yellow at first, like powder, gradually swelling up, turning into yellow-green, turning into brown-green with time, the surface is flat, reverse slightly pale (Figure 7 G, c). | The conidiophores were uncoloured, thick walled, and coarsely roughened or pitted and were vesicle bearing, 400-1000 μm × 10-20 μm, the vesicles were subglobose, diameter 25-45 μm. The cells were either uniseriate or biseriate or both. For biseriate cells, the phialides were borne on the metuale, and, in uniseriate cells, they were attached directly to the vesicles. The metulae covered nearly the entire surface of the vesicles and radiated from the vesicles in all directions. The conidia were globose with thin walls, which were slightly roughened, diameter 3.5-6.0 μm (Figure 7 G, d-o). | The maximum likelihood (ML) analysis results indicated that strain XW23 had sequences similar to the *Aspergillus flavus* (ITS: KF434091, TUB2: KF434080) (Figure S22). |
| *Penicillium brasilianum* Q9 | | CYA: mycelia white; texture velutinous; sporulation moderately dense, conidial colour en masse dull green to greyish green. Soluble pigment was absent, and reverse pigmentation pale to pale yellow (Figure 7 H, c). | Conidiophores: broomlike asymmetric, with three branches, stipes smooth walled, 20-95 μm × 2-3μm, conidia smooth walled, connectives visible, spheroidal to subspheroidal, 2-3 μm × 2-2.5 μm, sclerotia not observed (Figure 7 H, d-p). | The maximum likelihood (ML) analysis results indicated that strain Q9 had sequences similar to the *Penicillium brasilianum* (ITS: MF039289, TUB2: MF036174) (Figure S23). |
| *Albifimbria verrucaria* XW39 | | PDA: Slow-growing, reaching 32 and 38 mm in diameter at 25°C after 5 and 7 d, respectively, abundant white aerial mycelium and dark sporodochia distributed in concentric rings, reverse rosy buff Figure 7 I, c). | Conidiogenous cells phialidic, cylindrical to allantoid, hyaline, smooth to lightly verrucose, 8-15 μm × 2-3 μm. Conidia: aseptate, smooth, hyaline, fusiform, 6-8 μm × 2-3 μm Figure 7 I, d-j). | The maximum likelihood (ML) analysis results indicated that strain XW9 had sequences similar to the *Albifimbria verrucaria* (ITS: MH754508.1, TUB2: MH754511.1) (Figure S24). |
| *Phoma macrostoma* HC139 | | PDA: Fast-growing, reaching 50 and 73 mm in diameter at 25°C after 5 and 7 d, respectively; white to olivaceous, mycelium dense, wooly, aerial, reverse white to brownish.  Corn-agar: white to brownish, mycelium felty and reverse white to black  Oat-meal agar: olivaceous gray with pannose aerial mycelia, mycelium few and reverse gray to black (Figure S18 E-G). | Conspicuous thin-walled swollen cells occur in the aerial mycelium, septate and darker in colour than the hyphae. Conidial:ellipsoid or pyriform, brownish, smooth walled, 2.5-10 μm × 1-3.5 μm. Chlamydospores were not recognized (Figure S18 H-M). | The maximum likelihood (ML) analysis results indicated that strain HC139 had sequences similar to the *Phoma macrostoma* (ITS: DQ474111.1) (Figure S20). |

Supplementary Table 19. Disease Intensity of isolated *Fusarium* strains in different replanted orchards on apple seedlings. The number represents the number of strains.

| Province | Species | Disease intensity (DI)^1^ | | | | Disease intensity (DI)^2^ | | | |
| --- | --- | --- | --- | --- | --- | --- | --- | --- | --- |
|  |  | 0~10 | 10~30 | 30~70 | 70~100 | 0~10 | 10~30 | 30~70 | 70~100 |
| DC | *Fusarium verticillioides* | - | 1 | 1 | - | - | - | 2 | - |
|  | *Fusarium solani* | - | - | 2 | - | - | - | 1 | 1 |
|  | *Fusarium incarnatum* | - | 1 | - | - | - | 1 | - | - |
|  | *Fusarium sulawense* | - | 1 | - | - | 1 | - | - | - |
|  | *Fusarium luffae* | 1 | - | - | - | - | 1 | - | - |
|  | *Fusarium oxysporum* | - | 2 | 2 | - | - | 2 | 1 | 1 |
|  | *Fusarium proliferatum* | - | 2 | 2 | - | - | 2 | - | 2 |
|  | *Fusarium chlamydosporum* | 3 | - | - | - | 3 | - | - | - |
| DL | *Fusarium verticillioides* | - | 2 | 2 | - | - | 2 | 1 | 1 |
|  | *Fusarium solani* | - | 1 | 3 | - | - | 2 | 2 | - |
|  | *Fusarium oxysporum* | 1 | 1 | 1 | 1 | 1 | - | 2 | 1 |
|  | *Fusarium proliferatum* | - | 2 | 2 | 1 | - | 1 | 3 | 1 |
|  | *Fusarium chlamydosporum* | 1 | - | - | - | 1 | - | - | - |
|  | *Fusarium equiseti* | - | 3 | - | - | - | 2 | 1 | - |
|  | *Fusarium lacertarum* | - | 1 | 1 | - | - | 2 | - | - |
|  | *Fusarium tricinctum* | 1 | - | - | - | 1 | - | - | - |
| HC | *Fusarium verticillioides* | - | 4 | 1 | - | - | 3 | 2 | - |
|  | *Fusarium solani* | - | 1 | 2 | 3 | - | - | 2 | 4 |
|  | *Fusarium incarnatum* | - | 3 | 1 | - | - | 2 | 2 | - |
|  | *Fusarium oxysporum* | 2 | 2 | 2 | 2 | 2 | 1 | 1 | 4 |
|  | *Fusarium proliferatum* | - | 2 | 3 | 3 | - | 1 | 3 | 4 |
|  | *Fusarium equiseti* | - | 1 | 2 | - | - | 2 | 1 | - |
|  | *Fusarium lacertarum* | - | 2 | - | - | 1 | 1 | - | - |
|  | *Fusarium arcuatisporum* | 3 | - | - | - | 2 | 1 | - | - |
| H | *Fusarium verticillioides* | 1 | 1 | 1 | - | - | 2 | 1 | - |
|  | *Fusarium solani* | 1 | 1 | 2 | - | - | 1 | 3 | - |
|  | *Fusarium incarnatum* | 3 | - | - | - | 2 | 1 | - | - |
|  | *Fusarium oxysporum* | 2 | 1 | 1 | - | 2 | - | 2 | - |
|  | *Fusarium proliferatum* | - | 2 | 3 | - | - | 1 | 4 | - |
|  | *Fusarium equiseti* | - | - | 1 | 1 | - | - | 2 | - |
|  | *Fusarium lacertarum* | - | 1 | - | - | - | 1 | - | - |
|  | *Fusarium arcuatisporum* | 1 | - | - | - | 1 | - | - | - |
|  | *Fusarium tricinctum* | 1 | 1 | - | - | 2 | - | - | - |
| PC | *Fusarium verticillioides* | - | 2 | - | - | - | 2 | - | - |
|  | *Fusarium solani* | - | 2 | 2 | 1 | - | 1 | 2 | 2 |
|  | *Fusarium incarnatum* | - | - | 1 | - | - | - | 1 | - |
|  | *Fusarium oxysporum* | - | 2 | 2 | 1 | - | - | 2 | 3 |
|  | *Fusarium proliferatum* | - | 3 | 3 | 2 | - | - | 4 | 4 |
|  | *Fusarium chlamydosporum* | 4 | - | - | - | 3 | 1 | - | - |
|  | *Fusarium equiseti* | 1 | 2 | 2 | - | 1 | 1 | 3 | - |
|  | *Fusarium lacertarum* | - | 2 | - | - | 1 | 1 | - | - |
|  | *Fusarium arcuatisporum* | 1 | 1 | 1 | - | 1 | 2 | - | - |
|  | *Fusarium tricinctum* | - | 2 | - | - | 2 | - | - | - |
| QC | *Fusarium verticillioides* | - | 1 | 1 | 2 | - | 1 | 3 | - |
|  | *Fusarium solani* | - | 2 | 1 | 3 | - | 1 | 1 | 4 |
|  | *Fusarium incarnatum* | - | 2 | - | - | - | 1 | 1 | - |
|  | *Fusarium sulawense* | - | 1 | 2 | - | - | 1 | 1 | 1 |
|  | *Fusarium oxysporum* | - | 3 | 1 | - | - | - | 1 | 3 |
|  | *Fusarium proliferatum* | - | 2 | 1 | 3 | - | 1 | 2 | 3 |
|  | *Fusarium chlamydosporum* | 2 | - | - | - | 2 | - | - | - |
|  | *Fusarium equiseti* | 2 | 2 | - | - | 2 | 1 | 1 | - |
|  | *Fusarium lacertarum* | 1 | - | - | - | 1 | - | - | - |
|  | *Fusarium arcuatisporum* | 1 | 1 | - | - | 1 | - | 1 | - |
| XC | *Fusarium verticillioides* | 1 | - | - | - | 1 | - | - | - |
|  | *Fusarium solani* | - | 3 | - | - | - | 1 | 1 | 1 |
|  | *Fusarium incarnatum* | 1 | 2 | - | - | 1 | 1 | 1 | - |
|  | *Fusarium sulawense* | 2 | - | - | - | 2 | - | - | - |
|  | *Fusarium oxysporum* | 1 | - | 1 | - | 1 | - | 1 | - |
|  | *Fusarium proliferatum* | - | 1 | 1 | 1 | - | 1 | 2 | - |
|  | *Fusarium chlamydosporum* | 1 | 1 | - | - | 1 | 1 | - | - |
|  | *Fusarium equiseti* | - | 2 | - | - | - | 1 | 1 | - |
|  | *Fusarium citri* | 2 | 1 | - | - | 1 | 1 | 1 | - |
|  | *Fusarium arcuatisporum* | - | 2 | - | - | 1 | 1 | - | - |
| XF | *Fusarium verticillioides* | - | - | 1 | - | - | 1 | - | - |
|  | *Fusarium solani* | - | - | 3 | 1 | - | - | 3 | 1 |
|  | *Fusarium sulawense* | - | 1 | 1 | - | - | 2 | - | - |
|  | *Fusarium oxysporum* | 1 | - | 2 | 1 | 1 | - | 1 | 2 |
|  | *Fusarium proliferatum* | - | 2 | 1 | 2 | - | 1 | 1 | 3 |
|  | *Fusarium chlamydosporum* | 2 | 1 | - | - | 3 | - | - | - |
|  | *Fusarium equiseti* | - | 2 | 1 | - | - | 1 | 1 | 1 |
|  | *Fusarium citri* | 2 | - | - | - | 1 | 1 | - | - |
|  | *Fusarium tricinctum* | 2 | - | - | - | 2 | - | - | - |
| XJ | *Fusarium verticillioides* | - | 3 | - | - | - | 1 | 2 | - |
|  | *Fusarium solan* | - | - | 2 | 4 | - | - | 2 | 4 |
|  | *Fusarium sulawense* | 1 | - | - | - | - | 1 | - | - |
|  | *Fusarium oxysporum* | 1 | - | 1 | 2 | 1 | - | 1 | 2 |
|  | *Fusarium proliferatum* | - | 2 | 1 | 3 | - | 2 | 1 | 3 |
|  | *Fusarium chlamydosporum* | 2 | - | - | - | 2 | - | - | - |
|  | *Fusarium citri* | - | 1 | 1 | - | - | 2 | - | - |
|  | *Fusarium tricinctum* | 1 | - | - | - | 1 | - | - | - |
| XL | *Fusarium verticillioides* | 1 | - | - | - | - | 1 | - | - |
|  | *Fusarium solani* | - | 3 | - | - | - | 1 | 2 | - |
|  | *Fusarium incarnatum* | 1 | - | - | - | 1 | - | - | - |
|  | *Fusarium sulawense* | 1 | - | - | - | 1 | - | - | - |
|  | *Fusarium oxysporum* | 1 | 1 | - | 1 | 1 | 1 | - | 1 |
|  | *Fusarium proliferatum* | - | 1 | 1 | 1 | - | - | 2 | 1 |
|  | *Fusarium chlamydosporum* | 3 | 2 | - | - | 4 | 1 | - | - |
|  | *Fusarium equiseti* | - | 1 | 1 | 2 | - | 1 | 1 | 2 |
|  | *Fusarium citri* | - | 1 | - | - | - | 1 | - | - |
|  | *Fusarium tricinctum* | 2 | - | - | - | 2 | - | - | - |
| XQ | *Fusarium verticillioides* | - | - | - | 1 | - | - | - | 1 |
|  | *Fusarium solani* | - | 1 | 1 | - | - | - | 2 | - |
|  | *Fusarium incarnatum* | - | 1 | - | - | - | 1 | - | - |
|  | *Fusarium sulawense* | 1 | - | - | - | 1 | - | - | - |
|  | *Fusarium oxysporum* | 2 | 1 | 1 | 1 | 2 | - | 1 | 2 |
|  | *Fusarium proliferatum* | - | 1 | 1 | 1 | - | - | 2 | 1 |
|  | *Fusarium chlamydosporum* | 2 | 1 | - | - | 3 | - | - | - |
|  | *Fusarium equiseti* | 1 | - | - | - | 1 | - | - | - |
|  | *Fusarium lacertarum* | 1 | - | - | - | - | 1 | - | - |
|  | *Fusarium ipomoeae* | - | 1 | - | - | - | 1 | - | - |
|  | *Fusarium citri* | 1 | - | - | - | 1 | - | - | - |
|  | *Fusarium arcuatisporum* | 1 | - | - | - | 1 | - | - | - |
| XW | *Fusarium verticillioides* | - | 1 | - | - | - | 1 | - | - |
|  | *Fusarium solani* | - | - | 1 | - | - | - | 1 | - |
|  | *Fusarium incarnatum* | - | 1 | - | - | 1 | - | - | - |
|  | *Fusarium sulawense* | 1 | - | - | - | 1 | - | - | - |
|  | *Fusarium oxysporum* | 1 | 1 | 1 | - | 1 | - | 2 | - |
|  | *Fusarium proliferatum* | - | 1 | 1 | 1 | - | - | 1 | 2 |
|  | *Fusarium chlamydosporum* | 1 | - | - | - | 1 | - | - | - |
|  | *Fusarium equiseti* | - | - | 1 | - | - | - | 1 | - |
|  | *Fusarium ipomoeae* | - | 1 | - | - | - | 1 | - | - |
|  | *Fusarium arcuatisporum* | 1 | - | - | - | 1 | - | - | - |
|  | *Fusarium tricinctum* | 1 | - | - | - | 1 | - | - | - |
| YL | *Fusarium verticillioides* | - | 2 | - | - | - | 1 | 1 | - |
|  | *Fusarium solani* | - | 1 | 1 | 2 | - | - | 2 | 2 |
|  | *Fusarium sulawense* | 2 | - | - | - | 1 | 1 | - | - |
|  | *Fusarium oxysporum* | 1 | - | - | 3 | 1 | - | 2 | 1 |
|  | *Fusarium proliferatum* | - | 2 | 1 | 2 | - | 1 | 2 | 2 |
|  | *Fusarium equiseti* | - | 3 | 2 | - | 1 | 2 | 2 | - |
|  | *Fusarium lacertarum* | 2 | 1 | 1 | - | 2 | 1 | 1 | - |
| JC | *Fusarium verticillioides* | 1 | - | - | - | - | 1 | - | - |
|  | *Fusarium solani* | - | 1 | 1 | 1 | - | 2 | - | 1 |
|  | *Fusarium oxysporum* | 1 | 1 | 1 | 1 | 1 | - | 2 | 1 |
|  | *Fusarium proliferatum* | - | - | 2 | 2 | - | - | 1 | 3 |
|  | *Fusarium lacertarum* | - | 2 | 1 | - | - | 3 | - | - |
|  | *Fusarium tricinctum* | 1 | - | - | - | 1 | - | - | - |
| LC | *Fusarium verticillioides* | - | 1 | 2 | - | - | 2 | 1 | - |
|  | *Fusarium solani* | - | - | 1 | 1 | - | - | - | 2 |
|  | *Fusarium oxysporum* | 1 | - | 2 | 2 | 1 | - | - | 4 |
|  | *Fusarium proliferatum* | - | - | 2 | 2 | - | - | 3 | 1 |
|  | *Fusarium lacertarum* | 1 | 2 | - | - | 1 | 2 | - | - |
|  | *Fusarium tricinctum* | 3 | - | - | - | 3 | - | - | - |
| MC | *Fusarium verticillioides* | - | 1 | 1 | - | - | - | 2 | - |
|  | *Fusarium solani* | - | - | 2 | 3 | - | - | 1 | 4 |
|  | *Fusarium oxysporum* | 3 | 1 | 1 | 2 | 3 | - | 2 | 2 |
|  | *Fusarium proliferatum* | - | 2 | 1 | 5 | - | - | 1 | 7 |
|  | *Fusarium chlamydosporum* | 4 | - | - | - | 3 | 1 | - | - |
|  | *Fusarium equiseti* | - | - | 2 | - | - | - | 2 | - |
|  | *Fusarium lacertarum* | - | 2 | - | - | - | 2 | - | - |
|  | *Fusarium tricinctum* | 1 | 1 | - | - | 1 | 1 | - | - |

^1^Disease intensity of isolated strains against M.9T337.

^2^Disease intensity of isolated strains against *Malus hupehensis* Rehd. seedling.

References

Bazioli JM, Fill TP, Rocha MC, Malavazi I, Rodrigues Filho E, de Medeiros LS (2020) Perylenequinones production induced by co-culturing *Setophoma sp.* and *Penicillium brasilianum*. Phytochemistry Letters 40: 76-83.

Boerema GH. *Phoma* identification manual: differentiation of specific and infra-specific taxa in culture. CABI, 2004.

Crous PW, Schumacher RK, Wingfield MJ, Lombard L, Giraldo A, Christensen M, Gardiennet A, Nakashima C, Pereira OL, Smith AJ (2015) Fungal systematics and evolution: FUSE 1.

Diba K, Kordbacheh P, Mirhendi S, Rezaie S, Mahmoudi M (2007) Identification of *Aspergillus* species using morphological characteristics. Pakistan journal of medical sciences 23: 867.

Domsch KH, Gams W, Anderson TH. Compendium of soil fungi. Volume 1. Academic Press (London) Ltd., 1980.

Duan Y, Jiang W, Zhang R, Chen R, Chen X, Yin C, Mao Z (2022) *Fusarium proliferatum* f.sp. *malus domestica* causing apple replant disease in China.

Gabrekiristos E, Yesuf M, Ayana G (2018) An optimized inoculation method of Fusarium wilt (*Fusarium oxysporum* f.sp. *Cubense*) causal agent of banana wilt disease in ethiopia. Greener J Breed Crop Sci 6: 7-14.

Klich MA (2002) Identification of common *Aspergillus* species. Centraalbureau voor schimmelcultures.

Kukhar E, Smagulova A, Kiyan V (2020) Biological properties of *Phoma macrostoma* related to non-dermatophyte onychomycosis. Medical mycology case reports 27: 55-58.

Leslie JF, Summerell BA (2006) *Fusarium* Laboratory Manual. Blackwell Pub Professional 10.1002/9780470278376: 280-368.

Li K, Rouse D, German T (1994) PCR primers that allow intergeneric differentiation of ascomycetes and their application to *Verticillium* spp. Applied and Environmental Microbiology 60: 4324-31.

Liang XH, Cai YJ, Liao XR, Wu K, Wang L, Zhang DB, Meng Q (2009) Isolation and identification of a new hypocrellin A-producing strain *Shiraia sp.* SUPER-H168. Microbiological research 164: 9-17.

Liu YJ, Whelen S, Hall BD (1999) Phylogenetic relationships among ascomycetes: evidence from an RNA polymerse II subunit. Molecular biology and evolution 16: 1799-808.

Lombard L, Houbraken J, Decock C, Samson R, Meijer M, Réblová M, Groenewald JZ, Crous PW (2016) Generic hyper-diversity in Stachybotriaceae. Persoonia: Molecular Phylogeny and Evolution of Fungi 36: 156.

Lombard L, Van der Merwe N, Groenewald J, Crous PW (2015) Generic concepts in Nectriaceae. Studies in Mycology 80: 189-245.

Matić S, Gilardi G, Gullino ML, Garibaldi A (2019) Emergence of leaf spot disease on leafy vegetable and ornamental crops caused by *Paramyrothecium* and *Albifimbria* species. Phytopathology 109: 1053-61.

Mbofung GY, Hong SG, Pryor BM (2007) Phylogeny of *Fusarium oxysporum* f.sp. *lactucae* inferred from mitochondrial small subunit, elongation factor 1-α, and nuclear ribosomal intergenic spacer sequence data. Phytopathology 97: 87-98.

Mohd MH (2021) First report of *Fusarium sacchari* causing leaf blotch of orchid (*Dendrobium antennatum*) in Malaysia. Crop Protection 143: 105559.

Nishikawa J, Nakashima C (2013) Taxonomic Characterization and Experimental Host Ranges of Four Newly Recorded Species of A lternaria from J apan. Journal of Phytopathology 161: 604-16.

Nishimura S (1980) Host-specific toxins from *Alternaria alternata*. Proceedings of the Japan Academy, Series B 56: 362-66.

O’Donnell K, Kistler HC, Cigelnik E, Ploetz RC (1998) Multiple evolutionary origins of the fungus causing Panama disease of banana: concordant evidence from nuclear and mitochondrial gene genealogies. Proceedings of the National Academy of Sciences 95: 2044-49.

Sang MK, Han GD, Oh JY, Chun SC, Kim KD (2014) *Penicillium brasilianum* as a novel pathogen of onion (*Allium cepa* L.) and other fungi predominant on market onion in Korea. Crop protection 65: 138-42.

Simmons EG. Alternaria: An indentification manual. 2007.

Srisuksam C, Yodpanan P, Suntivich R, Tepboonrueng P, Wattananukit W, Jongsareejit B, Amnuaykanjanasin A (2021) The fungus *Phoma multirostrata* is a host-specific pathogen and a potential biocontrol agent for a broadleaf weed. Fungal Biology.

Thathana MG, Murage H, Abia ALK, Pillay M (2017) Morphological characterization and determination of aflatoxin-production potentials of *Aspergillus flavus* isolated from maize and soil in Kenya. Agriculture 7: 80.

Visagie C, Houbraken J, Frisvad JC, Hong SB, Klaassen C, Perrone G, Seifert K, Varga J, Yaguchi T, Samson R (2014) Identification and nomenclature of the genus Penicillium. Studies in mycology 78: 343-71.

White TJ, Bruns T, Lee S, Taylor J (1990) Amplification and direct sequencing of fungal ribosomal RNA genes for phylogenetics. PCR protocols: a guide to methods and applications 18: 315-22.

Xiong K, Liu HJ, Liu R (2010) Differences in fungicidal efficiency against *Aspergillus flavus* for neutralized and acidic electrolyzed oxidizing waters. International Journal of Food Microbiology 137: 67-75.
